# Theoretical Study of the Extent of Intersystem Crossing in the O(^3^P) + C_6_H_6_ Reaction with Experimental Validation

**DOI:** 10.1021/acs.jpclett.0c02866

**Published:** 2020-10-30

**Authors:** Carlo Cavallotti, Carlo De Falco, Luna Pratali Maffei, Adriana Caracciolo, Gianmarco Vanuzzo, Nadia Balucani, Piergiorgio Casavecchia

**Affiliations:** † Dipartimento di Chimica, Materiali e Ingegneria Chimica “Giulio Natta”, Politecnico di Milano, 20131 Milano, Italy; ‡ Dipartimento di Matematica, Politecnico di Milano, 20131 Milano, Italy; § Laboratory of Molecular Processes in Combustion, Department of Chemistry, Biology and Biotechnologies, University of Perugia, 06123 Perugia, Italy

## Abstract

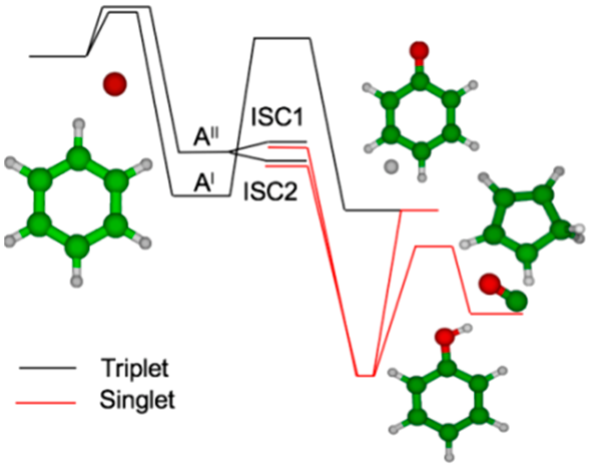

The extent of intersystem crossing in the O(^3^P) + C_6_H_6_ reaction, a prototypical system for spin-forbidden reactions in oxygenated aromatic molecules, is theoretically evaluated for the first time. Calculations are performed using nonadiabatic transition-state theory coupled with stochastic master equation simulations and Landau–Zener theory. It is found that the dominant intersystem crossing pathways connect the T2 and S0 potential energy surfaces through at least two distinct minimum-energy crossing points. The calculated channel-specific rate constants and intersystem crossing branching fractions differ from previous literature estimates and provide valuable kinetic data for the investigation of benzene and polycyclic aromatic hydrocarbons oxidation in interstellar, atmospheric, and combustion conditions. The theoretical results are supported by crossed molecular beam experiments with electron ionization mass-spectrometric detection and time-of-flight analysis at 8.2 kcal/mol collision energy. This system is a suitable benchmark for theoretical and experimental studies of intersystem crossing in aromatic species.

A detailed understanding of the mechanism of benzene oxidation in atmospheric chemistry, combustion, and astrochemistry is crucial in order to be able to interpret the reactivity of aromatic species, as benzene can be considered as the archetypical member of the family.^1−3^ An important mechanism of benzene oxidation involves intersystem crossing (ISC) between a triplet state, accessed following the addition of atomic oxygen O(^3^P) to benzene (C_6_H_6_), and the ground singlet state.^1,4^ It has indeed been shown that this is one of the main reaction routes toward benzene oxidation in combustion^5^ and that it impacts significantly the oxidation of polyaromatic hydrocarbons (PAHs).^6^ O(^3^P) addition to benzene can be rationalized in terms of two main pathways: chain branching to the phenoxy radical and atomic hydrogen and termination, leading to either cyclopentadiene (C_5_H_6_) + CO or phenol:^1,4^
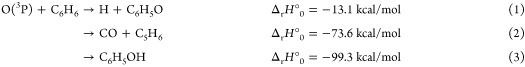
The competition between termination and branching has a huge impact on the system reactivity, as it directly impacts the concentration of radicals in the reaction environment. If termination prevails, then benzene acts as a radical sink and quenches the system reactivity. If branching is faster, then the system reactivity is catalytically enhanced. Unfortunately, while there is agreement in the literature on the global reaction rate,^7−16^ the determination of channel-specific rate constants has proven to be a major experimental and theoretical challenge. There are contrasting reports concerning the relative importance of reactions 1–3, with seminal crossed molecular beam (CMB) work suggesting that the major pathway leads to the formation of phenoxy and H (reaction 1),^17^ while a recent kinetic work suggests that in combustion conditions (1000–1200 K) the dominant pathways are chain termination reactions 2 and 3.^1^ It should be noted that, because of the complexity of the experiments,^1^ the error bars on the measured branching fractions (BFs) are rather high. Theoretically, the system reactivity was recently investigated in two studies,^1,4^ with the extent of ISC included in the models only indirectly, because of the complexity of performing an estimate of the rate of a spin-forbidden reaction involving benzene.

In this framework, the first aim of this study is to determine at a high level of accuracy channel-specific rate constants for the title reaction including explicitly, for the first time, ISC kinetics in the theoretical model. The second aim comes from the consideration that, though the theoretical and experimental investigation of spin-forbidden reactions has been a rich and active theoretical and experimental research subject in the past decades, there is a need to identify model systems that can be used to check the quality of the theoretical and experimental approaches developed to study them. The O(^3^P) + C_6_H_6_ reaction has the possibility of becoming one such model system, as benzene is the simplest aromatic species and the number of reaction channels is limited. To fulfill these aims, in the present work the reaction kinetics of O(^3^P) + C_6_H_6_ was investigated using *ab initio* transition-state theory-based master equation (AITSTME) simulations coupled with nonadiabatic transition-state theory (NA-TST).^18^ In order to determine the accuracy of the theoretical predictions, CMB experiments were performed and compared with theory. The synergistic use of AITSTME and CMBs to investigate the kinetics of reactions between unsaturated hydrocarbons and O(^3^P) has proven to be, according to our recent experience, a valuable tool, able to provide reliable qualitative and quantitative indications of the dynamics of these systems.^19−22^

The portions of the triplet and singlet C_6_H_6_O potential energy surfaces (PESs) describing the reactivity that follows O(^3^P) addition to benzene are shown in Figure 1a,b. The reactivity on the triplet PES is controlled by a limited number of reactions.^4^ The addition of O(^3^P) to benzene leads to the formation of a C_6_H_6_O adduct W1^T^ of *Cs* symmetry having two close-lying ^3^A′ and ^3^A″ electronic states (Figure 1a). The entrance well W1^T^ can then decompose to phenoxy+H, perform ISC to the singlet PES, or decompose back to reactants. Each one of these reactions can take place either on the ^3^A′ or ^3^A″ PES. Rate constants for all reactions occurring on the triplet PES were determined using the AITSTME method, as described in the Supporting Information. All DFT calculations were performed using Gaussian09,^23^ while CCSD(T) and CASPT2 calculations were performed with Molpro.^24^

**Figure 1:**
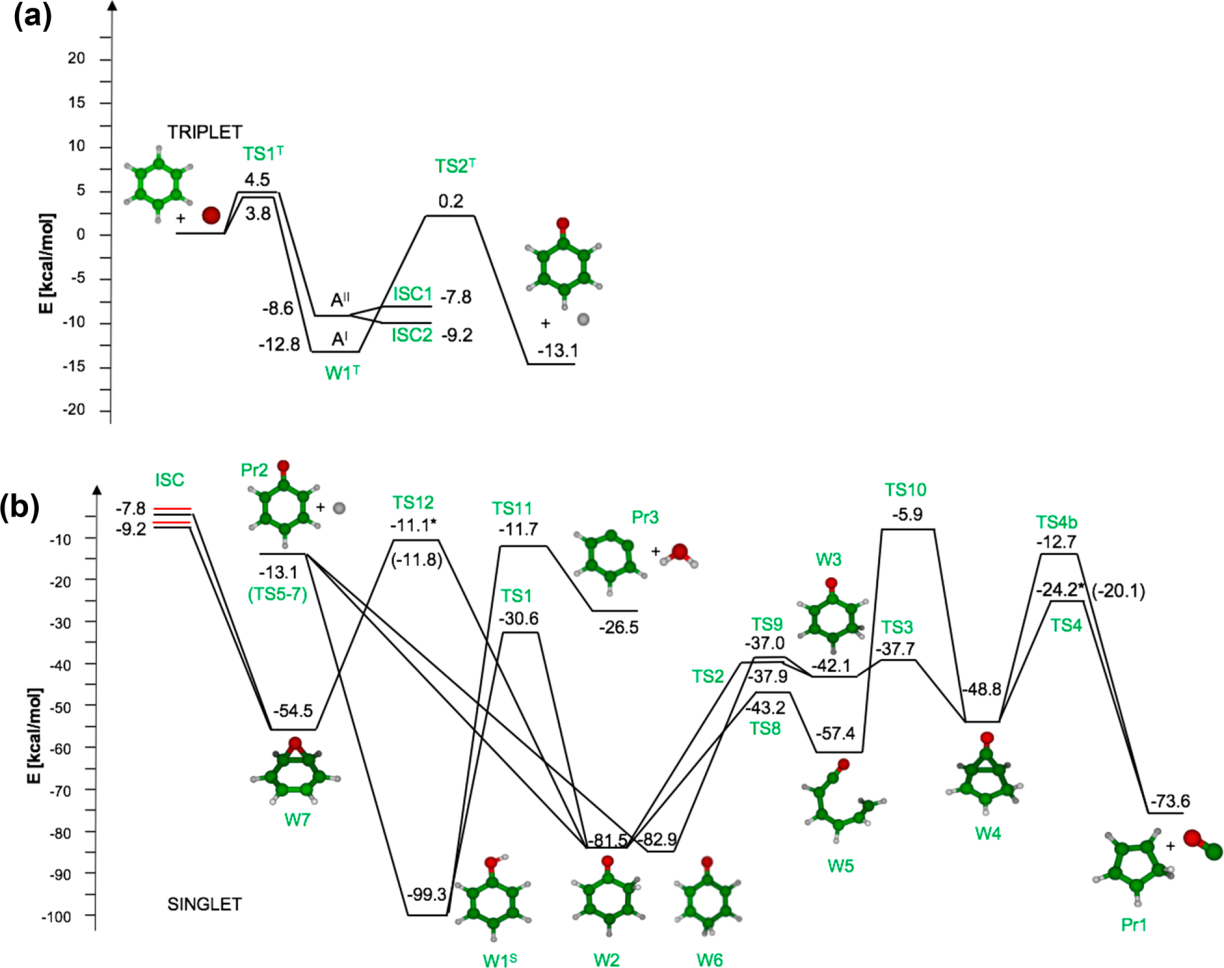
(a) Triplet potential energy surface for the O(^3^P)+C_6_H_6_ reaction with the highlighted ISC1 and ISC2 intersystem crossing pathways. (b) Singlet potential energy surface for the O(^3^P) + C_6_H_6_ reaction. Energies marked with * were computed at the CASPT2/aug-cc-pVTZ level, with CCSD(T)/CBS energies reported in parentheses.

It was found that the T1 ground state of the entrance well has ^3^A′ symmetry and that the energy gap with the T2 ^3^A″ state is 4.2 kcal/mol, thus slightly smaller than the 5.2 and 6.1–6.9 kcal/mol energy gaps determined by Nguyen et al.^4^ and Taatjes et al.,^1^ respectively. The energy barriers for O(^3^P) addition are 3.8 and 4.5 kcal/mol on the ^3^A′ and ^3^A″ PESs, respectively, thus similar to the energy barriers determined in ref (4) (4.1 and 4.3 kcal/mol) and smaller than the 5.7 kcal/mol found in ref (1).

The calculated total addition rate constant is compared with selected experimental data in Figure 2. As can be seen, it differs at the most by a factor of 1.4 at 1300 K from the experimental data of Ko et al.,^16^ while the agreement with the results of Nicovich et al.^13^ is quantitative. The agreement is good also with the total rate constants evaluated in previous theoretical studies,^1,4^ though it should be noted that in those works only the ^3^A′ PES was considered. As shown in Figure 2 we found that the contributions of reaction fluxes on the ^3^A″ PES cannot be neglected, especially at temperatures higher than 1000 K. Decomposition of W1^T^ to phenoxy + H through TS2^T^ requires overcoming a barrier of 13.0 kcal/mol on the ^3^A′ PES, while the barrier for this channel on the ^3^A″ PES is much higher^4^ and was therefore not investigated. Particular care was instead devoted to the investigation of ISC.

**Figure 2:**
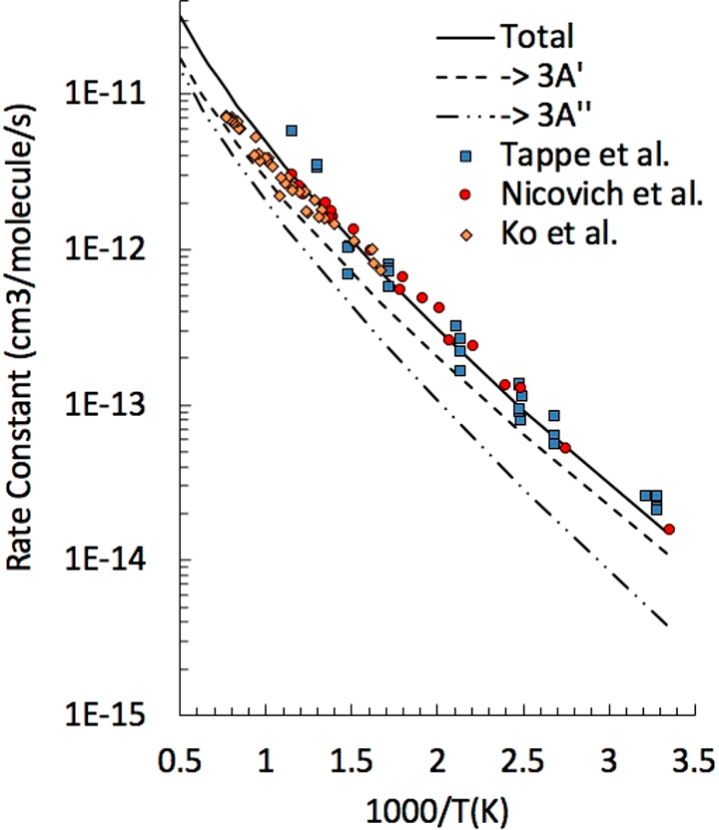
Temperature dependence of the total rate constant for the reaction of O(^3^P) addition to C_6_H_6_ calculated in this work (continuous line) compared with selected literature experimental data of Tappe et al.^15^ (■) (270–850 Torr in He), Nicovich et al.^13^ (•) (100 Torr in Ar), and Ko et al.^16^ (⧫) (180–450 Torr in Ar). The separate contributions of the ^3^A′ and ^3^A″ entrance channels are reported as dashed and dot–dashed lines, respectively.

The rate of ISC between triplet and singlet PESs was determined using NA-TST.^25−27^ According to NA-TST, ISC probabilities are computed at the minimum-energy crossing point (MECP), thus neglecting possible contributions from nonstationary points on the crossing seam. The MECPs between the triplet and singlet PESs were determined using EStokTP, an open-source software recently developed by us.^28^ As is known, the determination of MECPs between two PESs can be formulated in terms of minimization of the energy on a PES under the constraint that energies of both PESs are equal.^29^ This constrained local minimum search was performed interfacing EStokTP to NLopt,^30^ an open-source library for nonlinear optimization. Optimal performances were obtained using the SLSQP algorithm, a sequential quadratic programming (SQP) algorithm for nonlinearly constrained gradient-based optimization.^31^ MECPs were searched between both triplet ^3^A′ and ^3^A″ PESs and the singlet ground state. Spin–orbit couplings (SOCs) were evaluated using the state-interacting method at the MECPs using a Breit–Pauli Hamiltonian and a CASSCF wave function.^20^ The use of two different wave functions (restricted and unrestricted) for the singlet PES leads to the optimization of two different MECPs for the ^3^A″ state. The optimized MECP structures and the calculates SOCs are shown in Figure 3a–d. The T1/S0 structure (a) has *Cs* symmetry and a 101° O–C–H angle, characteristic of this electronic state.^1^ The calculated SOC is very small (0.6 cm^–1^), indicating that the contribution to reactivity of this pathway is negligible. The T2/S0 structures (b and c) have larger O–C–H angles and C–O distances than the T1/S0 MECP and much larger SOCs of about 35 cm^–1^. The difference in geometries and SOCs is related to the difference between the ^3^A′ and ^3^A″ electronic structures, and in particular to the different filling of the oxygen in-plane and out-of-plane lone pairs (Figure 3e,f). In the ^3^A″ state, the in-plane oxygen lone pair is doubly occupied. The resulting repulsion leads to the mentioned increase of O–C–H angle and SOC. The two T2/S0 structures differ by the symmetry, with the T2/S0 *C1* structure, determined using a restricted wave function for the singlet state, characterized by the O atom displaced from the symmetry plane toward the nearest C atom of the phenyl ring (Figure 3d). Geometric considerations suggest that the ^3^A″ triplet adduct is connected through the T2/S0 *C1* MECP (ISC2) to singlet benzene oxide (W7) and through the T2/S0 *Cs* MECP (ISC1) to a singlet diradical.

**Figure 3:**
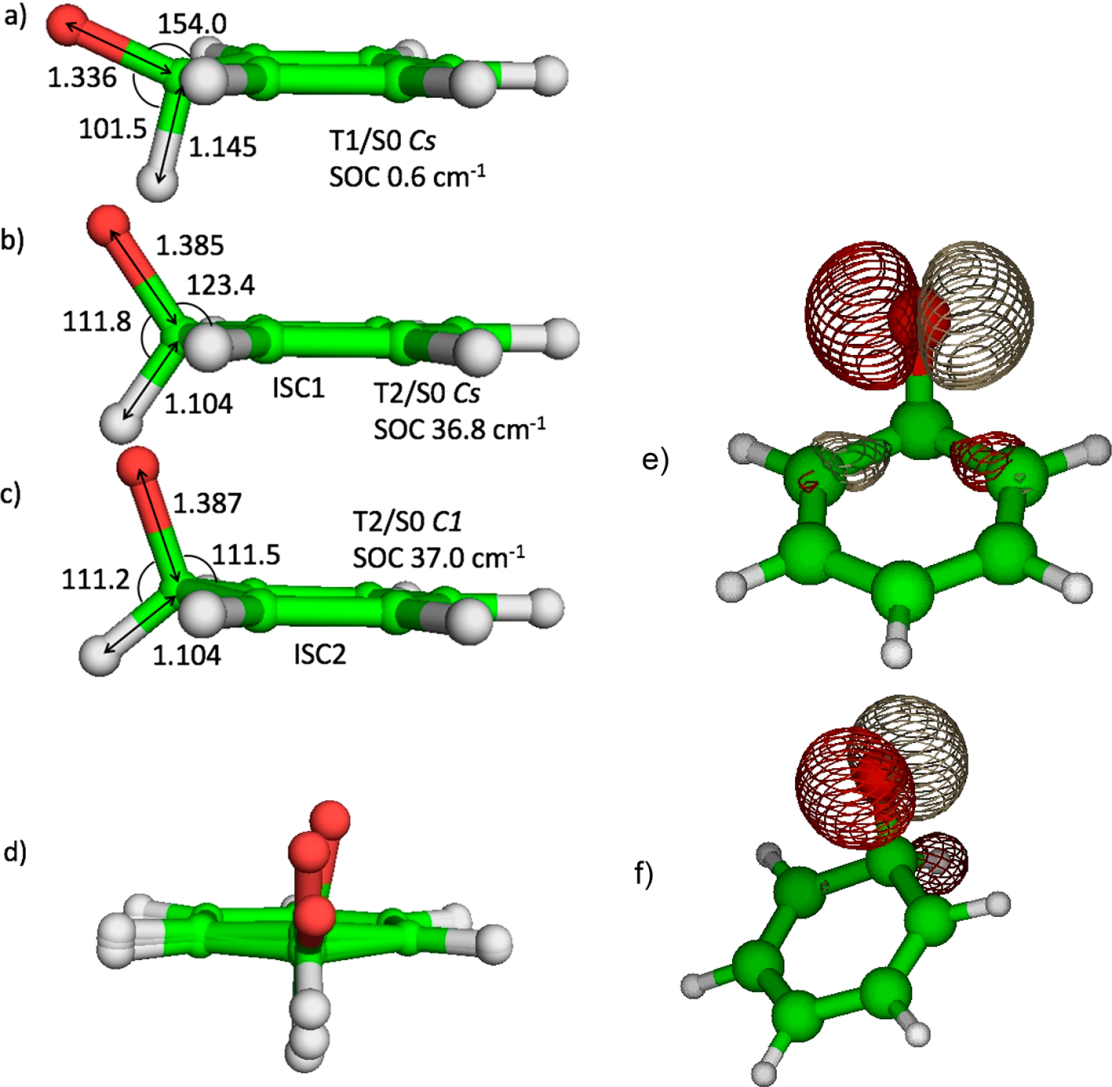
(a–c) Triplet singlet MECPs geometries and SOCs (distances in angstroms, angles in degrees). (d) Overlap of the a–c MECP geometries. (e and f) Out-of-plane a″ (e) and in-plane a′ (f) oxygen lone pair molecular orbitals calculated at the ωB97X-D/6-311+G(d,p) level for the T2/S0 geometry. In the ^3^A″ electronic state, the a′′ out-of-plane orbital is singly occupied while the in-plane a′ orbital is doubly occupied. In the ^3^A′ electronic state, the a′ and a″ orbital occupation is reversed.

The energies of ISC1 and ISC2 MECPs are 5.0 and 3.6 kcal/mol above that of W1^T^, respectively. Notably, the energy of ISC2 is slightly lower than that of the ^3^A″ W1^T^ well, which is determined by smaller ZPE corrections, while the electronic energy is, as expected, slightly higher. As both MECPs have similar SOCs, this suggests that ISC is fastest in the crossing point with lowest energy. This is, however, not the case. To understand this important point, we must consider the Landau–Zener ISC probability, *P*_LZ_
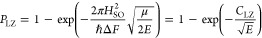
4where *H*_SO_ is the off-diagonal spin–orbit coupling element of the 4 × 4 Hamiltonian matrix between the singlet and triplet states, Δ*F* the relative slope of the triplet and singlet PES at the MECP, μ the reduced mass of the reacting moieties, and *E* kinetic energy. From the microcanonic standpoint, all the terms in eq 4 are constants, except for the kinetic energy *E*, which in NA-TST corresponds to the energy of the reaction coordinate and must be convoluted with the density of states of the other molecular degrees of freedom to determine the ISC rate constant.^25^ The *C*_LZ_ constant defined by eq 4 allows comparing ISC probabilities efficiently. They are a function of both SOC, Δ*F*, and μ. The SOC was computed as the root-mean-square average of the three coupling elements, while Δ*F*/μ^0.5^ values were determined as the difference of the norms of the gradient determined in mass-weighted Cartesian coordinates at the MECP on the triplet and singlet PESs. The *C*_LZ_ constants for ISC1 and ISC2 so determined are 0.298 and 0.078 cm^–0.5^, thus showing that, despite the higher energy, ISC from ISC1 is considerably faster than from ISC2. The reason is that the gradients in ISC2 are quite large because of the significant displacement of the geometry from its minimum-energy configuration. The *C*_LZ_ constant for the T1/S0 ISC is 7.9 × 10^–5^, which makes this pathway uncompetitive with the others. Vibrational frequencies at the MECP were computed using the Hessian suggested by Harvey et al.,^32^ after projection of rotational and translational motions, as well as of the motion along the reaction coordinate.

The reactivity on the singlet PES is more complex than on the triplet PES, even though there are only two exit channels: decomposition to phenoxy + H and to cyclopentadiene + CO. Because we have recently investigated theoretically the decomposition kinetics of phenol,^33^ we built on that phenol PES to construct the singlet PES for the present system. One important aspect is that the predictive capability of our phenol literature PES was extensively tested with good results over phenol literature experimental pyrolysis and combustion data. Upon ISC, the system evolves toward formation of benzene oxide (W7), which is then converted to 2,4 cyclohexadienone (W2), which can either isomerize to phenol (W1^s^) or decompose, after a C3C5 ring closure (W4), to cyclopentadiene and CO (Pr1) through TS4 (Figure 1b). Once formed, phenol (W1^S^) can decompose either to phenoxy + H (Pr2) or to benzyne + H_2_O (Pr3), though the latter channel is considerably slower. Further details about the reactivity on the singlet C_6_H_6_O PES can be found in our previous work.^34^

The O + C_6_H_6_ reaction kinetics was studied integrating stochastically the 1D master equation (ME) as implemented in the kinetic Monte Carlo MC-RRKM code.^34,35^ Simulations were initially performed for the flow reactor of Taatjes et al.,^1^ who measured the branching fractions (BFs) of this reaction between 300 and 900 K and between 1 and 10 Torr and then extended to the present CMB experiments. The comparison between the calculated and experimental BFs is shown in Figure 4a. As can be observed, there is good agreement between the predicted and measured phenol BF, while the yields of the C_5_H_6_ + CO and phenoxy + H channels are underestimated and overestimated, respectively. It should be noted though that the difference from the bounds of the experimental uncertainties is not large, as for example at 900 K the calculated BF for the C_5_H_6_ + CO channel is 0.19, while the experimental value is 0.33 ± 0.08. The respective values for phenoxy + H are 0.52 and 0.33 ± 0.12. Computational uncertainties, discussed in detail in the Supporting Information, may account for such discrepancies. One interesting result of the present simulations is that the phenoxy + H channel has contributions from both the triplet and singlet PES. In particular, at 4 Torr in the 700–900 K range the triplet-to-singlet ratio of the contributions to formation of phenoxy + H is about a factor of 2. The extent of ISC at 4 and 760 Torr, shown in Figure 4b as a function of temperature, is still large at 1100 K, a temperature at which benzene oxidation experiments are typically conducted.^36,37^ Because C_6_H_6_ + O is one of the most important reactions in benzene oxidation, we can conclude that this is another system for which quantum effects directly affect combustion properties, as was observed by Som et al.^38^ for the HO_2_ + HO_2_ → H_2_O_2_ + O_2_ reaction.

**Figure 4:**
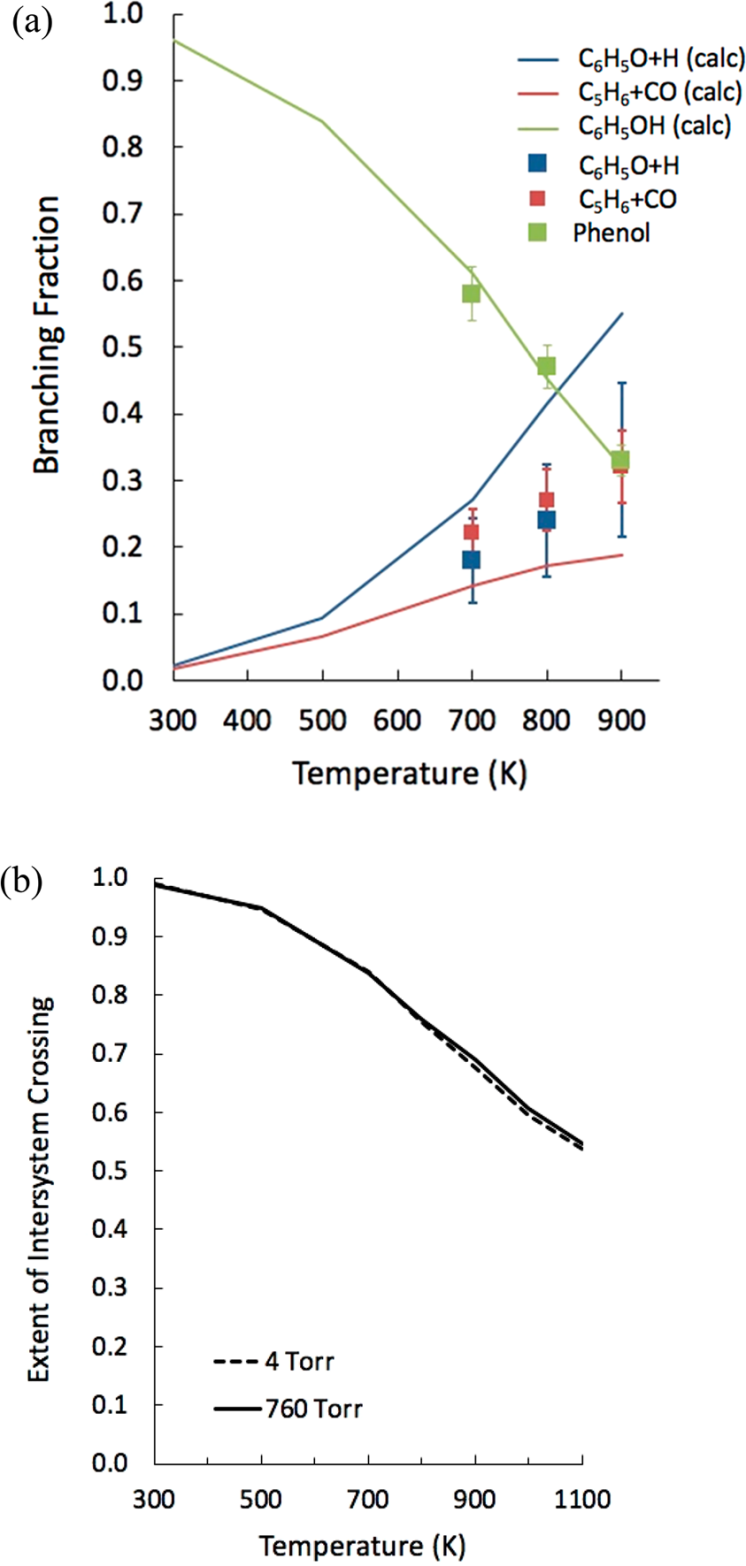
(a) Comparison between branching fractions calculated using master equation simulations at 4 Torr and 300–900 K and experimental data measured in a flow reactor.^1^ (b) Extent of ISC-calculated using ME simulations at 4 and 760 Torr in the 300–1100 K range.

In order to validate the computational model, we performed CMB experiments with mass-spectrometric detection and time-of-flight (TOF) analysis at collision energy *E*_c_ = 8.2 kcal/mol. One of the advantages of using CMB experiments is that it is possible to identify unambiguously primary products and determine BFs in a collisionless environment, as recently shown for reactions of O(^3^P) with a variety of aliphatic unsaturated hydrocarbons.^19−22,39−41^ Details of the experiment can be found in the Supporting Information. Notably, under our experimental conditions the atomic oxygen beam mainly contains O(^3^P) and a small amount (≤10%) of O(^1^D) (see the Supporting Information).^42,43^

The dynamics of channel 1, as well as that of the corresponding channel from the O(^1^D) reaction, were characterized from measurements at *m*/*z* = 93 and 65 (see the Supporting Information). Figure 5a shows the *m*/*z* = 93 angular distribution reflecting the H displacement channel 1 from O(^3^P) and O(^1^D) (see the Supporting Information). The small signal at *m*/*z* = 94, after correction for the ^13^C natural abundance of the *m*/*z* = 93 signal, is attributed to a small fraction of phenol intermediate whose lifetime is longer than its flight time from the collision region to the ionization zone of the detector (≥300 μs) and corresponds to channel 3. It is noteworthy that a small amount of phenol was also observed in the early CMB work.^17^ Phenol was probed both in angular and TOF distributions at *m*/*z* = 66 (Figure 5b). As can be seen, the *m*/*z* = 66 angular distribution exhibits a prominent peak, centered at the CM angle, superimposed to two broad wings. The central peak reflects the small amount of phenol adduct from the O(^3^P) reaction (no phenol was observed from the O(^1^D) reaction^43,44^) that fragments in the ionizer to C_5_H_6_^+^, and its distribution reflects the centroid distribution (see the Supporting Information). In contrast, the two side wings reflect the formation of the C_5_H_6_ product detected at its parent mass (66 amu) from both O(^3^P) and O(^1^D) reactions (see the Supporting Information). The C_5_H_6_ angular distribution is very broad because of linear momentum conservation (C_5_H_6_ is left by the heavy CO coproduct). C_5_H_6_ from O(^1^D) is expected to be much faster than C_5_H_6_ from O(^3^P) (because the O(^1^D) reaction is 45.3 kcal/mol more exoergic than the O(^3^P) reaction) and can easily be disentangled in TOF spectra, as can be seen in the exemplary TOF at Θ = 40° (Figure 5b). After the characterization of the center-of-mass (CM) angular, *T*(θ), and translational energy, *P*(*E*′_T_), distributions for the various contributing channels (see the Supporting Information), the relative yield of each primary product was estimated following the procedure outlined in previous work.^40^ The experimental product BFs are reported in Table 1, together with selected thermal data measured in conditions where the BF for the C_5_H_6_ + CO channel is comparable. It should be noted that phenoxy from channel 1 is partly produced from the direct adiabatic reaction of O(^3^P) on the triplet PES (BF = 0.48 ± 0.14) and partly from the nonadiabatic reaction via ISC to the singlet PES (BF = 0.18 ± 0.06) (see Figure 5a, Table 1, and the Supporting Information), with a total BF of phenoxy + H of 0.66 ± 0.20. The ME and experimental CMB results are in reasonable agreement, though the computational predictions underestimate the phenoxy + H experimental BF (falling, however, within the lower end of the experimental uncertainty). Notably, the ME simulation overestimates the experimental thermal data at 800 K. This means that any modification of the ME simulation parameters that would lead to an improvement of the agreement with CMB data would also lead to a worsening of the predictions of thermal data, thus suggesting that the present ME kinetic simulations offer a reasonable compromise for the interpretation of both sets of experimental data. The ME simulations confirm the pronounced pressure dependence found by Taatjes et al.^1^ This is mostly determined by the reactivity on the singlet PES as ISC is fast, with an average lifetime on the triplet surface of about 10 ps (7 ps at 800 K). Also, we confirm that the dominant C_6_H_6_O product at 10 Torr is phenol, as suggested by Taatjes, who could though not rule out minor contributions from other isomers. We find that at 800 K and 4 Torr about 5% and 0.8% of cyclohexadienone and benzene oxide are the main phenol isomers produced from collisional stabilization, respectively.

**Figure 5:**
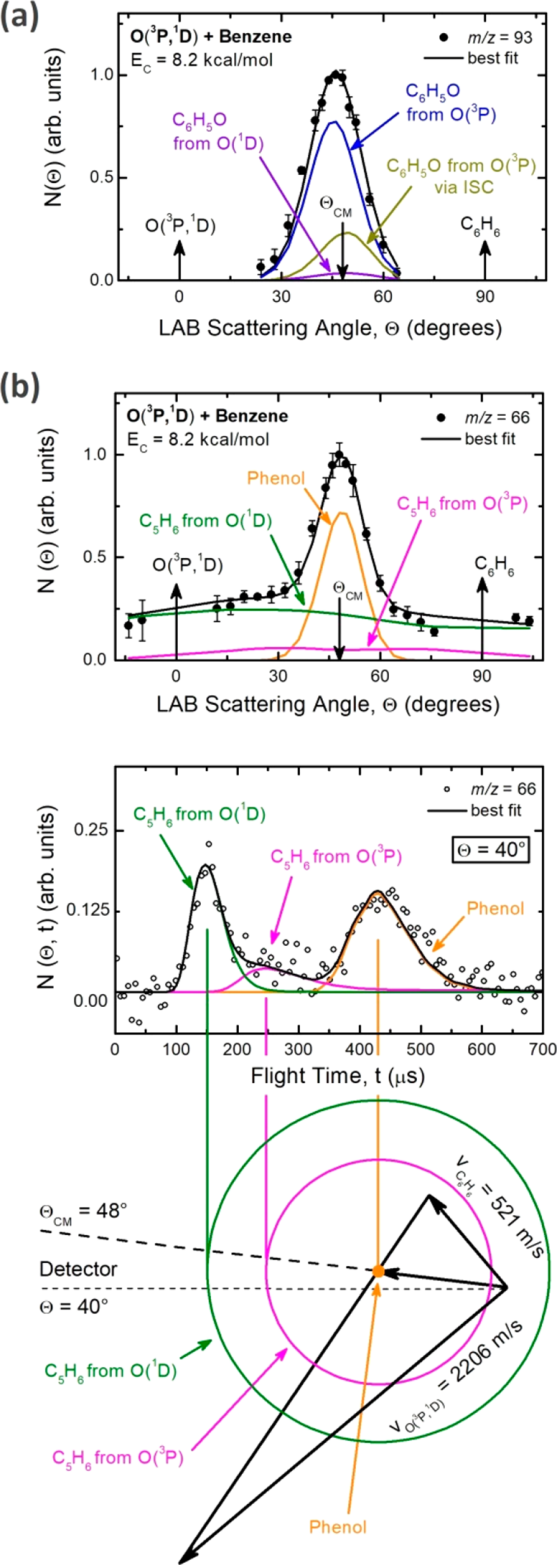
(a) Product angular distribution at *m*/*z* = 93. (b) Product angular and TOF distribution (at Θ = 40°) at *m*/*z* = 66 superimposed to the Newton diagram of the experiment for the O(^3^P,^1^D) + C_6_H_6_ reactions. Contribution from the various products are color coded and labeled, while the total best-fit is indicated with a black line (see text). The magenta and red circles in the Newton diagram represent the peak of the product velocity distribution in the CM frame for the cyclopentadiene product from O(^3^P) and O(^1^D), respectively. The phenol intermediate is centered at the CM and has zero velocity in this frame; the width of its “centroid” distribution arises from the angle and velocity spreads of the two reactant beams.

**Table 1:** History of the Branching Fractions of the O(^3^P) + C_6_H_6_ Reaction and Present Experimental and Theoretical Estimates

product channel	Sibener et al.^17^ (1980) CMB	Taatjes et al.^1^ (2010) 800 K, 4 Torr	this work ME/800 K, 4 Torr	this work CMB	this work ME/CMB
H + C_6_H_5_O	major	0.24 ± 0.10	0.40	0.66 ± 0.20	0.52
(from triplet PES)			(0.26)	(0.48 ± 0.14)	(0.22)
(from singlet PES via ISC)			(0.14)	(0.18 ± 0.06)	(0.30)
CO + C_5_H_6_ (from singlet PES via ISC)	≤5%	0.27 ± 0.06	0.16	0.32 ± 0.16	0.47
C_6_H_5_OH (from singlet PES via ISC)	small	0.47 ± 0.07	0.43	0.02 ± 0.01	0.0

In summary, the present results allow us to conclude that the O(^3^P) + C_6_H_6_ reaction is a complex reactive system that can however be consistently interpreted using ME simulations based on NA-TST. The calculated channel-specific rate constants, reported as Supporting Information, are valuable data for the investigation of the oxidation kinetics of benzene. In addition, we believe that the thorough characterization of the MECPs and ISC rates of the present work will serve as a useful reference for further theoretical calculations. Additional experimental studies would also be welcome, as it was noted that the two sets of considered experimental data are not fully compatible. Key aspects to account for in future kinetic experiments are the possible contribution of secondary reactivity. Finally, it would be interesting to investigate how a change in chemical structure of the reacting aromatic species (from benzene to naphthalene, up to larger PAHs) may impact the energy barrier for the entrance channel. A substantial decrease of the entrance barrier may in fact make this class of reactions quite relevant in astrochemical conditions and provide an efficient route for the formation of oxygenated aromatic species in interstellar environments.

## Supporting Information Available

Experimental details, computational methods, and channel-specific rate constants (PDF)
