# Supplementary material for: Theoretical Study of the Extent of Intersystem Crossing in the O(3P) + C6H6 Reaction with Experimental Validation
Source: J Phys Chem Lett. 2020 Oct 30;11(22):9621–8. doi: 10.1021/acs.jpclett.0c02866 (PMC8016199; doi:10.1021/acs.jpclett.0c02866)
Supplement: Supplementary file 1 — jz0c02866_si_001.pdf [file jz0c02866_si_001.pdf]

Electronic Supplementary Information for the paper

A Theoretical Study of the Extent of Intersystem Crossing in the $O(^3P) + C_6H_6$ Reaction with Experimental Validation

Carlo Cavallotti[1*], Carlo De Falco[2], Luna Pratali Maffei[1], Adriana Caracciolo[3], Gianmarco Vanuzzo[3], Nadia Balucani[3], and Piergiorgio Casavecchia[3*]

[1]Dipartimento di Chimica, Materiali e Ingegneria Chimica "Giulio Natta", Politecnico di Milano, 20131 Milano, Italy

[2]Dipartimento di Matematica, Politecnico di Milano, 20131 Milano, Italy

[3]Laboratory of Molecular Processes in Combustion, Department of Chemistry, Biology and Biotechnologies, University of Perugia, 06123 Perugia, Italy

This file contains:

S1. Method and experimental conditions

S2. Analysis of Experimental Results

S3. Computational Details

S4. Rate constant tables

S5. Branching fractions of collisionally stabilized singlet wells

S6. Structures, vibrational frequencies, energies, and symmetry factors of wells and saddle points

S1. Method and experimental conditions

The title reaction has been investigated under single-collision conditions using the crossed molecular beam (CMB) scattering technique with mass spectrometric (MS) detection and time-of-flight (TOF) analysis.[1-3] The basics of our CMB apparatus have been described elsewhere.[3-6] Briefly, two supersonic beams of the reactants are crossed at 90° under single-collision conditions in a large scattering chamber kept at about 2×10^{-6} mbar in operating conditions. The angular and velocity distributions of the reaction products are recorded by a triply differentially pumped, ultra-high-vacuum (UHV) (10^{-11} mbar) detector equipped with a tunable electron impact ionizer followed by a quadrupole mass filter and a Daly[7] type ion detector. The whole detector unit can be rotated in the plane of the two beams around their intersection axis (Θ=0° represents the direction of the atomic oxygen beam). The velocity of reactants and products is derived using single-shot and pseudo-random, respectively, time-of-flight (TOF) analysis. Product angular distributions were recorded by modulating the benzene beam at 160 Hz for background subtraction. In the TOF measurements of reaction products, high-time resolution was achieved by spinning the pseudorandom TOF disk (provided with four 127-bit pseudorandom sequences), located at the entrance of the detector, at 328.1 Hz (corresponding to a dwell time of 6 μs/channel). The flight length was 24.3 cm. Counting times ranged from one to four hours per angle, depending upon product mass and signal intensity.

The supersonic beam of O atoms was produced by means of a radio-frequency (RF) discharge source[8,9] operating at a RF power of 300 Watt and a gas pressure of 85 mbar of a dilute (5%) mixture of O_2 in He carrier, through a 0.48 mm diameter, water cooled quartz nozzle, followed by a 0.8 mm diameter boron nitride skimmer and a further collimating aperture. In this manner the atomic oxygen beam mainly contains O(^{3}P) and a small amount of O(^{1}D) (≤10%).[8] Peak velocity and speed ratio were 2206 m/s and 4.5, respectively. The supersonic beam of benzene was generated by expanding through a 0.1 mm diameter stainless-steel nozzle, kept at room temperature, 103 mbar of neat benzene maintained in a bath at 290 K to avoid temperature and then vapour pressure fluctuations. Beam peak velocity and speed ratio were 521 m/s and 4.2, respectively. The resulting collision energy was 8.2 kcal/mol. The small percentage of O(^{1}D) present in the atomic oxygen beam is expected to contribute significantly to the measured product distributions because the reaction cross section of O(^{3}P) with benzene is considerably

lower than that of O(^{1}D), being the O(^{3}P)+C_6H_6 reaction characterized by a very significant entrance energy barrier of about 4 kcal/mol (see Figure 1(a) in the main text), while the O(^{1}D) reaction is expected to be barrierless. Therefore, exploiting the presence of O(^{1}D) in the atomic oxygen beam, detailed information on the dynamics of also the O(^{1}D)+benzene reaction can be provided.[11]

The main, energetically allowed products of the O(^{3}P,^{1}D) + benzene reactions are the following:

O(^{3}P) + C_6H_6	$\rightarrow$	C_6H_5O + H	ΔH_0^0 = -13.1 (15.9) kcal/mol	(1a)
	$\rightarrow$	C_5H_6 + CO	ΔH_0^0 = -73.6 (74.0) kcal/mol	(2a)
	$\rightarrow$	(phenol)	ΔH_0^0 = -99.3 kcal/mol	(3)

O(^{1}D) + C_6H_6	$\rightarrow$	C_6H_5O + H	ΔH_0^0 = -58.5 (-61.3) kcal/mol	(1b)
	$\rightarrow$	C_5H_6 + CO	ΔH_0^0 = -119.0 (-119.4) kcal/mol	(2b)
	$\rightarrow$	C_5H_5 + CO + H	ΔH_0^0 = -43.2 (-43.6) kcal/mol	(4)

Above, the reported enthalpies of reaction at 0 K are those calculated in this work (in parenthesis experimental values from recommended $\Delta_f H_0^0$)[10]; C_5H_6 stands for 1,3-Cyclopentadiene, while C_5H_5 for the Cyclopentadienyl radical.

From laboratory (LAB) angular and TOF distributions at different mass-to-charge (*m/z*) ratios, product angular and translational energy distributions in the center-of-mass (CM) system have been derived for all above channels of both O(^{3}P) and O(^{1}D) reactions. The detailed experimental results, combined with those of statistical calculations for O(^{3}P) and O(^{1}D) reactions, including the role of intersystem crossing (ISC) from the O(^{3}P) reaction, will be the subject of a future, more extended publication[11]. In this Letter, we focus on the dynamics of H + C_6H_5O (phenoxy) and CO + C_5H_6 (cyclopentadiene) formation from O(^{3}P) + benzene (reaction channel (1a) and (2a), respectively), and note also the observation of a small amount of phenol intermediate (channel (3)) that survives long enough to reach the detector, by determining their branching fractions (BFs). Therefore, we present only experimental data relevant to the

observation and characterization of the above channels (see Figure 5 in main text and Figures below).

In Figure S1 we depict the velocity vector (so called Newton) diagram of the experiment, where the superimposed circles delimit the maximum velocity that the indicated bimolecular primary products can attain by assuming that all the available energy (given by $E_c-\Delta H_0^0$) is channeled into product translational energy. A possible small fraction of sufficiently long-lived phenol adduct is confined at the center of mass. In the study of this reaction using the CMB technique it is easy, for kinematic reasons[1,2], to detect the heavy products corresponding to channel (1a, 1b, and also 3), because the C_6H_5O (phenoxy radical) products are kinematically constrained in a narrow angular region around the CM angle (see Figure S1), and the fraction of possible, sufficiently long-lived intermediate C_6H_5OH (phenol - see below) will follow the centroid distribution and stick at the center-of-mass with nominally zero translational energy in the center-of-mass. In contrast, the detection of any of the two co-products from the ring breakage channels (2a), (2b), and (4) is more problematic because of angular momentum conservation arguments and dissociative ionization of channel (1a) and (1b) products. Yet, these channels can be readily observed in angular distributions and disentangled in TOF measurements as a function of scattering angle[11] (see, for example, Figure 5 in main text).

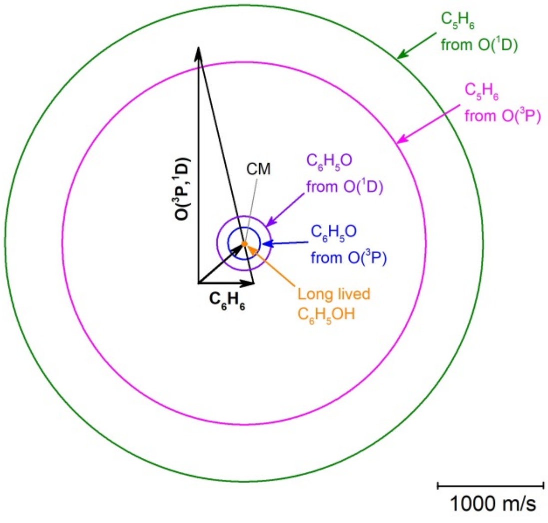

Figure S1. Velocity vector (Newton) diagram of the experiment. The radius of each circle represents the maximum velocity that the indicated product can attain in the CM system if all the available energy is channeled into product recoil energy. The phenol intermediate (the small fraction of it that has a sufficiently long lifetime to reach the detector) is centered at the CM (that is, it follows the "centroid' distribution) and has therefore zero velocity in this frame (see also Figure 5 in main text).

S2. Analysis of Experimental Results

Reactive scattering signal was registered at *m/z*= 94 (C_6H_6O), 93 (C_6H_5O), 66 (C_5H_6), and 65 (C_5H_5) with relative intensity of 0.002, 0.04, 0.06, and 1.00, respectively, when employing hard ionization detection (70 eV electron energy) (it was not necessary, neither useful to resort to soft ionization detection for this reactive system).

For the physical interpretation of the scattering data it is necessary to perform a coordinate transformation and move from the LAB frame to the CM reference frame.[1-6] The differential cross section $I_{CM}(\theta, u)$ is commonly factorized into the product of the velocity u (or translational energy E_T) distribution, P(u) (or $P(E'_T)$), and the angular distribution, $T(\theta)$: $I_{CM}(\theta, E'_T) = T(\theta) \times P(E'_T)$. In some cases the coupling between the $T(\theta)$ and $P(E'_T)$ functions needs to be accounted for, but it was not found to occur (within our sensitivity) in the present study. The best fit of the LAB product angular, $N(\Theta)$, and TOF, $N(\Theta, t)$, distributions is done by forward convoluting tentative CM distributions over the experimental conditions. In other words, the CM angular and translational energy distributions are assumed, averaged and transformed to the LAB frame for comparison with the experimental distributions, with the procedure repeated until a satisfactory fit of the experimental distributions is obtained. For reaction with multiple channels, as it is the case in the present work, if more than one product channel contributes to the signal at a given *m/z* ratio, a weighted total CM differential cross section reflecting the various possible contributions is used in the data analysis of the LAB angular and TOF distributions for a specific *m/z*, that is:

$$I_{CM}(\theta,E'_T)_{total} = \sum_i w_i \times [T(\theta) \times P(E'_T)]_i$$

with the parameter w_i, representing the relative contribution of the integral cross section of the ith channel, being a best-fit parameter.[3,4,6] The $T(\theta)$ and $P(E'_T)$ functions contain all the information about the dynamics.

The dynamics of phenoxy formation from $O(^3P)$ (channel (1a)) and from $O(^1D)$ (channel (1b)) were characterized by measuring product angular and TOF distributions at different selected angles at the parent ion *m/z*= 93 (and also at the daughter ion *m/z*=65).[11] Figure 5(a) in main text and Figure S2 (top) show that the *m/z*=93 LAB angular distribution is bell shaped and centered at

around the center of mass angle (Θ_{CM}= 48°), but with a clear forward bias, while in Figure S2 (bottom) the TOF spectrum registered at the CM angle exhibits a peak centered at around 380 μs, which is somewhat faster than the peak of the phenol (see Figure 5 of main text) because the phenol peak is centered at the CM velocity, while the peak of phenoxy, on the basis of energy and momentum conservation has a velocity somewhat different than the CM velocity, because the product translational energy distribution peaks away from zero (Fig. S3) and the phenoxy is left by an hydrogen atom co-product; in particular, the peak appears in the LAB frame at velocities larger than the CM velocity, that is, at flight times shorter than the flight time of the CM (which occurs at about 430 μs, which is where the peak of phenol occurs, as can be seen in the TOF spectrum at Θ=40° in Figure 5 of main text. We remark that the peaking of the CM occurs at about 430 μs also at Θ_{CM}=48°).

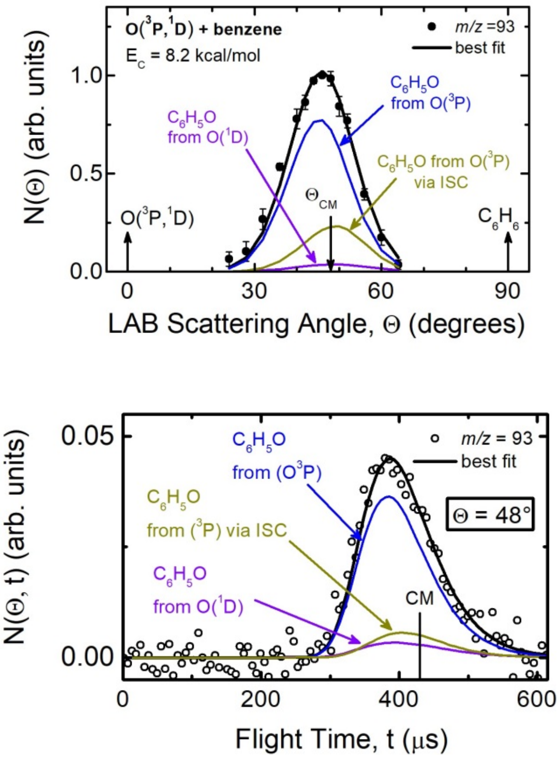

Figure S2. Top panel: LAB angular distribution (top panel) and TOF distribution (bottom panel) of *m/z*=93 product (phenoxy) from the O(^{3}P, ^{1}D) reactions with benzene at E_c=8.2 kcal/mol. Partial contributions from the two reaction are indicated, together with the total best-fit.

The primary product at *m/z*=93 was identified as C_6H_5O (phenoxy) from the H+C_6H_5O channel from both O(^{3}P) and O(^{1}D) reactions (channels (1a) and (1b)). Since the heavy product C_6H_5O for channel (1a) and the corresponding one (1b) for the O(^{1}D) reaction are kinematically constrained (due to the momentum and energy conservation laws) and therefore scattered within two small circles (Figure S1), their intensity is strongly amplified in the LAB frame (see Figure S2-top) because of the CM→LAB Jacobian transformation[1-3]. In Figure S2 the distinct contributions from the ^{3}P and ^{1}D reactions are represented as labeled color-coded curves, while the black line represents the overall best-fit (total C_6H_5O product) and this fits very well the experimental data. The corresponding best-fit CM functions for the O(^{3}P) and O(^{1}D) reactions producing phenoxy are shown in Figure S3. As can be seen the T(θ) of phenoxy from the O(^{3}P)+C_6H_6 reaction occurring on the triplet PES (see below) is strongly forward biased (in fact its partial contribution to the LAB angular distribution peaks at an angle smaller than θ_{CM} (*i.e.*, in the forward direction), while the T(θ) for the O(^{3}P) reaction proceeding via ISC and that for the

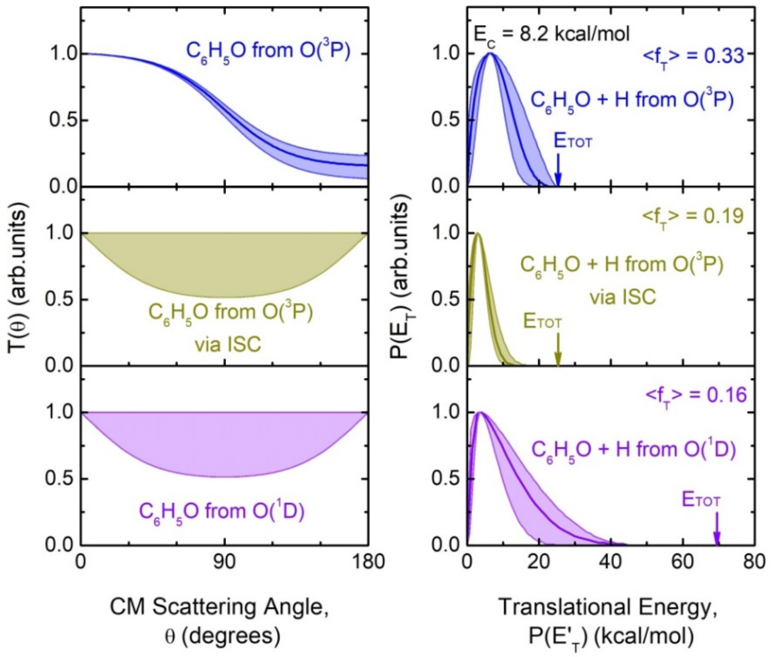

Figure S3. Left hand side: a) best-fit CM angular distributions of phenoxy (C_6H_5O) from O(^{3}P)+C_6H_6 reaction (channel (1a)). Right hand side: best fit CM translational energy distributions of the C_6H_5O + H channel from the O(^{3}P) reaction. The total available energy is indicated by the arrow and the average translational energy fraction is also given. The shaded areas represent the error bars determined for the CM functions.

$O(^1D)$ reaction are backward-forward symmetric (and to them correspond partial contributions in the LAB angular distribution centered at θ_{CM} – see Figure S2-top). The $O(^3P)$ direct contribution reflects a strongly osculating complex mechanism, while the $O(^1D)$ one and that of $O(^3P)$ via ISC reflect a long lived complex mechanism.[12]

The best-fit product translational energy distribution of the phenoxy+H channel from the $O(^3P)$ adiabatic reaction shown in Figure S3-*rhs* clearly shows that it peaks away from zero translational energy, at about 6.4 kcal/mol, corresponding to an average fraction of total available energy in product translation, $<f_T>$, of 0.33. This indicates the existence of a significant exit potential energy barrier on the way to products. Indeed, the electronic structure calculations indicate the existence of an exit barrier of about 13 kcal/mol on the triplet PES (see Figure 1(a) in main text), while no exit barrier is present on the singlet PES to the same products (see Figure 1(b) in main text) (the corresponding $P(E'_T)$ peaks at a very low energy for phenoxy+H coming from both the $O(^3P)$ reaction via ISC and the $O(^1D)$ reaction). Notably, the average fraction of total available energy released in translation is consequently much lower than for the $O(^3P)$ direct reaction (which has $<f_T>=0.33$), being 0.19 for the $O(^3P)$ reaction via ISC and 0.16 for the $O(^1D)$ reaction. We remind that the average product translational energy $<E'_T>$ is defined as $<E'_T>=\sum P(E'_T)E'_T/\sum P(E'_T)$, and the average fraction of total available energy, E_{TOT} ($E_{TOT} = E_c-\Delta H^0_0$), channeled into translation, $<f_T>$, is defined as $<f_T>=<E'_T>/E_{TOT}$.

To illustrate further the above results it should be noted that a large range of impact parameters is contributing to the reactive collisions between an $O(^3P)$ atom and a benzene molecule. For a range of impact parameters the triplet diradical intermediate can be quite short lived (≤ 1-2 rotational periods) and this contributes to the direct type forward scattering on the triplet PES. At the same time, there is also another range of impact parameters that lead to a long-lived triplet diradical intermediate that can readily undergo ISC to the corresponding singlet diradical intermediate, which can produce both C_6H_5O+H in a barrierless smooth way and/or via isomerization and ring contraction also C_5H_6 + CO (see PES in Figure 1(b) of main text). Theory suggests that the phenoxy product from $O(^3P)$ comes in part (about 45%) from a direct mechanism on the triplet PES, experiencing a significant exit energy barrier, and in part (about 55%) from a long lived complex mechanism from the singlet PES after ISC (see Table 1 in main text). Although the experiment is weakly sensitive to the fraction of phenoxy produced from

$O(^3P)$ directly from the triplet PES and that via ISC from the singlet PES, guided by theory a refined fit of the *m/z*=93 (as well as 65) data has been obtained[11] and the derived corresponding BFs are those reported in Table 1 (main text).

Let us now move to examine the angular distribution at *m/z*=66 which is characterized by a prominent peak centered at the CM angle, superimposed on two broad wings (Figure 5(b) in main text). The central peak reflects the phenol adduct from the $O(^3P)$ reaction that fragments in the ionizer to $C_5H_6^+$ (losing a CO molecule) and the measured distribution reflects that of the centroid (that is, it is determined just by the velocity and angular spreads of the two reactants beams, and the detector angular resolution). Phenol was also observed at its parent ion *m/z*=94. In fact, although at *m/z*=94 it was not possible to record the angular and velocity distributions, because of the too low signal intensity, we observed that the signal at this mass (at the center-of-mass angle) was higher than the 6,6% of the signal acquired at *m/z*= 93, attributed to the phenoxy forming channels (1a) and (1b), and corresponding to the reactive signal of the C_6H_5O isotopologue with ^{13}C natural abundance. Consequently, once subtracted the phenoxy ^{13}C contribution, the remaining *m/z*= 94 signal could only be related to the phenol adduct C_6H_5OH (indicated above as channel (3)), having a lifetime longer than its flight time from the collision region to the ionization zone (*i.e.*, ≥300 μs). In fact, it should be noted that formation of the phenol channel (3) is not possible under the single collision conditions of the present experiments, because the total energy is well above that of the possible bimolecular product channels and, therefore, the hot phenol intermediate will decompose into two product fragments. As we have discussed above, the small fraction of sufficiently long-lived phenol observed fragments very significantly in the ionizer of the MS detector by losing a CO molecule and giving a very significant ion signal at *m/z*=66. This can very clearly be seen not only in the angular distribution measured at *m/z*=66 (see Figure 5 in main text) where the peak centered at the CM angle can only originate from phenol, but also in the TOF data (Figure 5 in main text) which show that the slow peak has a flight time equal to that of the center of mass. Note that the phenol intensity is strongly amplified in the LAB frame because the C_6H_5OH adduct has nominally zero velocity in the CM frame and the mass-spectrometer is a number density detector).

In contrast, the two side wings of the product angular distribution at *m/z*=66 (Figure 5(b) in main text) reflect unambiguously the formation of CO + C_5H_6 from both $O(^3P)$ (channel (2a)) and $O(^1D)$ (channel (2b)). As can be seen the LAB angular distribution of the C_5H_6 product is very broad because of linear momentum conservation (cyclopentadiene is left by the heavy CO counter-product), and the $O(^3P)$ and $O(^1D)$ contributions to CO formation can be readily disentangled by TOF measurements. In fact, the exothermicity of the $O(^1D)$ reaction is about 45 kcal/mol larger than that of channel (2a) because of the electronic energy content (45.3 kcal/mol) of $O(^1D)$. Therefore, C_5H_6 formed from $O(^1D)$ is expected to be much faster than C_5H_6 from $O(^3P)$, as it is indeed observed experimentally (see *m/z*=66 TOF spectrum in Figure 5(b) of main text, which shows the former contribution peaking at about 150 μs and the latter at about 250 μs) being the Newton circles for the two reactions significantly different (see Figure S1). The fact that the peak attributed to C_5H_6 from $O(^1D)$ is much more intense than that from $O(^3P)$ (see Figure 5 in main text), despite the fact that the $O(^1D)$ concentration in the atomic beam is about an order of magnitude lower than that of $O(^3P)$, is due to the much larger cross section of the barrierless $O(^1D)$ reaction with respect to the $O(^3P)$ reaction, which is known to have a barrier of about 4 kcal/mol (see Figure 1(a) in the main text). Notably, although the LAB results obtained here are qualitatively similar to those obtained from an earlier pioneering CMB study[13], where a small amount of phenol was also observed peaking at the CM, the earlier data[13] were not analyzed in terms of $O(^3P)$ and $O(^1D)$ contributions (although $O(^1D)$ was known to be present in the O atom beam), because of lack of sufficient resolution (12 μs/channel TOF spectra and TOF path of about 17 cm, versus 6μs/channel and 24.3 cm of the present study).

The CO channel from $O(^3P)$ was fitted using a symmetric, slightly polarized CM angular distribution and a $P(E'_T)$ distribution peaking at about 4.7 kcal/mol and falling to zero at around 20 kcal/mol (see Figure S4), which witnesses only 8% of the total available energy released into product translation, despite the presence of a significant exit potential barrier (see Figure 1(b) in main text) (in turn, this corresponds to a very high internal excitation of the molecular CO and C_5H_6 products of about 92% of the total available energy). Note the much larger fraction (0.28) of total available energy released in translation in the $O(^1D)$ reaction (which is 45.3 kcal/mol more exothermic than the $O(^3P)$ reaction); clearly, a large fraction of the internal electronic energy of the atomic reactant is channeled into product translational energy.

After the characterization of the T(θ) and P(E'$_T$) functions for channels (1a) (Figure S3), and also (2a) (Figure S4), the relative yield of each primary product was estimated. In this work, we determined the BFs by following the procedure developed by Schmoltner *et al.*[14], and successfully applied by us in recent studies of numerous polyatomic multichannel reactions[6,15], where the parameters considered are: the apparent cross sections (w_i) derived from the best-fit analysis, the estimated electron ionization cross sections, and the measured total yield of the primary products, taking into account ion fragmentation and quadrupole transmission. The BFs for the O(^{3}P) + benzene reaction at E_c=8.2 kcal/mol are reported in Table 1 of the main text, where they are compared with the statistical predictions on the coupled triplet/singlet PESs. As can be seen in Table 1 (main text) the CMB experimental results are in good agreement with those from statistical calculations within the relative error bars.

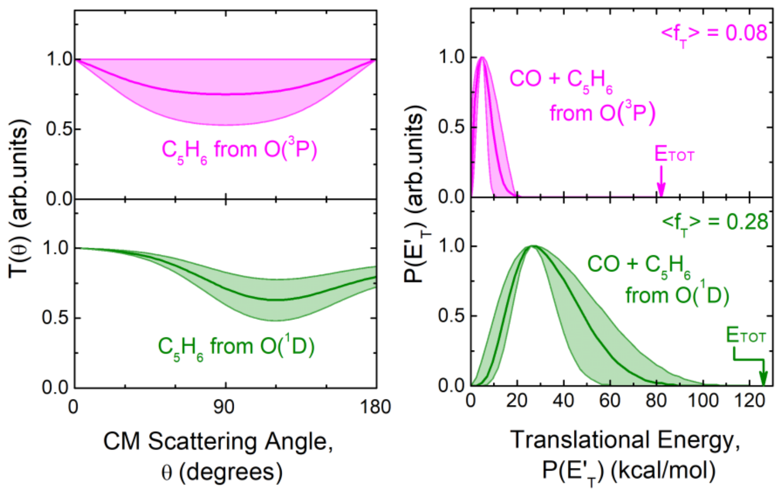

Figure S4. Left hand side: best-fit CM angular distribution determined for the C_5H_6 (cyclopentadiene) forming reactive channels originated from the contraction of the aromatic ring from the O(^{3}P) reaction (magenta) (top panel) and from the O(^{1}D) reaction (green) (bottom panel). Right hand side: best fit CM translational energy distributions of the cyc-C_5H_6 + CO channel from the O(^{3}P) reaction (channel (2a)) (top panel) and O(^{1}D) reaction (bottom panel). The total energy and the average translational energy fraction determined for each reactive channel are also indicated. The shaded areas represent the error bars determined for the CM functions.

We have found that the branching fractions of the H and CO forming channels are very different for the O(^{3}P) and O(^{1}D) reaction. In particular, in O(^{1}D) +C_6H_6 dominant is the CO + H + C_5H_5

(cyclopentadienyl) 3-body channel (4), followed by the CO + C_5H_6 channel (2b) with the H + C_6H_5O channel (1b) being minor.[11] No phenol from the O(^{1}D) reaction was detected.[11,16]

A full report on the O(^{3}P, ^{1}D) + C_6H_6 reaction dynamics, including an estimate of the relative cross sections for O(^{3}P) and O(^{1}D) reactions toward the same products, based on the relative concentration of O(^{3}P) and O(^{1}D) in the beam, will be reported in a future more extended publication.[11]

S3. Computational Details

Structures and vibrational frequencies on the triplet PES were determined using density functional theory (DFT) at the unrestricted ωB97X-D/6-311+G(d,p) level, energies of saddle points at the CASPT2/aug- cc-pVTZ level, and energies of wells at the CCSD(T)/aug-cc-pVTZ level, using DF-MP2/aug-cc-pVQZ – DF-MP2/aug-cc-pVTZ level corrections for basis size effects (referred to as CCSD(T)/CBS in the paper).

The energy difference between the $^3A'$ and $^3A''$ states of the entrance well were determined at the CASPT2/aug-cc-pVTZ level, using DFT geometries and a (10e,9o) active space, consisting of the (4e,3o) p electrons and orbitals of oxygen and of the 3 Π and Π* bonding and antibonding orbitals of benzene (6e,6o). The same active space was used to determine the energy barriers for O addition ($TS1^T$) and H loss ($TS2^T$) on the triplet surface, as well as the MECP energies. A 0.25 IPEA shift was used for all CASPT2 calculations. The use of ωB97X-D structures and CASPT2 energies has proven to be in our previous studies a computationally efficient approach for studying systems that require large active spaces to perform proper energy evaluations, with stationary point energies usually within 1 kcal/mol or better with respect to those determined using CASPT2 geometries, large active spaces, and aug-cc-pVTZ basis sets.[15,17]

A (8e,7o) active space, state averaged over two states, was used to determine the CASSCF wave function adopted to compute SOC energies. The (8e,7o) active space consisted of the two unpaired electrons and orbitals (2e,2o), of the two π and π* bonding and antibonding orbitals of benzene (4e,4o), and of the oxygen lone pair (2e,1o).

Structure and vibrational frequencies of stationary points on the singlet PES were determined at the ωB97X-D/6-311+G(d,p) level, while energies were computed at the CCSD(T)/CBS level, made exception for TS12 and TS4, that have strong multireference character. Also, barrierless channels TS5, TS6, and TS7 were studied using variational transition state theory (VTST) on PESs determined combining ωB97X-D/6-311+G(d,p) geometries and frequencies with CASPT2 energies.[17] The energy barrier of TS4 calculated at the CCSD(T)/CBS and CASPT/aug-cc-pVTZ levels differ by about 4 kcal/mol. For this specific system we tested the impact of using CASPT2(6e,6o)/aug-cc-pVTZ or ωB97X-D/6-311+G(d,p) structures to determine energies at the CASPT2(12e,1o)/aug-cc-pVTZ level. We found that the energy computed at the

CASPT2(12e,1o)/aug-cc-pVTZ//ωB97X-D/6-311+G(d,p) level is 0.5 kcal/mol smaller than that determined at the CASPT2(12e,11o)/aug-cc-pVTZ//CASPT2(6e,6o)/aug-cc-pVTZ level, thus supporting the use of unrestricted ωB97X-D geometries for multireference oxygenated aromatic systems. A (12e,11o) active space consisting of the (4e,3o) p electrons and orbitals of oxygen, of the 3 π and π* bonding and antibonding orbitals of benzene (6e,6o), and of the σ and σ* bonding and antibonding orbitals of the C-C bond of the W7 benzoxide 3 membered ring (2e,2o) was used to determine the energy of TS12.

MECPs were determined using energies and analytical gradients evaluated at the ωB97X-D/6-311+G(d,p) level using an unrestricted formalism for the triplet PES and both unrestricted and restricted formalisms for the singlet PES. Optimizations were started from the $W1^T$ structures and converged within about 50 steps, with accuracies of 10^{-10} Hartrees using 10^{-4} tolerances.

Master equation simulations were performed using a single exponential down model, with the same average collision downward energy transfer (ΔE_{down}) parameter of $260x(T/300)^{0.875}$ we used in the ME investigation of phenol decomposition.[17]

Analysis of computational uncertainties

The main computational uncertainties are related to the estimation of energy barriers of reactions with multireference character, the estimation of the rate constant for ISC, and the parameters used in the ME simulations. According to our experience with the computational methodology used in this work, energy barriers have an uncertainty smaller than 1 kcal/mol, which increases up to 2 kcal/mol for reactions with multireference character, as for example $TS2^T$ and TS4. It should be noted that for those TSs we used CASPT2 theory and large basis sets, which should limit the uncertainty.

The computational uncertainties related to the estimation of the rate of ISC crossing are determined mostly by the fact that a thorough assessment of the level of accuracy that can be reached using NA-TST theory coupled with Landau-Zener estimation of ISC probabilities to determine the rate of ISC reactions is presently missing. The many parameters that must be accurately determined to calculate the crossing rates, such as the energy barrier at the MECP, the ZPE correction, the vibrational frequencies of the MECP, the SO coupling energy, and the fact the ISC is considered only at the MECP, and not along other non stationary points on the

crossing seams, suggests that the uncertainty factor may be large (a factor of 10 or more). However, our experience in the study of the $O+C_3H_6$ reaction with NA-TST theory,[18] for which extensive experimental characterization exists, suggest that for that specific system the uncertainty of the calculated ISC rate is about a factor of 3, as larger values would lead to a very poor fit of experimental evidences. We consider this value as a reasonable uncertainty factor also for the present calculations.

Finally, there is also uncertainty about the ΔE_{down} parameter used in ME simulations. In particular it is interesting to notice that a smaller ΔE_{down} (and a higher ISC rate) would improve the agreement between the predictions of the present calculations and the C_5H_6 and H branching fractions measured by Taatjes et al.

S4. Rate Constant Table

Arrhenius fits in the form $AT^{\alpha}\exp(-E_A/RT)$. Units: cal, mol, s, cm^3. In the fitting the rate constants for all the collisionally stabilized wells (mostly W1, W2, and W7) were merged into that of phenol. Branching fractions for each well are reported in the next section.

Reaction	P [atm]	A [cm^3/mol/s]	α	E_A [cal/mol]	R^2	T Range [K]
C_6H_6+O→C_6H_5O+H						
	0.1	2.88E+08	1.68	6522	1.00	300-2200
	1	4.76E+07	1.91	6209	1.00	300-2200
	10	3.39E+08	1.66	6704	1.00	300-2200
	100	2.56E+09	1.41	7478	1.00	300-2200
C_6H_6+O→CO+C_5H_6						
	0.1	6.29E+18	-1.77	11232	0.98	300-2200
	1	1.45E+32	-5.44	24217	1.00	700-2200
	10	8.16E+34	-6.05	33005	0.98	800-2200
	100	2.89E+49	-9.85	55232	0.99	1200-2200
C_6H_6+O→C_6H_5OH						
	0.1	1.63E+16	-6.73	-14031	0.98	300-2200
	1	2.48E+14	-0.78	3522	0.98	300-2200
	10	3.01E+17	-1.58	5066	1.00	300-2200
	100	1.68E+17	-1.35	5515	1.00	300-2200
DUPLICATE						
	0.1	4.30E+35	-6.92	13025		
	1	1.03E+48	-10.40	21217		
	10	2.16E+36	-6.88	16364		
	100	6.00E+38	-7.60	18628		

S5. Branching Fractions of Collisionally Stabilized Singlet Wells

Please refer to Figure 1b for the wells nomenclature.

		**0.1 atm**	
**T [K]**	**W2**	**W1**	**W7**
300	0.21	0.55	0.24
500	0.22	0.55	0.23
700	0.24	0.61	0.15
800	0.24	0.64	0.11
900	0.23	0.69	0.07
1000	0.20	0.75	0.05
1100	0.17	0.80	0.03
1200	0.14	0.84	0.01
1300	0.10	0.89	0.01
1500	0.04	0.96	0.00
1750	0.00	0.99	0.01
2000	0.00	1.00	0.00
2200	0.00	1.00	0.00

		**1 atm**	
**T [K]**	**W2**	**W1**	**W7**
300	0.23	0.54	0.23
500	0.24	0.52	0.24
700	0.25	0.51	0.23
800	0.27	0.52	0.21
900	0.28	0.53	0.19
1000	0.28	0.57	0.15
1100	0.28	0.61	0.11
1200	0.27	0.65	0.08
1300	0.25	0.70	0.06
1500	0.18	0.80	0.02
1750	0.08	0.92	0.01
2000	0.01	0.98	0.01
2200	0.03	0.96	0.01

		**10 atm**	
**T [K]**	**W2**	**W1**	**W7**
300	0.24	0.53	0.23
500	0.24	0.52	0.25
700	0.25	0.49	0.25
800	0.26	0.49	0.25
900	0.26	0.49	0.25
1000	0.28	0.49	0.23
1100	0.29	0.50	0.21
1200	0.30	0.52	0.18
1300	0.31	0.53	0.16
1500	0.29	0.61	0.10
1750	0.25	0.72	0.04
2000	0.14	0.82	0.04
2200	0.08	0.89	0.03

		**100 atm**	
**T [K]**	**W2**	**W1**	**W7**
300	0.18	0.65	0.17
500	0.23	0.54	0.23
700	0.25	0.50	0.25
800	0.26	0.49	0.25
900	0.25	0.49	0.26
1000	0.27	0.48	0.25
1100	0.27	0.48	0.25
1200	0.27	0.48	0.25
1300	0.29	0.48	0.23
1500	0.32	0.49	0.19
1750	0.35	0.52	0.13
2000	0.37	0.55	0.08
2200	0.28	0.67	0.05

S.6 Structures and vibrational frequencies of wells and saddle points

All data reported in MESS format. Energies on the triplet PES referred to the reactants ($O(^3P)+C_6H_6$). Energies on the singlet PES referred to the energy of $W1^S$, see Figure 1 for energies referred to reactants and nomenclature.

TRIPLET

```
Well W1T AI
 Species
 RRHO
 Geometry[angstrom]          13
  C   0.00000  0.00000  0.00000
  C   0.00000  0.00000  1.35956
  C   1.20732  0.00000  2.09709
  C   2.44961  0.02644  1.42057
  C   2.51714  0.02711  0.06274
  C   1.27906  0.01188 -0.78889
  O   1.29299  0.98378 -1.76785
  H   3.46574  0.06972 -0.46003
  H  -0.92211  0.02249 -0.56934
  H  -0.94477  0.01235  1.89272
  H   1.18027  0.00537  3.17969
  H   3.36622  0.05882  2.00002
  H   1.30485 -0.91044 -1.42682
          Core     RigidRotor
                   SymmetryFactor   1.0000000000000000
          End
   Frequencies[1/cm]        33
  112.39   312.09   413.53   429.76   527.43   579.16   615.36   712.85   745.03   812.76
  859.06   953.14   983.32   985.53   997.48   1030.5   1074.9   1094.7   1138.0   1172.5
  1210.1   1243.2   1335.6   1390.2   1450.4   1556.3   1615.2   2757.3   3184.1   3187.3
  3207.7   3209.2   3220.0
  ZeroEnergy[kcal/mol]          -12.8
  ElectronicLevels[1/cm]               1
  0.0000000000000000      3.0000000000000000
 End
 End
!***********************************
Well W1T AII
 Species
 RRHO
 Geometry[angstrom]          13
 C   0.00000  0.00000  0.00000
 C   0.00000  0.00000  1.35781
 C   1.20361  0.00000  2.09960
 C   2.44252 -0.03679  1.41937
 C   2.51088 -0.03932  0.06332
```

C 1.27736 0.02613 -0.80696
O 1.31402 1.23801 -1.49620
H 3.46361 -0.06871 -0.45170
H -0.92605 -0.00020 -0.56235
H -0.94678 -0.01825 1.88742
H 1.17635 0.00588 3.18188
H 3.36045 -0.08320 1.99595
H 1.28262 -0.81268 -1.52303
Core RigidRotor
SymmetryFactor 1.0000000000000000
End
Frequencies[1/cm] 33
81.970 314.33 386.57 531.33 531.62 593.33 612.82 686.17 800.00 820.38
855.44 971.11 986.32 991.61 1001.5 1021.5 1029.1 1128.2 1187.7 1206.6
1328.2 1346.6 1383.5 1441.5 1559.4 1613.2 1856.8 2958.3 3184.8 3188.9
3211.1 3217.2 3223.0
ZeroEnergy[kcal/mol] -8.6
ElectronicLevels[1/cm] 1
0.0000000000000000 3.0000000000000000
End
End
!***********************************
Barrier TS1^{T} A^{I}
RRHO ! 1
Geometry[angstrom] 13
C 0.000000 0.000000 0.000000
C 0.000000 0.000000 2.798116
O 1.760580 0.000000 3.541948
H -0.335432 0.000000 3.831747
C 0.000000 1.223334 2.073490
C 0.000000 -1.223334 2.073490
C 0.000000 1.213771 0.695101
C 0.000000 -1.213771 0.695101
H 0.000000 2.156942 2.622280
H 0.000000 2.148917 0.147084
H 0.000000 -2.156942 2.622280
H 0.000000 -2.148917 0.147084
H -0.005077 0.000000 -1.084049
Core RigidRotor
SymmetryFactor 1.0
End
Frequencies[1/cm] 32
134.3314 168.6184
397.7220 406.5148 609.8332 611.0571 661.8186 731.8031
836.5094 908.3830 936.5521 995.7966 1006.4717 1013.6296
1036.0809 1046.8007 1060.4667 1172.3212 1177.4989 1204.5388
1323.1908 1372.4653 1490.9959 1498.2938 1601.0119 1626.0654
3162.2838 3190.8242 3200.3607 3210.8570 3220.4301 3224.3142

```
ZeroEnergy[kcal/mol]    3.8
  ElectronicLevels[1/cm]                  1
   0.0000000000000000       3.0000000000000000
 End
!*******************************************
      Tunneling    Eckart
      ImaginaryFrequency[1/cm]    501.5
      WellDepth[kcal/mol]         3.8
      WellDepth[kcal/mol]         16.6
 End
END
!**********************************
Barrier TS1^T A^II
RRHO          !          1
 Geometry[angstrom]          13
C 0.000000    0.000000    0.000000
C 0.000000    0.000000    1.379028
O 1.206697    0.000000    2.083685
H 2.423543   -0.026972    1.395873
C 2.443561   -0.028098    0.017456
C 1.227852    0.082554   -0.716023
C 1.253960    1.955934   -1.002435
C 3.383295   -0.061305   -0.520785
H -0.932551   -0.011350   -0.551509
H -0.939272   -0.018568    1.919910
H 1.199177    0.000362    3.167537
H 3.354195   -0.067327    1.950339
H 1.233047   -0.156462   -1.771381
             Core      RigidRotor
          SymmetryFactor   1.0
 End
    Frequencies[1/cm]          32
129.4001            179.1394
 372.0405            408.4012            605.6819
 609.4118            660.9616            719.7743
 836.7631            907.2501            969.6090
 995.3697           1008.2942           1019.1390
1040.2875            1049.5285            1065.0004
1163.7092            1185.4781            1203.9291
1285.1482            1381.4693            1492.1144
1512.4906            1602.3562            1626.4672
3189.6182            3197.5914            3203.9742
3215.2383            3215.6807            3225.2365
ZeroEnergy[kcal/mol]    4.5
  ElectronicLevels[1/cm]                  1
   0.0000000000000000       3.0000000000000000
 End
      Tunneling    Eckart
```

```
     ImaginaryFrequency[1/cm]   520.4
     WellDepth[kcal/mol]        4.5
     WellDepth[kcal/mol]        17.3
 End
 End
!******************************************
Barrier TS2^T
RRHO         !          1
 Geometry[angstrom]        13
C    0.000000    0.000000    0.000000
 C    0.000000    0.000000    2.832941
 O    0.380080    0.000000    4.054973
 H   -1.575026    0.000000    3.367213
 C    0.000000    1.244302    2.066199
 C    0.000000   -1.244302    2.066199
 C    0.000000    1.221959    0.698954
 C    0.000000   -1.221959    0.698954
 H    0.000000    2.169790    2.629325
 H    0.000000    2.153163    0.143435
 H    0.000000   -2.169790    2.629325
 H    0.000000   -2.153163    0.143435
 H    0.004460    0.000000   -1.083515
          Core     RigidRotor
        SymmetryFactor   1.0
 End
    Frequencies[1/cm]        32
163.4219           355.6104
 438.7039          479.3218          519.6993
 553.2119          595.7658          644.6148
 711.0613          773.7788          803.1741
 849.0645          921.2886          984.5149
 997.5207          999.3148         1014.5327
1098.0555         1161.8469          1176.2493
1277.1320         1335.1244          1378.7227
1439.5102         1472.7321          1560.4156
1605.8581         3190.9751          3196.8974
3212.9447         3220.0823          3223.7527
ZeroEnergy[kcal/mol]   0.2
 ElectronicLevels[1/cm]                1
  0.0000000000000000      3.0000000000000000
 End
     Tunneling   Eckart
     ImaginaryFrequency[1/cm]   1122.6
     WellDepth[kcal/mol]        13.0
     WellDepth[kcal/mol]        13.3
 End
 End
!******************************************
```

```
Barrier ISC1
RRHO         !          1
 Geometry[angstrom]         13
C  0.0000000000000000       0.0000000000000000       0.0000000000000000
 C  1.3566670000000001       0.0000000000000000      0.0
 C  2.1051590000000000       0.0000000000000000      -1.1997729999999998
 C  1.4239460000000002     -5.6719999999999996E-002  -2.4379270000000002
 C  6.9386000000001559E-002 -5.9286999999999994E-002  -2.5131880000000000
 C -0.81210399999999994     -1.8997000000000000E-002  -1.2818670000000001
 O  -1.5568560000000000       1.1516620000000000      -1.3299639999999999
 H -0.44070599999999976     -0.11403900000000000      -3.4670489999999998
 H -0.56243600000000016     -1.2139999999999997E-002  0.92550600000000005
 H   1.8817410000000001     -2.3945000000000010E-002  0.94932000000000016
 H   3.1870300000000000      2.1649999999999982E-003  -1.1705049999999997
 H   2.0009450000000002     -0.12368400000000000      -3.3544730000000000
 H  -1.4647570000000001     -0.90935299999999997      -1.2803979999999999
          Core     RigidRotor
        SymmetryFactor   1.0
 End
    Frequencies[1/cm]         32
  3263.1    3210.3    3197.1    3180.1    3163.1    2917.3    2779.3    1600.2    1567.8    1428.7
  1418.4    1365.6    1212.5    1195.9    1136.4    1041.4    1041.2    1028.1    997.51    988.73
  978.51    950.30    819.10    803.25    772.73    632.65    620.03    595.64    543.33    441.58
  349.45    73.040
ZeroEnergy[kcal/mol]    -7.8
  ElectronicLevels[1/cm]                1
  0.0000000000000000       3.0000000000000000
 End
End
!********************************************
Barrier ISC2
RRHO         !          1
 Geometry[angstrom]         13
C    0.000000    0.000000    0.000000
 C    0.000000    0.000000    1.369166
 C    1.205080    0.000000    2.085849
 C    2.452223   -0.008658    1.412985
 C    2.504596    0.010806    0.056860
 C    1.272144    0.132142   -0.789303
 O    1.084948    1.429262   -1.243127
 H    3.457787    0.013513   -0.461058
 H   -0.920232    0.012282   -0.569953
 H   -0.941762   -0.022069    1.906297
 H    1.182857    0.002368    3.169603
 H    3.366176   -0.028653    1.996048
 H    1.287603   -0.584872   -1.628140
```

Core RigidRotor
SymmetryFactor 0.5
End
Frequencies[1/cm] 32
3228.7 3212.0 3204.3 3191.6 3173.0 2945.0 1602.4 1541.7 1450.5 1410.2
1359.0 1245.3 1214.9 1188.5 1157.0 1061.8 1037.8 1009.3 1002.3 992.17
969.65 903.62 845.35 779.23 707.17 685.57 584.47 550.57 521.14 412.85
279.88 142.15
ZeroEnergy[kcal/mol] -9.2
ElectronicLevels[1/cm] 1
0.0000000000000000 3.0000000000000000
End
End
!***

SINGLET

Well W1[S]
Species
RRHO
Geometry[angstrom] 13
C 0.00000 0.00000 0.00000
C 0.00000 0.00000 1.39050
C 1.19604 0.00000 2.09618
C 2.39936 0.00012 1.39601
C 2.41301 0.00005 0.00908
C 1.20903 0.00002 -0.69035
O 1.27575 0.00021 -2.05011
H 3.34269 0.00019 -0.54700
H -0.93911 0.00014 -0.54601
H -0.94556 0.00006 1.92118
H 1.19236 0.00018 3.17947
H 3.33983 0.00022 1.93567
H 0.39096 -0.00141 -2.41844
Core RigidRotor
SymmetryFactor 1.0000000000000000
End
Rotor Hindered
Group 13
Axis 7 6
Symmetry 2
Potential[kcal/mol] 9
0.00 0.38 1.41 2.59 3.35 3.35 2.60 1.42 0.39
End
Frequencies[1/cm] 32
3920.0 3221.1 3213.9 3199.9 3191.2 3173.1 1683.7 1671.9 1546.6 1515.4

```
 1374.6    1354.3    1303.5    1207.5    1200.2    1181.6    1106.5    1056.2    1020.0    1007.7
 988.83    906.06    840.50    838.77    770.74    696.72    637.14    541.13    519.71    421.53
 415.29    236.89
 ZeroEnergy[kcal/mol]          0.
 ElectronicLevels[1/cm]              1
 0.0000000000000000      1.0000000000000000
End
End
!************************************
Well W2
Species
RRHO
Geometry[angstrom]          13
 C   0.00000  0.00000  0.00000
 C   0.00000  0.00000  1.34412
 C   1.22781  0.00000  2.13008
 C   2.42088 -0.00022  1.52851
 C   2.55322 -0.00064  0.04057
 C   1.25833  0.00022 -0.76002
 O   1.28141  0.00110 -1.97382
 H   3.13723 -0.86942 -0.28713
 H  -0.91731  0.00015 -0.57730
 H  -0.94508  0.00003  1.87872
 H   1.15108  0.00018  3.21113
 H   3.33466 -0.00025  2.11424
 H   3.13910  0.86661 -0.28775
        Core     RigidRotor
                 SymmetryFactor    1.0000000000000000
        End
   Frequencies[1/cm]        33
 46.189    267.69    442.04    463.14    498.41    537.19    583.27    731.27    755.03    814.51
 945.15    954.39    962.12    1008.8    1014.6    1033.4    1174.5    1203.3    1206.6    1259.1
 1355.3    1405.9    1411.3    1454.8    1651.8    1726.2    1787.7    3044.8    3076.4    3179.5
 3185.4    3209.7    3216.2
ZeroEnergy[kcal/mol] 17.85141539098150
 ElectronicLevels[1/cm]              1
 0.0000000000000000      1.0000000000000000
End
End
!************************************
Well W3
Species
RRHO
Geometry[angstrom]          13
 C   0.00000  0.00000  0.00000
 C   0.00000  0.00000  1.40315
 C   1.17237  0.00000  2.10236
 C   2.43044 -0.00006  1.36105
```

```
 C   2.38381  -0.00039  -0.09766
 C   1.17458   0.00115  -0.85655
 O   1.11080   0.00260  -2.09349
 H   3.32505  -0.00116  -0.63879
 H  -0.95357  -0.00044  -0.52166
 H  -0.95016  -0.00017   1.92687
 H   1.19682   0.00025   3.18521
 H   3.04248  -0.85554   1.70067
 H   3.04046   0.85759   1.69929
          Core     RigidRotor
                   SymmetryFactor    1.0000000000000000
          End
    Frequencies[1/cm]        33
  36.303     112.22     388.25     427.31     466.19     512.84     586.35     662.58     731.28     806.66
  860.87     948.70     965.77     971.13     1011.4     1037.6     1092.9     1125.3     1182.3     1283.1
  1299.1     1335.6     1409.9     1426.5     1512.3     1593.4     1691.3     2984.1     3000.3     3180.7
  3196.4     3198.7     3221.2
ZeroEnergy[kcal/mol] 57.12394355501510
 ElectronicLevels[1/cm]              1
  0.0000000000000000       1.0000000000000000
 End
 End
 !************************************
Well W4
 Species
 RRHO
 Geometry[angstrom]          13
 C   0.00000   0.00000   0.00000
 C   0.00000   0.00000   1.49488
 C   1.22695   0.00000   2.01102
 C   2.31393  -0.02515   0.96403
 C   1.54661   0.06933  -0.35354
 C   0.63554   1.19658  -0.58135
 O   0.48426   2.28799  -1.03628
 H   1.93180  -0.43576  -1.23340
 H  -0.71847  -0.59195  -0.55781
 H  -0.91942   0.02952   2.06597
 H   1.44609   0.03056   3.07135
 H   2.90687  -0.94457   1.02065
 H   3.00996   0.81212   1.07842
          Core     RigidRotor
                   SymmetryFactor    0.5000000000000000
          End
    Frequencies[1/cm]        33
  147.42     214.22     396.93     519.92     548.35     673.76     696.01     765.36     775.12     881.88
  911.52     950.51     963.40     996.28     1013.9     1038.7     1072.8     1092.8     1136.1     1192.8
  1250.8     1288.1     1334.9     1372.2     1492.2     1691.7     1959.7     3045.5     3084.2     3170.9
  3178.2     3208.2     3232.5
```

```
ZeroEnergy[kcal/mol] 50.52521766281842
  ElectronicLevels[1/cm]                1
   0.0000000000000000       1.0000000000000000
 End
 End
 !************************************
Well W5
 Species
 RRHO
 Geometry[angstrom]           13
  C   0.00000   0.00000   0.00000
  C   0.00000   0.00000   1.33480
  C   1.20785   0.00000   2.14530
  C   1.26694   0.00065   3.48708
  C   0.12864   0.00187   4.39630
  C   0.26882   0.00112   5.70630
  O   0.38496   0.00114   6.85629
  H   0.92753   0.00008  -0.56468
  H  -0.92356  -0.00010  -0.56651
  H  -0.95732  -0.00016   1.85029
  H   2.14922  -0.00060   1.60316
  H   2.24717   0.00052   3.95324
  H  -0.89626   0.00269   4.04589
          Core     RigidRotor
                   SymmetryFactor    1.0000000000000000
          End
 Rotor                       Hindered
 Group    4   5   6   7  11  12  13
 Axis          3          2
 Symmetry          1
 Potential[kcal/mol]         18
   0.00    0.57    2.10    3.88    4.75    4.11    2.83    2.25    2.93    4.08    2.93    2.25    2.83    4.10    4.75
3.89    2.11    0.58
 End
 Rotor                       Hindered
 Group    6   7  13
 Axis          5          4
 Symmetry          1
 Potential[kcal/mol]         18
   0.00    0.53    1.98    3.71    4.72    4.16    2.75    1.71    1.50    1.62    1.50    1.70    2.74    4.15    4.72
3.72    1.99    0.54
 End
    Frequencies[1/cm]         31
   3244.7     3214.1     3190.9     3170.4     3163.2     3148.9     2243.5     1730.7     1676.9     1493.6
   1422.4     1360.3     1332.2     1293.9     1193.4     1148.9     1043.1     1032.1     988.38     948.59
   927.46     810.60     694.68     687.32     606.15     604.58     535.87     370.41     351.06     223.85
   117.18
ZeroEnergy[kcal/mol] 41.93025540519802
```

```
 ElectronicLevels[1/cm]                1
  0.0000000000000000       1.0000000000000000
 End
 End
!***********************************
Well W6
 Species
 RRHO
 Geometry[angstrom]            13
 C   0.00000   0.00000   0.00000
 C   0.00000   0.00000   1.33445
 C   1.25260   0.00000   2.14600
 C   2.50800   0.00013   1.33880
 C   2.51260   0.00008   0.00436
 C   1.25765   0.00006  -0.77837
 O   1.25971   0.00024  -1.99479
 H   3.43371   0.00014  -0.56778
 H  -0.91912   0.00003  -0.57533
 H  -0.93959  -0.00005   1.87973
 H   1.25149  -0.86841   2.81903
 H   3.44570   0.00021   1.88732
 H   1.25139   0.86833   2.81913
       Core    RigidRotor
            SymmetryFactor    2.0000000000000000
       End
    Frequencies[1/cm]         33
  116.80     301.40     362.02     461.32     508.59     575.44     581.19     756.16     777.33     849.14
  894.13     946.72     967.94     1004.8     1025.8     1034.9     1153.3     1202.4     1217.4     1282.1
  1390.5     1421.2     1434.4     1437.5     1694.8     1728.4     1787.2     3023.9     3046.1     3175.0
  3175.8     3209.0     3210.8
ZeroEnergy[kcal/mol] 16.39124864604130
 ElectronicLevels[1/cm]                1
  0.0000000000000000       1.0000000000000000
 End
 End
!***********************************
Well W7
Species
 RRHO
 Geometry[angstrom]            13
  C   0.00000   0.00000   0.00000
  C   0.00000   0.00000   1.46772
  C   1.15732   0.00000   2.15213
  C   2.43064  -0.19724   1.48602
  C   2.50528  -0.38794   0.15716
  C   1.32186  -0.20463  -0.69147
  O   0.68440   1.06425  -0.64609
  H   3.43316  -0.69727  -0.31092
```

```
 H   -0.91086  -0.30218  -0.50999
 H   -0.95344  -0.01804   1.98386
 H    1.14593   0.03955   3.23588
 H    3.32002  -0.29728   2.09855
 H    1.33161  -0.64931  -1.68305
           Core     RigidRotor
                    SymmetryFactor    1.0000000000000000
           End
   Frequencies[1/cm]        33
  254.08    316.25    467.70    546.78    629.73    636.90    717.35    793.12    799.13    871.37
  948.97    981.66    992.89    1003.6    1010.4    1029.8    1073.5    1149.7    1202.0    1214.6
  1281.8    1367.6    1408.9    1433.8    1475.0    1639.9    1716.6    3146.0    3154.9    3183.0
  3191.5    3205.0    3212.9
 ZeroEnergy[kcal/mol]          44.8
 ElectronicLevels[1/cm]              1
  0.0000000000000000      1.0000000000000000
 End
 End
!**********************************
Bimolecular Pr1
 Fragment PROD1
 RRHO
 Geometry[angstrom]          11
 C    0.00000   0.00000   0.00000
 C    0.00000   0.00000   1.34273
 C    1.41616   0.00000   1.83662
 C    2.21914   0.00009   0.56995
 C    1.38515   0.00011  -0.48230
 H   -0.86689  -0.00019   1.98945
 H    1.63232  -0.87888   2.45727
 H    1.63256   0.87836   2.45788
 H    3.30023   0.00028   0.53829
 H    1.67337   0.00015  -1.52576
 H   -0.87398  -0.00016  -0.63876
       Core    RigidRotor
           SymmetryFactor    2.0000000000000000
       End
   Frequencies[1/cm]        27
  342.40    527.09    684.49    727.06    819.63    822.35    921.13    939.48    970.67    975.48
  981.71    1022.8    1117.4    1134.5    1136.5    1277.8    1329.4    1406.6    1423.1    1583.6
  1665.5    3041.6    3076.0    3210.9    3220.6    3238.9    3246.1
 ZeroEnergy[kcal/mol]          0.
 ElectronicLevels[1/cm]              1
  0.0000000000000000      1.0000000000000000
 End

!**********************************
 Fragment PROD2
```

```
 RRHO
 Geometry[angstrom]            2
  C   0.00000  0.00000  0.00000
  O   0.00000  0.00000  1.12558
       Core    RigidRotor
            SymmetryFactor   1.0000000000000000
       End
    Frequencies[1/cm]          1
   2246.3
  ZeroEnergy[kcal/mol]           0.
  ElectronicLevels[1/cm]                1
   0.0000000000000000       1.0000000000000000
 End
GroundEnergy[kcal/mol] 25.81989553537770
 End
 !************************************
Bimolecular Pr2
 Fragment PROD1
 RRHO
 Geometry[angstrom]           12
  C   0.00000  0.00000  0.00000
  C   0.00000  0.00000  1.37119
  C   1.21377  0.00000  2.07978
  C   2.44348 -0.00010  1.39922
  C   2.47485 -0.00044  0.02840
  C   1.24610 -0.00012 -0.74281
  O   1.26029  0.00010 -1.98690
  H   3.40600 -0.00091 -0.52626
  H  -0.91819  0.00004 -0.57585
  H  -0.93656 -0.00008  1.91743
  H   1.20134 -0.00003  3.16375
  H   3.36728 -0.00027  1.96676
       Core    RigidRotor
            SymmetryFactor   2.0000000000000000
       End
    Frequencies[1/cm]         30
   185.39     379.18     452.70     481.88     533.57     600.08     648.42     796.58     807.60     811.15
   930.53     987.40     1000.5     1003.5     1019.2     1100.3     1167.7     1172.8     1285.8     1340.3
   1438.0     1452.2     1520.1     1567.0     1609.6     3190.3     3197.7     3210.1     3218.2     3221.9
  ZeroEnergy[kcal/mol]           0.
  ElectronicLevels[1/cm]                1
   0.0000000000000000       2.0000000000000000

End
 !************************************
 Fragment PROD2
  Atom
  Name H
```

```
 ElectronicLevels[1/cm]                1
  0.0000000000000000       2.0000000000000000

 End
GroundEnergy[kcal/mol] 86.20041595547007
 End
!***********************************
Bimolecular Pr3
 Fragment PROD1
 RRHO
 Geometry[angstrom]          10
 C    0.00000   0.00000   0.00000
 C    0.00000   0.00000   1.40503
 C    1.18349   0.00000   2.15937
 C    2.45707  -0.00007   1.56606
 C    2.34118   0.00000   0.19066
 C    1.29573  -0.00019  -0.47578
 H   -0.91103  -0.00017  -0.58384
 H   -0.95238  -0.00002   1.92514
 H    1.11420  -0.00002   3.24229
 H    3.37101  -0.00014   2.14533
        Core    RigidRotor
             SymmetryFactor    2.0000000000000000
        End
    Frequencies[1/cm]         24
  395.51     401.45     455.46     608.83     626.35     759.81     847.02     884.68     946.28     993.80
  1011.6     1081.3     1122.0     1171.6     1276.1     1320.5     1440.8     1498.5     1500.3     2053.7
  3186.1     3201.9     3224.0     3227.6
 ZeroEnergy[kcal/mol]           0.
 ElectronicLevels[1/cm]                1
  0.0000000000000000       1.0000000000000000
 End

!***********************************
 Fragment PROD2
 RRHO
 Geometry[angstrom]           3
 H    0.00000   0.00000   0.00000
 O    0.00000   0.00000   0.95757
 H    0.92404   0.00000   1.20875
        Core    RigidRotor
             SymmetryFactor    2.0000000000000000
        End
    Frequencies[1/cm]          3
  1609.7     3898.2     4007.6
 ZeroEnergy[kcal/mol]           0.
 ElectronicLevels[1/cm]                1
  0.0000000000000000       1.0000000000000000
```

End

GroundEnergy[kcal/mol] 72.77797703373600
End
!**********************************
Barrier TS1 W1^S W2
Variational
RRHO ! 1
Geometry[angstrom] 13
C -1.29536 -0.09090 -0.35037
C -1.29592 -0.09201 1.02570
C -0.10969 -0.09099 1.81028
C 1.11775 -0.18739 1.20806
C 1.18547 -0.21631 -0.21141
C -0.02739 0.02506 -0.95264
O 0.28218 0.53933 -2.09659
H 1.86936 -0.93002 -0.67661
H -2.20677 -0.02563 -0.93156
H -2.25020 -0.06336 1.54245
H -0.19383 -0.06920 2.88973
H 2.01732 -0.30379 1.80333
H 1.34789 0.60228 -1.47268
Core RigidRotor
SymmetryFactor 0.50000000000000000
End
Frequencies[1/cm] 32

3221.4	3216.0	3192.9	3184.6	3126.6	1940.6	1663.8	1575.3	1566.2	1472.1
1408.6	1349.7	1272.8	1189.8	1177.1	1104.1	1074.4	1015.9	1010.9	1002.7
969.69	881.68	833.63	771.54	764.32	670.93	635.10	545.62	515.67	452.12
392.67	167.98								

ZeroEnergy[kcal/mol] 62.832594881441420
ElectronicLevels[1/cm] 1
0.0000000000000000 1.0000000000000000
End
!**
RRHO ! 2
Geometry[angstrom] 13
C -1.29554 -0.09098 -0.35021
C -1.29604 -0.09196 1.02545
C -0.10940 -0.09105 1.81028
C 1.11769 -0.18726 1.20831
C 1.18585 -0.21598 -0.21202
C -0.02728 0.02528 -0.95342
O 0.28146 0.53919 -2.09655
H 1.86777 -0.93256 -0.67578
H -2.20686 -0.02587 -0.93152
H -2.25023 -0.06327 1.54241
H -0.19374 -0.06941 2.88971

H 2.01732 -0.30351 1.80348
H 1.35591 0.59998 -1.45960
Core RigidRotor
SymmetryFactor 0.50000000000000000
End
Frequencies[1/cm] 32

3221.6	3216.2	3193.2	3184.6	3125.9	1924.5	1663.4	1574.3	1565.3	1471.5
1408.5	1352.3	1273.3	1189.9	1177.2	1104.2	1074.3	1016.0	1011.3	1001.9
970.31	881.40	833.57	770.96	764.24	666.53	633.97	543.09	516.53	453.05
391.01	174.89								

ZeroEnergy[kcal/mol] 63.899200810011781
ElectronicLevels[1/cm] 1
0.0000000000000000 1.0000000000000000
End
!**
RRHO ! 3
Geometry[angstrom] 13
C -1.29573 -0.09106 -0.35005
C -1.29615 -0.09191 1.02520
C -0.10912 -0.09111 1.81028
C 1.11763 -0.18713 1.20857
C 1.18624 -0.21565 -0.21263
C -0.02717 0.02550 -0.95421
O 0.28074 0.53904 -2.09652
H 1.86619 -0.93509 -0.67495
H -2.20695 -0.02611 -0.93148
H -2.25025 -0.06319 1.54237
H -0.19365 -0.06963 2.88970
H 2.01732 -0.30323 1.80362
H 1.36394 0.59771 -1.44652
Core RigidRotor
SymmetryFactor 0.50000000000000000
End
Frequencies[1/cm] 32

3221.8	3216.3	3193.4	3184.6	3125.2	1910.4	1663.0	1573.4	1564.5	1470.9
1408.5	1355.3	1273.7	1190.0	1177.2	1104.5	1074.4	1016.3	1011.6	1001.2
970.97	881.17	833.65	770.77	764.10	662.46	632.67	540.56	517.44	454.05
389.03	181.50								

ZeroEnergy[kcal/mol] 64.863890604193469
ElectronicLevels[1/cm] 1
0.0000000000000000 1.0000000000000000
End
!**
RRHO ! 4
Geometry[angstrom] 13
C -1.29591 -0.09113 -0.34988
C -1.29627 -0.09186 1.02495
C -0.10883 -0.09116 1.81028

C   1.11757 -0.18700   1.20883
C   1.18663 -0.21532 -0.21324
C -0.02706   0.02573 -0.95500
O   0.28001   0.53890 -2.09648
H   1.86462 -0.93760 -0.67410
H -2.20704 -0.02635 -0.93144
H -2.25027 -0.06311   1.54233
H -0.19356 -0.06984   2.88968
H   2.01732 -0.30296   1.80377
H   1.37195   0.59545 -1.43342
Core RigidRotor
SymmetryFactor 0.50000000000000000
End
Frequencies[1/cm] 32

3222.0	3216.5	3193.6	3184.6	3124.3	1898.3	1662.7	1572.7	1563.7	1470.4
1408.4	1358.6	1274.0	1190.2	1177.3	1105.0	1074.9	1016.8	1011.7	1000.5
971.65	880.96	833.86	770.90	763.89	658.67	631.18	537.97	518.41	455.09
386.71	187.73								

ZeroEnergy[kcal/mol] 65.732524782786950
ElectronicLevels[1/cm] 1
0.0000000000000000 1.0000000000000000
End
!***
RRHO ! 5
Geometry[angstrom] 13
C -1.29609 -0.09119 -0.34972
C -1.29639 -0.09182   1.02470
C -0.10854 -0.09122   1.81028
C   1.11751 -0.18687   1.20909
C   1.18702 -0.21500 -0.21386
C -0.02695   0.02595 -0.95579
O   0.27929   0.53875 -2.09645
H   1.86306 -0.94010 -0.67325
H -2.20712 -0.02658 -0.93139
H -2.25030 -0.06303   1.54229
H -0.19346 -0.07006   2.88967
H   2.01732 -0.30269   1.80391
H   1.37994   0.59321 -1.42031
Core RigidRotor
SymmetryFactor 0.50000000000000000
End
Frequencies[1/cm] 32

3222.2	3216.6	3193.8	3184.6	3123.4	1888.0	1662.4	1572.0	1562.9	1470.0
1408.4	1362.3	1274.2	1190.3	1177.5	1106.0	1075.8	1017.3	1011.8	999.80
972.33	880.77	834.18	771.29	763.57	655.06	629.48	535.24	519.38	456.16
384.02	193.51								

ZeroEnergy[kcal/mol] 66.491709709257787
ElectronicLevels[1/cm] 1

```
 0.0000000000000000       1.0000000000000000
End
!********************************************
RRHO          !           6
Geometry[angstrom]         13
C   -1.29627  -0.09126  -0.34955
C   -1.29652  -0.09177   1.02446
C   -0.10826  -0.09127   1.81028
C    1.11745  -0.18674   1.20935
C    1.18742  -0.21468  -0.21447
C   -0.02685   0.02618  -0.95659
O    0.27856   0.53861  -2.09641
H    1.86150  -0.94259  -0.67238
H   -2.20721  -0.02681  -0.93135
H   -2.25032  -0.06296   1.54225
H   -0.19337  -0.07027   2.88965
H    2.01732  -0.30242   1.80406
H    1.38791   0.59099  -1.40718
        Core      RigidRotor
      SymmetryFactor   0.50000000000000000
End
   Frequencies[1/cm]         32
  3222.4     3216.8     3194.1     3184.6     3122.4     1879.7     1662.1     1571.5     1562.1     1469.8
  1408.4     1366.2     1274.4     1190.5     1177.7     1107.4     1077.1     1017.9     1011.8     999.18
  973.03     880.61     834.61     771.94     763.14     651.68     627.57     532.52     520.32     457.24
  380.97     198.76
ZeroEnergy[kcal/mol]    67.142131578040232
 ElectronicLevels[1/cm]                1
 0.0000000000000000       1.0000000000000000
End
!********************************************
RRHO          !           7
Geometry[angstrom]         13
C   -1.29645  -0.09132  -0.34938
C   -1.29664  -0.09173   1.02421
C   -0.10797  -0.09133   1.81027
C    1.11739  -0.18661   1.20961
C    1.18782  -0.21436  -0.21508
C   -0.02674   0.02640  -0.95738
O    0.27784   0.53846  -2.09638
H    1.85996  -0.94508  -0.67151
H   -2.20729  -0.02704  -0.93131
H   -2.25035  -0.06290   1.54221
H   -0.19328  -0.07049   2.88964
H    2.01732  -0.30215   1.80420
H    1.39585   0.58879  -1.39404
        Core      RigidRotor
      SymmetryFactor   0.50000000000000000
```

End
Frequencies[1/cm] 32
3222.7 3216.9 3194.3 3184.6 3121.3 1873.5 1662.0 1571.1 1561.6 1469.5
1408.5 1370.3 1274.6 1190.6 1178.0 1109.5 1078.8 1018.5 1011.8 998.62
973.74 880.48 835.17 772.87 762.61 648.67 625.42 529.99 521.22 458.28
377.53 203.39
ZeroEnergy[kcal/mol] 67.684762497916165
ElectronicLevels[1/cm] 1
0.0000000000000000 1.0000000000000000
End
!***
RRHO ! 8
Geometry[angstrom] 13
C -1.29663 -0.09138 -0.34921
C -1.29677 -0.09168 1.02396
C -0.10768 -0.09138 1.81027
C 1.11732 -0.18648 1.20987
C 1.18822 -0.21404 -0.21570
C -0.02663 0.02662 -0.95818
O 0.27712 0.53831 -2.09635
H 1.85841 -0.94756 -0.67062
H -2.20737 -0.02727 -0.93127
H -2.25037 -0.06284 1.54218
H -0.19319 -0.07070 2.88962
H 2.01731 -0.30189 1.80435
H 1.40376 0.58660 -1.38088
Core RigidRotor
SymmetryFactor 0.50000000000000000
End
Frequencies[1/cm] 32
3222.9 3217.1 3194.5 3184.6 3120.1 1869.0 1662.0 1571.0 1561.4 1469.4
1408.7 1374.7 1274.7 1190.8 1178.3 1112.4 1080.7 1019.2 1011.7 998.13
974.45 880.37 835.88 773.95 762.00 646.07 623.01 527.67 522.03 459.26
373.70 207.32
ZeroEnergy[kcal/mol] 68.106623408155184
ElectronicLevels[1/cm] 1
0.0000000000000000 1.0000000000000000
End
!***
RRHO ! 9
Geometry[angstrom] 13
C -1.29680 -0.09144 -0.34904
C -1.29689 -0.09164 1.02371
C -0.10739 -0.09144 1.81026
C 1.11726 -0.18634 1.21013
C 1.18863 -0.21373 -0.21632
C -0.02652 0.02685 -0.95899
O 0.27639 0.53816 -2.09631

H 1.85686 -0.95003 -0.66972
H -2.20745 -0.02749 -0.93123
H -2.25040 -0.06278 1.54214
H -0.19310 -0.07092 2.88961
H 2.01731 -0.30162 1.80449
H 1.41162 0.58443 -1.36769
Core RigidRotor
SymmetryFactor 0.50000000000000000
End
Frequencies[1/cm] 32
3223.1 3217.2 3194.7 3184.7 3118.8 1866.1 1662.2 1571.1 1561.3 1469.3
1408.8 1379.3 1274.9 1191.0 1178.8 1116.3 1082.6 1019.9 1011.6 997.70
975.15 880.27 836.65 775.07 761.33 643.79 620.34 525.99 522.07 460.14
369.54 210.44
ZeroEnergy[kcal/mol] 68.412259621558729
ElectronicLevels[1/cm] 1
0.0000000000000000 1.0000000000000000
End
!***
RRHO ! 10
Geometry[angstrom] 13
C -1.29698 -0.09150 -0.34886
C -1.29703 -0.09159 1.02346
C -0.10711 -0.09149 1.81025
C 1.11720 -0.18621 1.21040
C 1.18904 -0.21342 -0.21693
C -0.02641 0.02708 -0.95982
O 0.27567 0.53800 -2.09626
H 1.85530 -0.95248 -0.66881
H -2.20753 -0.02772 -0.93119
H -2.25040 -0.06273 1.54209
H -0.19300 -0.07114 2.88954
H 2.01733 -0.30136 1.80465
H 1.41943 0.58227 -1.35450
Core RigidRotor
SymmetryFactor 0.50000000000000000
End
Frequencies[1/cm] 32
3223.4 3217.6 3194.8 3184.9 3117.7 1865.1 1662.4 1571.4 1561.3 1469.3
1409.0 1383.9 1275.0 1191.2 1179.5 1121.4 1084.4 1020.5 1011.5 997.33
975.81 880.20 837.45 776.26 760.50 641.76 617.45 526.05 520.33 460.87
365.07 212.62
ZeroEnergy[kcal/mol] 68.596110829391404
ElectronicLevels[1/cm] 1
0.0000000000000000 1.0000000000000000
End
!***
RRHO ! 11

```
Geometry[angstrom]        13
 C   0.00000  0.00000  0.00000
 C   0.00000  0.00000  1.37189
 C   1.19035  0.00000  2.15892
 C   2.41428 -0.09452  1.55935
 C   2.48660 -0.12156  0.13116
 C   1.27088  0.11886 -0.61189
 O   1.57212  0.62938 -1.74747
 H   3.15091 -0.86344 -0.31917
 H  -0.91045  0.06361 -0.58246
 H  -0.95329  0.02886  1.89074
 H   1.10425  0.02019  3.23826
 H   3.31444 -0.20954  2.15347
 H   2.72437  0.67170 -0.99249
         Core     RigidRotor
       SymmetryFactor   0.50000000000000000
End
   Frequencies[1/cm]        32
  3223.6     3217.7     3195.0     3184.9     3116.2     1866.0     1662.8     1571.8     1561.7     1469.4
  1409.4     1388.6     1275.1     1191.4     1180.5     1127.5     1085.9     1021.2     1011.4     997.00
  976.50     880.20     838.38     777.64     759.73     640.07     614.55     526.87     518.39     461.39
  360.45     213.88
ZeroEnergy[kcal/mol]    68.672142496882753
 ElectronicLevels[1/cm]               1
  0.0000000000000000       1.0000000000000000
End
!*******************************************
RRHO          !          12
Geometry[angstrom]        13
C  -1.29734 -0.09160 -0.34851
C  -1.29729 -0.09151  1.02297
C  -0.10653 -0.09161  1.81024
C   1.11708 -0.18594  1.21092
C   1.18987 -0.21280 -0.21818
C  -0.02619  0.02754 -0.96145
O   0.27422  0.53769 -2.09619
H   1.85220 -0.95745 -0.66695
H  -2.20769 -0.02816 -0.93110
H  -2.25045 -0.06265  1.54203
H  -0.19282 -0.07158  2.88952
H   2.01732 -0.30083  1.80495
H   1.43494  0.57804 -1.32801
         Core     RigidRotor
       SymmetryFactor   0.50000000000000000
End
   Frequencies[1/cm]        32
  3223.8     3217.8     3195.3     3184.9     3114.6     1868.5     1663.3     1572.5     1562.6     1469.6
  1409.9     1393.3     1275.3     1191.8     1181.9     1134.4     1087.3     1021.8     1011.2     996.72
```

977.19 880.24 839.43 779.08 758.92 638.66 611.65 527.81 516.79 461.66
355.76 214.09
ZeroEnergy[kcal/mol] 68.633407630711485
ElectronicLevels[1/cm] 1
0.0000000000000000 1.0000000000000000
End
!***
RRHO ! 13
Geometry[angstrom] 13
C -1.29752 -0.09165 -0.34834
C -1.29743 -0.09146 1.02272
C -0.10623 -0.09166 1.81023
C 1.11702 -0.18580 1.21118
C 1.19030 -0.21250 -0.21880
C -0.02609 0.02776 -0.96225
O 0.27350 0.53753 -2.09616
H 1.85066 -0.95998 -0.66601
H -2.20778 -0.02838 -0.93106
H -2.25050 -0.06261 1.54201
H -0.19272 -0.07181 2.88955
H 2.01730 -0.30056 1.80508
H 1.44264 0.57599 -1.31472
Core RigidRotor
SymmetryFactor 0.50000000000000000
End
Frequencies[1/cm] 32
3224.0 3217.6 3195.6 3184.7 3112.6 1872.1 1664.0 1573.3 1563.6 1469.8
1410.4 1397.9 1275.4 1192.2 1183.9 1141.8 1088.4 1022.5 1011.0 996.47
977.86 880.32 840.49 780.45 758.17 637.48 608.77 528.81 515.26 461.63
351.15 213.17
ZeroEnergy[kcal/mol] 68.476618215880035
ElectronicLevels[1/cm] 1
0.0000000000000000 1.0000000000000000
End
!***
RRHO ! 14
Geometry[angstrom] 13
C -1.29770 -0.09170 -0.34816
C -1.29757 -0.09142 1.02247
C -0.10594 -0.09172 1.81022
C 1.11696 -0.18567 1.21145
C 1.19073 -0.21220 -0.21942
C -0.02598 0.02798 -0.96308
O 0.27278 0.53738 -2.09612
H 1.84909 -0.96250 -0.66504
H -2.20786 -0.02860 -0.93101
H -2.25053 -0.06259 1.54197
H -0.19263 -0.07203 2.88954

```
H   2.01730  -0.30029   1.80523
H   1.45028   0.57395  -1.30142
       Core     RigidRotor
     SymmetryFactor   0.50000000000000000
End
  Frequencies[1/cm]        32
 3224.3     3217.7     3195.7     3184.7     3110.8     1877.4     1664.7     1574.3     1564.8     1470.0
 1411.0     1402.4     1275.5     1192.9     1186.6     1148.9     1089.2     1023.1     1010.9     996.24
 978.46     880.44     841.50     781.76     757.31     636.42     605.91     529.81     513.88     461.25
 346.61     210.96
ZeroEnergy[kcal/mol]    68.215039227466474
 ElectronicLevels[1/cm]              1
 0.0000000000000000       1.0000000000000000
End
!*******************************************
RRHO          !          15
Geometry[angstrom]         13
C  -1.29788  -0.09174  -0.34798
C  -1.29771  -0.09138   1.02223
C  -0.10565  -0.09177   1.81021
C   1.11690  -0.18553   1.21172
C   1.19116  -0.21190  -0.22004
C  -0.02588   0.02821  -0.96391
O   0.27206   0.53721  -2.09608
H   1.84752  -0.96503  -0.66407
H  -2.20794  -0.02882  -0.93097
H  -2.25056  -0.06256   1.54194
H  -0.19253  -0.07226   2.88953
H   2.01729  -0.30002   1.80539
H   1.45787   0.57194  -1.28811
       Core     RigidRotor
     SymmetryFactor   0.50000000000000000
End
  Frequencies[1/cm]        32
 3224.5     3217.8     3195.9     3184.6     3108.9     1885.0     1665.6     1575.4     1566.3     1470.4
 1412.0     1406.4     1275.7     1195.1     1189.5     1155.2     1089.9     1023.7     1010.7     996.05
 979.04     880.66     842.57     783.15     756.45     635.48     603.19     530.77     513.14     460.56
 342.31     207.46
ZeroEnergy[kcal/mol]    67.851744244707544
 ElectronicLevels[1/cm]              1
 0.0000000000000000       1.0000000000000000
End
!*******************************************
RRHO          !          16
Geometry[angstrom]         13
C  -1.29806  -0.09178  -0.34780
C  -1.29785  -0.09133   1.02198
C  -0.10535  -0.09183   1.81020
```

C 1.11685 -0.18539 1.21198
C 1.19160 -0.21160 -0.22067
C -0.02577 0.02845 -0.96474
O 0.27134 0.53705 -2.09604
H 1.84594 -0.96757 -0.66307
H -2.20803 -0.02903 -0.93092
H -2.25059 -0.06254 1.54191
H -0.19243 -0.07249 2.88952
H 2.01729 -0.29976 1.80554
H 1.46541 0.56997 -1.27477
Core RigidRotor
SymmetryFactor 0.50000000000000000
End
Frequencies[1/cm] 32

3224.7	3217.8	3196.1	3184.6	3107.0	1894.5	1666.6	1576.8	1568.4	1470.8
1414.0	1409.3	1275.9	1200.6	1191.0	1160.2	1090.4	1024.2	1010.5	995.88
979.59	880.98	843.69	784.56	755.60	634.65	600.60	531.66	512.98	459.62
338.33	202.67								

ZeroEnergy[kcal/mol] 67.386047073169021
ElectronicLevels[1/cm] 1
0.0000000000000000 1.0000000000000000
End
!***
RRHO ! 17
Geometry[angstrom] 13
C -1.29824 -0.09182 -0.34762
C -1.29799 -0.09129 1.02173
C -0.10505 -0.09188 1.81019
C 1.11679 -0.18525 1.21225
C 1.19205 -0.21131 -0.22129
C -0.02567 0.02868 -0.96559
O 0.27062 0.53689 -2.09600
H 1.84434 -0.97013 -0.66207
H -2.20811 -0.02925 -0.93088
H -2.25061 -0.06253 1.54188
H -0.19234 -0.07272 2.88951
H 2.01729 -0.29948 1.80570
H 1.47292 0.56804 -1.26142
Core RigidRotor
SymmetryFactor 0.50000000000000000
End
Frequencies[1/cm] 32

3224.9	3217.9	3196.2	3184.6	3105.0	1905.3	1667.7	1578.5	1570.4	1471.3
1417.2	1410.9	1276.0	1208.7	1191.7	1163.7	1090.8	1024.7	1010.3	995.73
980.08	881.34	844.73	785.85	754.74	633.91	598.15	532.47	513.01	458.41
334.72	196.56								

ZeroEnergy[kcal/mol] 66.832713012928306
ElectronicLevels[1/cm] 1

0.0000000000000000 1.0000000000000000
End
!***
RRHO ! 18
Geometry[angstrom] 13
C -1.29841 -0.09186 -0.34743
C -1.29814 -0.09125 1.02148
C -0.10476 -0.09194 1.81018
C 1.11673 -0.18511 1.21252
C 1.19250 -0.21102 -0.22191
C -0.02556 0.02891 -0.96644
O 0.26989 0.53672 -2.09596
H 1.84273 -0.97271 -0.66104
H -2.20820 -0.02947 -0.93083
H -2.25065 -0.06252 1.54185
H -0.19224 -0.07295 2.88949
H 2.01729 -0.29921 1.80586
H 1.48038 0.56616 -1.24806
Core RigidRotor
SymmetryFactor 0.50000000000000000
End
Frequencies[1/cm] 32
3225.1 3217.9 3196.4 3184.6 3102.9 1917.8 1668.8 1580.5 1572.2 1471.9
1420.7 1411.9 1276.2 1218.4 1192.0 1166.2 1091.1 1025.2 1010.1 995.61
980.49 881.79 845.70 787.09 753.89 633.25 595.82 533.20 513.22 456.97
331.49 189.18
ZeroEnergy[kcal/mol] 66.186825062532034
ElectronicLevels[1/cm] 1
0.0000000000000000 1.0000000000000000
End
!***
RRHO ! 19
Geometry[angstrom] 13
C -1.29859 -0.09189 -0.34725
C -1.29829 -0.09121 1.02123
C -0.10446 -0.09200 1.81017
C 1.11667 -0.18497 1.21280
C 1.19296 -0.21074 -0.22253
C -0.02545 0.02915 -0.96730
O 0.26917 0.53655 -2.09591
H 1.84111 -0.97530 -0.66001
H -2.20829 -0.02968 -0.93078
H -2.25068 -0.06252 1.54183
H -0.19214 -0.07318 2.88948
H 2.01729 -0.29893 1.80602
H 1.48781 0.56432 -1.23469
Core RigidRotor
SymmetryFactor 0.50000000000000000

```
End
   Frequencies[1/cm]         32
  3225.2     3217.9     3196.5     3184.6     3100.8     1932.9     1670.1     1583.0     1574.1     1472.6
  1424.2     1412.7     1276.6     1229.2     1192.3     1168.0     1091.3     1025.6     1009.9     995.53
  980.85     882.37     846.65     788.33     753.07     632.66     593.66     533.90     513.74     455.41
  328.69     180.70
ZeroEnergy[kcal/mol]    65.464035581860810
 ElectronicLevels[1/cm]              1
  0.0000000000000000       1.0000000000000000
End
!*******************************************
RRHO          !          20
Geometry[angstrom]         13
C  -1.29877  -0.09192  -0.34706
C  -1.29844  -0.09117   1.02098
C  -0.10416  -0.09206   1.81015
C   1.11661  -0.18482   1.21307
C   1.19343  -0.21046  -0.22315
C  -0.02535   0.02938  -0.96816
O   0.26845   0.53638  -2.09586
H   1.83946  -0.97793  -0.65895
H  -2.20838  -0.02990  -0.93073
H  -2.25071  -0.06252   1.54180
H  -0.19204  -0.07342   2.88947
H   2.01728  -0.29865   1.80618
H   1.49520   0.56254  -1.22131
          Core      RigidRotor
        SymmetryFactor   0.50000000000000000
End
   Frequencies[1/cm]         32
  3225.4     3217.9     3196.6     3184.6     3098.7     1949.8     1671.4     1585.8     1575.7     1473.5
  1427.5     1413.5     1277.2     1240.0     1192.5     1169.2     1091.5     1026.0     1009.7     995.49
  981.14     883.04     847.52     789.54     752.26     632.14     591.65     534.52     514.46     453.80
  326.23     171.16
ZeroEnergy[kcal/mol]    64.660456135787069
 ElectronicLevels[1/cm]              1
  0.0000000000000000       1.0000000000000000
End
!*******************************************
RRHO          !          21
Geometry[angstrom]         13
C  -1.29895  -0.09195  -0.34688
C  -1.29859  -0.09113   1.02072
C  -0.10385  -0.09211   1.81014
C   1.11655  -0.18468   1.21334
C   1.19390  -0.21019  -0.22377
C  -0.02525   0.02962  -0.96904
O   0.26772   0.53621  -2.09581
```

```
H   1.83780  -0.98057  -0.65788
H  -2.20847  -0.03011  -0.93067
H  -2.25074  -0.06252   1.54177
H  -0.19193  -0.07365   2.88945
H   2.01728  -0.29837   1.80635
H   1.50257   0.56081  -1.20792
        Core     RigidRotor
      SymmetryFactor   0.50000000000000000
End
   Frequencies[1/cm]         32
  3225.5     3217.9     3196.7     3184.6     3096.4     1968.2     1672.8     1589.0     1577.0     1474.4
  1430.4     1414.2     1278.1     1250.5     1192.8     1170.2     1091.5     1026.4     1009.5     995.49
  981.35     883.81     848.28     790.69     751.47     631.67     589.77     535.08     515.24     452.18
  324.03     160.48
ZeroEnergy[kcal/mol]    63.794411658016035
 ElectronicLevels[1/cm]                 1
  0.0000000000000000       1.0000000000000000
End
!********************************************
    Tunneling    Eckart
    ImaginaryFrequency[1/cm]    2226.9398000000001
    WellDepth[kcal/mol]         68.67
    WellDepth[kcal/mol]         50.82
End
End
Barrier TS2 W2 W3
 Variational
 RRHO          !          1
 Geometry[angstrom]         13
 C  -1.21352  -0.00853  -0.24556
 C  -1.21051  -0.00948   1.13759
 C  -0.03438  -0.00875   1.88142
 C   1.18998   0.02608   1.17695
 C   1.20273  -0.02287  -0.27650
 C  -0.02116  -0.00916  -1.06527
 O  -0.01042  -0.03381  -2.29882
 H   2.15561  -0.01253  -0.79481
 H  -2.15691  -0.00632  -0.78116
 H  -2.16221  -0.01235   1.65948
 H  -0.03993  -0.01102   2.96257
 H   2.12579  -0.15471   1.69698
 H   1.27723   1.12295   0.68045
        Core     RigidRotor
      SymmetryFactor   0.50000000000000000
End
   Frequencies[1/cm]         32
  3236.2     3212.9     3206.1     3185.4     3183.1     2355.0     1703.0     1583.8     1560.4     1450.9
  1412.7     1350.8     1275.1     1210.3     1185.5     1161.0     1069.2     1025.3     1013.4     985.74
```

975.31 879.14 816.47 806.88 740.20 659.62 600.29 523.21 466.71 441.90
398.76 143.15
ZeroEnergy[kcal/mol] 60.343667635022470
ElectronicLevels[1/cm] 1
0.0000000000000000 1.0000000000000000
End
!***
RRHO ! 2
Geometry[angstrom] 13
C -1.21324 -0.00863 -0.24561
C -1.21049 -0.00948 1.13800
C -0.03495 -0.00882 1.88145
C 1.19045 0.02737 1.17624
C 1.20238 -0.02400 -0.27705
C -0.02087 -0.00925 -1.06530
O -0.01052 -0.03378 -2.29905
H 2.15522 -0.01177 -0.79547
H -2.15671 -0.00611 -0.78115
H -2.16231 -0.01232 1.65966
H -0.04023 -0.01085 2.96263
H 2.12536 -0.15780 1.69656
H 1.27828 1.12561 0.69540
Core RigidRotor
SymmetryFactor 0.50000000000000000
End
Frequencies[1/cm] 32
3236.1 3212.7 3205.9 3185.0 3182.2 2378.7 1702.2 1582.3 1560.3 1450.4
1412.8 1351.5 1275.5 1213.0 1185.4 1161.8 1069.3 1024.2 1013.1 985.13
971.19 878.41 816.75 807.80 737.55 657.68 600.00 523.06 466.06 441.46
397.30 141.61
ZeroEnergy[kcal/mol] 60.551104600556286
ElectronicLevels[1/cm] 1
0.0000000000000000 1.0000000000000000
End
!***
RRHO ! 3
Geometry[angstrom] 13
C -1.21295 -0.00873 -0.24567
C -1.21048 -0.00949 1.13841
C -0.03552 -0.00889 1.88149
C 1.19092 0.02867 1.17554
C 1.20203 -0.02512 -0.27759
C -0.02057 -0.00935 -1.06532
O -0.01063 -0.03374 -2.29927
H 2.15481 -0.01107 -0.79614
H -2.15650 -0.00591 -0.78115
H -2.16241 -0.01229 1.65983
H -0.04053 -0.01065 2.96269

```
H   2.12491  -0.16096   1.69612
H   1.27936   1.12831   0.71035
        Core     RigidRotor
      SymmetryFactor   0.50000000000000000
End
   Frequencies[1/cm]         32
 3236.0    3212.4    3205.7    3184.9    3181.0    2402.7    1701.4    1580.9    1560.2    1449.8
 1413.0    1352.2    1276.0    1216.0    1185.3    1162.3    1069.4    1023.3    1012.8    984.85
 967.00    877.56    817.03    808.74    734.69    655.68    599.72    522.90    465.42    441.00
 395.77    140.03
ZeroEnergy[kcal/mol]    60.733769989171889
 ElectronicLevels[1/cm]                1
 0.0000000000000000       1.0000000000000000
End
!*******************************************
RRHO          !          4
Geometry[angstrom]         13
C   -1.21267  -0.00883  -0.24572
C   -1.21048  -0.00949   1.13882
C   -0.03609  -0.00897   1.88152
C    1.19138   0.02996   1.17484
C    1.20168  -0.02622  -0.27813
C   -0.02026  -0.00946  -1.06536
O   -0.01075  -0.03371  -2.29950
H    2.15440  -0.01044  -0.79682
H   -2.15630  -0.00571  -0.78114
H   -2.16251  -0.01226   1.66001
H   -0.04083  -0.01043   2.96275
H    2.12443  -0.16419   1.69568
H    1.28047   1.13105   0.72530
        Core     RigidRotor
      SymmetryFactor   0.50000000000000000
End
   Frequencies[1/cm]         32
 3235.8    3212.1    3205.5    3184.8    3179.8    2426.8    1700.7    1579.5    1560.0    1449.3
 1413.2    1353.0    1276.5    1219.2    1185.2    1162.8    1069.5    1022.4    1012.5    984.71
 963.02    876.62    817.31    809.71    731.65    653.63    599.44    522.73    464.81    440.54
 394.22    138.44
ZeroEnergy[kcal/mol]    60.891949715216881
 ElectronicLevels[1/cm]                1
 0.0000000000000000       1.0000000000000000
End
!*******************************************
RRHO          !          5
Geometry[angstrom]         13
C   -1.21238  -0.00893  -0.24576
C   -1.21048  -0.00950   1.13924
C   -0.03666  -0.00904   1.88155
```

C 1.19185 0.03125 1.17414
C 1.20134 -0.02731 -0.27866
C -0.01994 -0.00958 -1.06540
O -0.01087 -0.03367 -2.29973
H 2.15397 -0.00988 -0.79751
H -2.15609 -0.00551 -0.78114
H -2.16263 -0.01223 1.66018
H -0.04113 -0.01017 2.96281
H 2.12393 -0.16748 1.69522
H 1.28162 1.13382 0.74023
Core RigidRotor
SymmetryFactor 0.50000000000000000
End
Frequencies[1/cm] 32

3235.7	3211.8	3205.3	3184.7	3178.5	2451.0	1700.0	1578.1	1559.9	1448.7
1413.4	1353.9	1277.0	1222.6	1185.1	1163.0	1069.5	1021.6	1012.1	984.64
959.37	875.59	817.58	810.71	728.43	651.51	599.17	522.55	464.22	440.08
392.63	136.84								

ZeroEnergy[kcal/mol] 61.031404227470034
ElectronicLevels[1/cm] 1
0.0000000000000000 1.0000000000000000
End
!***
RRHO ! 6
Geometry[angstrom] 13
C -1.21209 -0.00904 -0.24581
C -1.21049 -0.00951 1.13965
C -0.03722 -0.00912 1.88158
C 1.19231 0.03255 1.17344
C 1.20100 -0.02838 -0.27918
C -0.01961 -0.00970 -1.06545
O -0.01099 -0.03363 -2.29995
H 2.15354 -0.00938 -0.79822
H -2.15587 -0.00530 -0.78114
H -2.16275 -0.01219 1.66036
H -0.04143 -0.00989 2.96287
H 2.12340 -0.17084 1.69474
H 1.28280 1.13661 0.75516
Core RigidRotor
SymmetryFactor 0.50000000000000000
End
Frequencies[1/cm] 32

3235.5	3211.5	3205.1	3184.7	3177.1	2474.6	1699.3	1576.9	1559.7	1448.1
1413.5	1354.9	1277.5	1226.0	1185.0	1163.2	1069.5	1020.9	1011.6	984.61
956.09	874.46	817.85	811.73	725.01	649.34	598.89	522.35	463.66	439.62
391.02	135.21								

ZeroEnergy[kcal/mol] 61.145215124044796
ElectronicLevels[1/cm] 1

```
 0.0000000000000000       1.0000000000000000
End
!*******************************************
RRHO          !          7
Geometry[angstrom]         13
C   -1.21180  -0.00914  -0.24585
C   -1.21050  -0.00952   1.14006
C   -0.03778  -0.00920   1.88160
C    1.19277   0.03385   1.17275
C    1.20066  -0.02942  -0.27970
C   -0.01928  -0.00983  -1.06550
O   -0.01113  -0.03359  -2.30018
H    2.15311  -0.00895  -0.79893
H   -2.15566  -0.00509  -0.78113
H   -2.16288  -0.01215   1.66053
H   -0.04173  -0.00959   2.96292
H    2.12285  -0.17427   1.69425
H    1.28402   1.13940   0.77009
          Core      RigidRotor
      SymmetryFactor   0.50000000000000000
End
   Frequencies[1/cm]         32
 3235.3     3211.2     3204.9     3184.7     3175.7     2497.4     1698.6     1575.7     1559.5     1447.5
 1413.7     1355.9     1278.0     1229.6     1184.9     1163.3     1069.5     1020.3     1011.1     984.61
 953.19     873.25     818.10     812.76     721.40     647.10     598.62     522.15     463.13     439.15
 389.39     133.52
ZeroEnergy[kcal/mol]    61.239071375133022
 ElectronicLevels[1/cm]                 1
 0.0000000000000000       1.0000000000000000
End
!*******************************************
RRHO          !          8
Geometry[angstrom]         13
C   -1.21151  -0.00925  -0.24588
C   -1.21052  -0.00953   1.14047
C   -0.03833  -0.00929   1.88162
C    1.19323   0.03515   1.17206
C    1.20032  -0.03045  -0.28021
C   -0.01893  -0.00998  -1.06557
O   -0.01127  -0.03356  -2.30041
H    2.15266  -0.00860  -0.79964
H   -2.15544  -0.00488  -0.78112
H   -2.16301  -0.01211   1.66071
H   -0.04203  -0.00926   2.96298
H    2.12227  -0.17776   1.69373
H    1.28528   1.14220   0.78501
          Core      RigidRotor
      SymmetryFactor   0.50000000000000000
```

```
End
   Frequencies[1/cm]        32
 3235.1     3210.8     3204.7     3184.7     3174.2     2519.4     1698.0     1574.6     1559.1     1446.8
 1413.9     1357.0     1278.6     1233.1     1184.8     1163.3     1069.6     1019.7     1010.5     984.62
 950.69     872.00     818.35     813.80     717.64     644.79     598.35     521.93     462.63     438.68
 387.73     131.80
ZeroEnergy[kcal/mol]    61.306683590412838
 ElectronicLevels[1/cm]              1
 0.0000000000000000       1.0000000000000000
End
!********************************************
RRHO          !          9
Geometry[angstrom]        13
C  -1.21122  -0.00936  -0.24592
C  -1.21055  -0.00954   1.14089
C  -0.03888  -0.00937   1.88164
C   1.19368   0.03645   1.17137
C   1.19999  -0.03146  -0.28071
C  -0.01857  -0.01013  -1.06563
O  -0.01141  -0.03351  -2.30064
H   2.15221  -0.00831  -0.80037
H  -2.15522  -0.00467  -0.78112
H  -2.16315  -0.01207   1.66088
H  -0.04234  -0.00890   2.96303
H   2.12166  -0.18132   1.69320
H   1.28657   1.14498   0.79992
          Core     RigidRotor
       SymmetryFactor   0.50000000000000000
End
   Frequencies[1/cm]        32
 3234.9     3210.5     3204.4     3184.6     3172.7     2540.6     1697.5     1573.6     1558.7     1446.1
 1414.1     1358.1     1279.2     1236.6     1184.7     1163.2     1069.6     1019.3     1009.8     984.63
 948.61     870.74     818.60     814.86     713.77     642.41     598.07     521.71     462.17     438.21
 386.06     130.08
ZeroEnergy[kcal/mol]    61.361073717766750
 ElectronicLevels[1/cm]              1
 0.0000000000000000       1.0000000000000000
End
!********************************************
RRHO          !          10
Geometry[angstrom]        13
C  -1.21092  -0.00945  -0.24596
C  -1.21057  -0.00955   1.14130
C  -0.03943  -0.00948   1.88165
C   1.19411   0.03777   1.17067
C   1.19967  -0.03245  -0.28120
C  -0.01821  -0.01030  -1.06577
O  -0.01157  -0.03347  -2.30080
```

H 2.15179 -0.00808 -0.80108
H -2.15504 -0.00443 -0.78112
H -2.16333 -0.01202 1.66107
H -0.04263 -0.00852 2.96311
H 2.12097 -0.18495 1.69260
H 1.28792 1.14778 0.81482
Core RigidRotor
SymmetryFactor 0.50000000000000000
End
Frequencies[1/cm] 32

3234.4	3210.1	3203.9	3184.3	3171.4	2561.0	1697.5	1572.8	1558.3	1445.5
1414.4	1359.2	1279.8	1240.1	1184.6	1163.0	1069.6	1019.1	1009.0	984.66
946.98	869.57	818.80	815.98	709.92	639.97	597.79	521.49	461.73	437.65
384.39	128.23								

ZeroEnergy[kcal/mol] 61.396181098351078
ElectronicLevels[1/cm] 1
0.0000000000000000 1.0000000000000000
End
!***
RRHO ! 11
Geometry[angstrom] 13
C 0.00000 0.00000 0.00000
C 0.00000 0.00000 1.38767
C 1.17064 0.00000 2.12761
C 2.40516 0.04865 1.41595
C 2.40995 -0.02384 -0.03573
C 1.19277 -0.00091 -0.81990
O 1.19887 -0.02386 -2.05505
H 3.36191 0.00160 -0.55586
H -0.94415 0.00534 -0.53511
H -0.95283 -0.00241 1.90717
H 1.16768 0.00148 3.20907
H 3.33090 -0.17915 1.93798
H 2.49992 1.16004 1.07568
Core RigidRotor
SymmetryFactor 0.50000000000000000
End
Frequencies[1/cm] 32

3234.2	3209.8	3203.7	3184.2	3169.9	2581.2	1697.0	1572.2	1557.6	1444.8
1414.6	1360.4	1280.4	1243.6	1184.5	1162.8	1069.6	1018.8	1008.2	984.68
945.62	868.38	819.01	817.06	705.92	637.41	597.51	521.25	461.34	437.18
382.72	126.51								

ZeroEnergy[kcal/mol] 61.412134393622117
ElectronicLevels[1/cm] 1
0.0000000000000000 1.0000000000000000
End
!***
RRHO ! 12

```
Geometry[angstrom]        13
C  -1.21032  -0.00968  -0.24601
C  -1.21067  -0.00958   1.14213
C  -0.04051  -0.00966   1.88168
C   1.19501   0.04040   1.16932
C   1.19903  -0.03437  -0.28217
C  -0.01745  -0.01065  -1.06595
O  -0.01189  -0.03338  -2.30127
H   2.15087  -0.00787  -0.80256
H  -2.15457  -0.00402  -0.78111
H  -2.16365  -0.01193   1.66142
H  -0.04324  -0.00765   2.96321
H   2.11959  -0.19247   1.69143
H   1.29073   1.15322   0.84462
        Core     RigidRotor
      SymmetryFactor   0.50000000000000000
End
  Frequencies[1/cm]        32
 3233.9    3209.5    3203.4    3184.2    3168.1    2600.3    1696.6    1571.7    1556.9    1444.0
 1414.8    1361.5    1281.0    1247.1    1184.4    1162.6    1069.5    1018.7    1007.2    984.71
 944.58    867.28    819.22    818.18    701.94    634.75    597.22    521.01    461.04    436.71
 381.09    124.84
ZeroEnergy[kcal/mol]    61.414651165206599
 ElectronicLevels[1/cm]              1
 0.0000000000000000       1.0000000000000000
End
!*******************************************
RRHO          !          13
Geometry[angstrom]        13
C  -1.21002  -0.00982  -0.24602
C  -1.21074  -0.00961   1.14254
C  -0.04105  -0.00972   1.88169
C   1.19548   0.04172   1.16867
C   1.19872  -0.03531  -0.28264
C  -0.01707  -0.01083  -1.06599
O  -0.01207  -0.03333  -2.30158
H   2.15038  -0.00789  -0.80333
H  -2.15429  -0.00385  -0.78110
H  -2.16379  -0.01190   1.66158
H  -0.04354  -0.00716   2.96323
H   2.11890  -0.19636   1.69085
H   1.29220   1.15585   0.85949
        Core     RigidRotor
      SymmetryFactor   0.50000000000000000
End
  Frequencies[1/cm]        32
 3234.0    3209.2    3203.6    3184.6    3166.2    2618.7    1695.6    1571.3    1555.9    1443.3
 1414.9    1362.7    1281.8    1250.5    1184.3    1162.3    1069.5    1018.5    1006.2    984.73
```

943.72 866.23 819.49 819.19 697.87 631.98 596.93 520.75 460.81 436.32
379.47 123.31
ZeroEnergy[kcal/mol] 61.397399135630514
ElectronicLevels[1/cm] 1
0.0000000000000000 1.0000000000000000
End
!***
RRHO ! 14
Geometry[angstrom] 13
C -1.20971 -0.00994 -0.24603
C -1.21080 -0.00963 1.14296
C -0.04158 -0.00981 1.88170
C 1.19592 0.04304 1.16800
C 1.19841 -0.03622 -0.28309
C -0.01668 -0.01103 -1.06610
O -0.01225 -0.03327 -2.30183
H 2.14992 -0.00798 -0.80408
H -2.15404 -0.00366 -0.78109
H -2.16398 -0.01185 1.66176
H -0.04384 -0.00664 2.96327
H 2.11812 -0.20032 1.69021
H 1.29374 1.15846 0.87435
Core RigidRotor
SymmetryFactor 0.50000000000000000
End
Frequencies[1/cm] 32
3233.7 3208.9 3203.3 3184.5 3164.4 2635.9 1695.2 1571.0 1555.0 1442.5
1415.1 1363.9 1282.4 1253.7 1184.2 1161.9 1069.5 1018.4 1005.1 984.75
943.16 865.34 820.40 819.55 693.95 629.09 596.63 520.49 460.60 435.84
377.87 121.63
ZeroEnergy[kcal/mol] 61.372513918298743
ElectronicLevels[1/cm] 1
0.0000000000000000 1.0000000000000000
End
!***
RRHO ! 15
Geometry[angstrom] 13
C -1.20940 -0.01007 -0.24604
C -1.21088 -0.00966 1.14338
C -0.04211 -0.00990 1.88170
C 1.19636 0.04438 1.16735
C 1.19811 -0.03710 -0.28354
C -0.01627 -0.01124 -1.06622
O -0.01245 -0.03322 -2.30208
H 2.14945 -0.00814 -0.80484
H -2.15379 -0.00347 -0.78108
H -2.16417 -0.01180 1.66193
H -0.04414 -0.00610 2.96331

H 2.11731 -0.20438 1.68953
H 1.29533 1.16101 0.88921
Core RigidRotor
SymmetryFactor 0.50000000000000000
End
Frequencies[1/cm] 32

3233.5	3208.6	3203.1	3184.5	3162.7	2652.2	1694.8	1570.9	1553.9	1441.7
1415.3	1365.0	1283.2	1256.8	1184.1	1161.6	1069.5	1018.4	1004.0	984.77
942.81	864.58	821.50	819.70	690.13	626.08	596.30	520.23	460.45	435.36
376.33	119.96								

ZeroEnergy[kcal/mol] 61.334077811541419
ElectronicLevels[1/cm] 1
0.0000000000000000 1.0000000000000000
End
!***
RRHO ! 16
Geometry[angstrom] 13
C -1.20908 -0.01020 -0.24605
C -1.21097 -0.00968 1.14380
C -0.04263 -0.00999 1.88170
C 1.19679 0.04572 1.16669
C 1.19782 -0.03796 -0.28399
C -0.01586 -0.01146 -1.06634
O -0.01265 -0.03316 -2.30232
H 2.14899 -0.00839 -0.80559
H -2.15353 -0.00328 -0.78108
H -2.16437 -0.01175 1.66210
H -0.04443 -0.00553 2.96335
H 2.11645 -0.20852 1.68883
H 1.29698 1.16350 0.90405
Core RigidRotor
SymmetryFactor 0.50000000000000000
End
Frequencies[1/cm] 32

3233.2	3208.3	3202.9	3184.5	3160.9	2667.4	1694.5	1570.9	1552.8	1441.0
1415.6	1366.1	1283.9	1259.7	1184.0	1161.2	1069.5	1018.5	1002.9	984.78
942.62	863.96	822.60	819.83	686.44	622.94	595.96	519.97	460.39	434.86
374.86	118.30								

ZeroEnergy[kcal/mol] 61.282119406793242
ElectronicLevels[1/cm] 1
0.0000000000000000 1.0000000000000000
End
!***
RRHO ! 17
Geometry[angstrom] 13
C -1.20876 -0.01033 -0.24605
C -1.21106 -0.00971 1.14421
C -0.04315 -0.01008 1.88169

C 1.19722 0.04707 1.16605
C 1.19754 -0.03880 -0.28442
C -0.01544 -0.01169 -1.06648
O -0.01286 -0.03311 -2.30257
H 2.14853 -0.00870 -0.80635
H -2.15326 -0.00309 -0.78108
H -2.16458 -0.01170 1.66228
H -0.04472 -0.00492 2.96338
H 2.11555 -0.21275 1.68809
H 1.29870 1.16592 0.91887
Core RigidRotor
SymmetryFactor 0.5000000000000000
End
Frequencies[1/cm] 32

3232.9	3208.0	3202.7	3184.4	3159.1	2681.8	1694.2	1571.0	1551.6	1440.2
1415.8	1367.2	1284.7	1262.5	1184.0	1160.8	1069.4	1018.5	1001.7	984.78
942.59	863.50	823.71	819.94	682.93	619.69	595.58	519.70	460.42	434.36
373.49	116.67								

ZeroEnergy[kcal/mol] 61.216996096988651
ElectronicLevels[1/cm] 1
0.0000000000000000 1.0000000000000000
End
!***
RRHO ! 18
Geometry[angstrom] 13
C -1.20844 -0.01047 -0.24605
C -1.21117 -0.00974 1.14463
C -0.04366 -0.01017 1.88169
C 1.19764 0.04843 1.16541
C 1.19726 -0.03961 -0.28484
C -0.01501 -0.01193 -1.06662
O -0.01308 -0.03305 -2.30283
H 2.14808 -0.00910 -0.80710
H -2.15299 -0.00291 -0.78108
H -2.16480 -0.01165 1.66245
H -0.04501 -0.00429 2.96341
H 2.11461 -0.21709 1.68733
H 1.30048 1.16825 0.93367
Core RigidRotor
SymmetryFactor 0.5000000000000000
End
Frequencies[1/cm] 32

3232.7	3207.7	3202.4	3184.4	3157.2	2695.6	1693.9	1571.1	1550.3	1439.4
1416.0	1368.3	1285.6	1265.2	1183.9	1160.4	1069.4	1018.6	1000.5	984.78
942.68	863.20	824.85	820.03	679.66	616.34	595.15	519.44	460.60	433.84
372.25	115.09								

ZeroEnergy[kcal/mol] 61.145497622557954
ElectronicLevels[1/cm] 1

```
 0.0000000000000000       1.0000000000000000
End
!*******************************************
RRHO          !          19
Geometry[angstrom]         13
C   -1.20811  -0.01061  -0.24604
C   -1.21128  -0.00978   1.14504
C   -0.04417  -0.01026   1.88167
C    1.19806   0.04979   1.16478
C    1.19699  -0.04039  -0.28526
C   -0.01457  -0.01218  -1.06677
O   -0.01332  -0.03299  -2.30308
H    2.14764  -0.00958  -0.80785
H   -2.15270  -0.00274  -0.78108
H   -2.16503  -0.01159   1.66262
H   -0.04529  -0.00362   2.96344
H    2.11362  -0.22152   1.68653
H    1.30235   1.17050   0.94845
        Core     RigidRotor
     SymmetryFactor   0.50000000000000000
End
  Frequencies[1/cm]         32
 3232.4     3207.4     3202.2     3184.4     3155.4     2708.7     1693.7     1571.3     1549.1     1438.6
 1416.2     1369.3     1286.5     1267.7     1183.8     1160.0     1069.4     1018.8     999.34     984.77
 942.88     863.07     826.01     820.12     676.67     612.95     594.63     519.19     460.95     433.32
 371.16     113.56
ZeroEnergy[kcal/mol]    61.067909897848700
 ElectronicLevels[1/cm]                1
 0.0000000000000000       1.0000000000000000
End
!*******************************************
RRHO          !          20
Geometry[angstrom]         13
C   -1.20777  -0.01075  -0.24602
C   -1.21141  -0.00981   1.14546
C   -0.04468  -0.01035   1.88165
C    1.19847   0.05116   1.16416
C    1.19674  -0.04114  -0.28567
C   -0.01413  -0.01245  -1.06693
O   -0.01356  -0.03292  -2.30334
H    2.14720  -0.01015  -0.80860
H   -2.15241  -0.00257  -0.78108
H   -2.16528  -0.01154   1.66278
H   -0.04557  -0.00293   2.96346
H    2.11258  -0.22607   1.68569
H    1.30429   1.17265   0.96320
        Core     RigidRotor
     SymmetryFactor   0.50000000000000000
```

```
End
   Frequencies[1/cm]         32
  3232.1     3207.2     3202.0     3184.3     3153.5     2721.2     1693.5     1571.6     1547.8     1437.8
  1416.4     1370.2     1287.5     1270.0     1183.8     1159.6     1069.3     1018.9     998.14     984.76
  943.17     863.11     827.20     820.18     673.96     609.60     593.97     518.93     461.51     432.78
  370.27     112.06
ZeroEnergy[kcal/mol]    60.984175739991393
 ElectronicLevels[1/cm]                1
  0.0000000000000000       1.0000000000000000
End
!*******************************************
RRHO          !          21
Geometry[angstrom]         13
C   -1.20743  -0.01090  -0.24601
C   -1.21155  -0.00985   1.14587
C   -0.04518  -0.01044   1.88163
C    1.19888   0.05254   1.16355
C    1.19649  -0.04186  -0.28606
C   -0.01368  -0.01272  -1.06710
O   -0.01382  -0.03286  -2.30361
H    2.14677  -0.01080  -0.80935
H   -2.15210  -0.00240  -0.78109
H   -2.16554  -0.01148   1.66295
H   -0.04585  -0.00219   2.96347
H    2.11149  -0.23073   1.68482
H    1.30633   1.17471   0.97791
         Core      RigidRotor
        SymmetryFactor   0.50000000000000000
End
   Frequencies[1/cm]         32
  3231.8     3206.9     3201.8     3184.3     3151.7     2733.0     1693.3     1571.9     1546.5     1437.1
  1416.5     1371.1     1288.6     1272.0     1183.7     1159.1     1069.3     1019.1     996.95     984.74
  943.53     863.32     828.41     820.23     671.54     606.42     593.07     518.69     462.32     432.23
  369.62     110.59
ZeroEnergy[kcal/mol]    60.900727496481699
 ElectronicLevels[1/cm]                1
  0.0000000000000000       1.0000000000000000
End
!*******************************************
     Tunneling    Eckart
     ImaginaryFrequency[1/cm]    768.00018999999998
     WellDepth[kcal/mol]         43.56
     WellDepth[kcal/mol]          4.29
End
End
Barrier TS3 W3 W4
 Variational
 RRHO          !          1
```

```
Geometry[angstrom]        13
C  -1.09003  -0.24489  -0.25265
C  -1.11039  -0.21746   1.19007
C   0.04653  -0.21245   1.86490
C   1.27052  -0.38957   1.04147
C   0.99148  -0.34337  -0.41602
C  -0.06673   0.39379  -1.03974
O  -0.12787   0.93642  -2.12722
H   1.65932  -0.87553  -1.08966
H  -1.93485  -0.66274  -0.79668
H  -2.07205  -0.22557   1.69236
H   0.11239  -0.11798   2.94131
H   1.78214  -1.32091   1.30883
H   1.98979   0.41390   1.27032
      Core     RigidRotor
    SymmetryFactor   0.50000000000000000
End
  Frequencies[1/cm]        32
 3226.2    3192.9    3173.4    3165.9    3067.7    3009.2    1804.4    1667.8    1433.3    1419.4
 1395.3    1363.0    1349.4    1250.7    1161.0    1153.0    1111.0    1033.9    1020.0    974.22
 954.36    922.61    884.88    849.11    746.56    715.99    601.13    566.12    456.68    425.18
 253.53    229.10
ZeroEnergy[kcal/mol]    61.4687972787131000
 ElectronicLevels[1/cm]              1
 0.0000000000000000       1.0000000000000000
End
!*******************************************
RRHO          !          2
Geometry[angstrom]        13
C  -1.09284  -0.24243  -0.25217
C  -1.11101  -0.21772   1.18986
C   0.04662  -0.21312   1.86424
C   1.27039  -0.38971   1.04110
C   0.99476  -0.34155  -0.41644
C  -0.06661   0.39160  -1.03928
O  -0.12794   0.93506  -2.12681
H   1.66650  -0.86917  -1.08974
H  -1.94053  -0.65490  -0.79579
H  -2.07199  -0.22764   1.69346
H   0.11268  -0.12015   2.94078
H   1.78223  -1.32097   1.30856
H   1.98964   0.41354   1.27153
      Core     RigidRotor
    SymmetryFactor   0.50000000000000000
End
  Frequencies[1/cm]        32
 3226.2    3192.8    3173.7    3166.2    3067.3    3008.2    1801.9    1666.1    1434.4    1417.5
 1395.7    1363.9    1349.5    1251.3    1161.0    1152.8    1110.3    1034.1    1019.8    974.52
```

954.40 922.96 883.52 847.82 745.82 715.49 600.91 565.70 456.41 424.94
253.31 229.93
ZeroEnergy[kcal/mol] 61.5005286940189988
ElectronicLevels[1/cm] 1
0.0000000000000000 1.0000000000000000
End
!***
RRHO ! 3
Geometry[angstrom] 13
C -1.09562 -0.23996 -0.25168
C -1.11164 -0.21797 1.18964
C 0.04671 -0.21381 1.86359
C 1.27026 -0.38984 1.04072
C 0.99803 -0.33973 -0.41686
C -0.06648 0.38941 -1.03883
O -0.12801 0.93369 -2.12641
H 1.67369 -0.86272 -1.08980
H -1.94616 -0.64700 -0.79488
H -2.07193 -0.22970 1.69456
H 0.11296 -0.12237 2.94024
H 1.78234 -1.32101 1.30828
H 1.98948 0.41318 1.27275
Core RigidRotor
SymmetryFactor 0.5000000000000000
End
Frequencies[1/cm] 32
3226.2 3192.7 3174.0 3166.6 3066.8 3007.1 1799.4 1664.5 1435.6 1415.5
1396.1 1364.9 1349.7 1251.9 1161.1 1152.5 1109.5 1034.3 1019.7 974.84
954.44 923.31 882.20 846.51 745.10 714.97 600.70 565.31 456.14 424.68
253.07 230.76
ZeroEnergy[kcal/mol] 61.5321600393032595
ElectronicLevels[1/cm] 1
0.0000000000000000 1.0000000000000000
End
!***
RRHO ! 4
Geometry[angstrom] 13
C -1.09837 -0.23749 -0.25118
C -1.11228 -0.21822 1.18942
C 0.04680 -0.21452 1.86293
C 1.27012 -0.38997 1.04034
C 1.00127 -0.33792 -0.41728
C -0.06635 0.38719 -1.03839
O -0.12808 0.93232 -2.12601
H 1.68088 -0.85619 -1.08984
H -1.95177 -0.63903 -0.79395
H -2.07187 -0.23172 1.69565
H 0.11324 -0.12462 2.93970

H 1.78246 -1.32105 1.30800
H 1.98933 0.41283 1.27397
Core RigidRotor
SymmetryFactor 0.50000000000000000
End
Frequencies[1/cm] 32

3226.2	3192.5	3174.3	3166.9	3066.3	3006.0	1797.0	1662.8	1436.7	1413.6
1396.5	1365.8	1349.8	1252.5	1161.2	1152.2	1108.8	1034.4	1019.5	975.18
954.49	923.67	880.94	845.18	744.41	714.45	600.50	564.92	455.87	424.39
252.82	231.61								

ZeroEnergy[kcal/mol] 61.5575019942656380
ElectronicLevels[1/cm] 1
0.0000000000000000 1.0000000000000000
End
!***
RRHO ! 5
Geometry[angstrom] 13
C -1.10111 -0.23500 -0.25067
C -1.11292 -0.21847 1.18921
C 0.04689 -0.21524 1.86227
C 1.26999 -0.39008 1.03995
C 1.00449 -0.33610 -0.41769
C -0.06622 0.38497 -1.03797
O -0.12815 0.93095 -2.12561
H 1.68808 -0.84957 -1.08984
H -1.95733 -0.63100 -0.79301
H -2.07182 -0.23372 1.69674
H 0.11352 -0.12692 2.93916
H 1.78258 -1.32107 1.30771
H 1.98917 0.41248 1.27519
Core RigidRotor
SymmetryFactor 0.50000000000000000
End
Frequencies[1/cm] 32

3226.2	3192.4	3174.6	3167.2	3065.9	3005.0	1794.5	1661.1	1437.8	1411.7
1396.8	1366.7	1349.9	1253.1	1161.3	1151.9	1108.0	1034.5	1019.2	975.53
954.52	924.04	879.73	843.84	743.76	713.90	600.29	564.56	455.61	424.08
252.54	232.47								

ZeroEnergy[kcal/mol] 61.5764544888844893
ElectronicLevels[1/cm] 1
0.0000000000000000 1.0000000000000000
End
!***
RRHO ! 6
Geometry[angstrom] 13
C -1.10381 -0.23252 -0.25015
C -1.11357 -0.21872 1.18899
C 0.04699 -0.21599 1.86160

C 1.26986 -0.39018 1.03956
C 1.00768 -0.33428 -0.41809
C -0.06609 0.38274 -1.03754
O -0.12822 0.92957 -2.12521
H 1.69527 -0.84287 -1.08983
H -1.96286 -0.62290 -0.79204
H -2.07177 -0.23569 1.69782
H 0.11381 -0.12925 2.93861
H 1.78272 -1.32108 1.30741
H 1.98901 0.41213 1.27643
Core RigidRotor
SymmetryFactor 0.50000000000000000
End
Frequencies[1/cm] 32

3226.2	3192.3	3174.9	3167.6	3065.4	3003.9	1792.1	1659.4	1439.0	1409.8
1397.1	1367.5	1350.0	1253.7	1161.4	1151.5	1107.3	1034.7	1018.9	975.90
954.56	924.41	878.58	842.47	743.11	713.34	600.08	564.21	455.35	423.73
252.22	233.33								

ZeroEnergy[kcal/mol] 61.5890175231598062
ElectronicLevels[1/cm] 1
0.0000000000000000 1.0000000000000000
End
!***
RRHO ! 7
Geometry[angstrom] 13
C -1.10649 -0.23002 -0.24963
C -1.11423 -0.21896 1.18878
C 0.04708 -0.21675 1.86094
C 1.26972 -0.39027 1.03917
C 1.01085 -0.33246 -0.41849
C -0.06595 0.38050 -1.03713
O -0.12830 0.92818 -2.12482
H 1.70247 -0.83608 -1.08979
H -1.96835 -0.61474 -0.79105
H -2.07173 -0.23763 1.69889
H 0.11409 -0.13164 2.93807
H 1.78286 -1.32108 1.30710
H 1.98884 0.41180 1.27767
Core RigidRotor
SymmetryFactor 0.50000000000000000
End
Frequencies[1/cm] 32

3226.1	3192.2	3175.3	3167.9	3064.9	3002.9	1789.7	1657.7	1440.1	1407.9
1397.3	1368.4	1350.1	1254.3	1161.6	1151.1	1106.5	1034.8	1018.6	976.29
954.58	924.79	877.49	841.09	742.48	712.76	599.86	563.87	455.08	423.36
251.88	234.20								

ZeroEnergy[kcal/mol] 61.5951196185046354
ElectronicLevels[1/cm] 1

0.0000000000000000   1.0000000000000000
End
!***
RRHO   !   8
Geometry[angstrom]   13
C   -1.10915 -0.22752 -0.24909
C   -1.11489 -0.21919   1.18857
C   0.04718 -0.21752   1.86028
C   1.26959 -0.39035   1.03877
C   1.01400 -0.33064 -0.41888
C   -0.06582   0.37824 -1.03673
O   -0.12837   0.92679 -2.12444
H   1.70965 -0.82922 -1.08972
H   -1.97380 -0.60651 -0.79005
H   -2.07169 -0.23955   1.69996
H   0.11437 -0.13407   2.93752
H   1.78302 -1.32106   1.30679
H   1.98868   0.41147   1.27891
Core   RigidRotor
SymmetryFactor   0.50000000000000000
End
Frequencies[1/cm]   32

3226.1	3192.0	3175.6	3168.3	3064.4	3001.8	1787.3	1656.0	1441.2	1406.1
1397.3	1369.2	1350.1	1254.9	1161.8	1150.7	1105.8	1034.9	1018.2	976.69
954.61	925.18	876.45	839.68	741.86	712.16	599.64	563.54	454.81	422.95
251.52	235.07								

ZeroEnergy[kcal/mol]   61.6011359395452329
ElectronicLevels[1/cm]   1
0.0000000000000000   1.0000000000000000
End
!***
RRHO   !   9
Geometry[angstrom]   13
C   -1.11178 -0.22501 -0.24855
C   -1.11556 -0.21943   1.18836
C   0.04727 -0.21832   1.85961
C   1.26946 -0.39041   1.03837
C   1.01712 -0.32882 -0.41927
C   -0.06568   0.37598 -1.03634
O   -0.12844   0.92539 -2.12406
H   1.71682 -0.82228 -1.08962
H   -1.97921 -0.59822 -0.78903
H   -2.07166 -0.24144   1.70103
H   0.11465 -0.13654   2.93698
H   1.78320 -1.32102   1.30648
H   1.98849   0.41116   1.28016
Core   RigidRotor
SymmetryFactor   0.50000000000000000

End
Frequencies[1/cm] 32
3226.1 3191.9 3176.0 3168.6 3063.8 3000.8 1784.9 1654.3 1442.3 1404.5
1397.2 1370.0 1350.2 1255.5 1162.1 1150.2 1105.1 1035.0 1017.8 977.11
954.63 925.57 875.45 838.27 741.24 711.55 599.41 563.22 454.54 422.51
251.15 235.95
ZeroEnergy[kcal/mol] 61.6007770959596738
ElectronicLevels[1/cm] 1
0.0000000000000000 1.0000000000000000
End
!***
RRHO ! 10
Geometry[angstrom] 13
C -1.11441 -0.22248 -0.24800
C -1.11629 -0.21973 1.18816
C 0.04728 -0.21910 1.85892
C 1.26933 -0.39038 1.03797
C 1.02024 -0.32709 -0.41967
C -0.06547 0.37373 -1.03602
O -0.12845 0.92400 -2.12361
H 1.72393 -0.81546 -1.08949
H -1.98460 -0.58988 -0.78804
H -2.07166 -0.24347 1.70209
H 0.11485 -0.13899 2.93643
H 1.78345 -1.32076 1.30627
H 1.98820 0.41104 1.28134
Core RigidRotor
SymmetryFactor 0.50000000000000000
End
Frequencies[1/cm] 32
3225.8 3191.9 3176.6 3168.7 3063.7 2999.9 1783.1 1652.7 1443.4 1403.3
1396.7 1370.7 1350.2 1255.9 1162.3 1149.7 1104.3 1035.1 1017.3 977.55
954.63 925.99 874.44 836.84 740.53 710.96 599.16 562.88 454.19 421.96
250.62 236.63
ZeroEnergy[kcal/mol] 61.5936571033786490
ElectronicLevels[1/cm] 1
0.0000000000000000 1.0000000000000000
End
!***
RRHO ! 11
Geometry[angstrom] 13
C 0.00000 0.00000 0.00000
C 0.00000 0.00000 1.43536
C 1.16435 0.00000 2.10565
C 2.38614 -0.17048 1.28499
C 2.14024 -0.10530 -0.17260
C 1.05162 0.59140 -0.78820
O 0.98842 1.14249 -1.87576

H 2.84803 -0.58833 -0.84191
H -0.87288 -0.36148 -0.53952
H -0.95467 -0.02531 1.95054
H 1.23210 0.07834 3.18325
H 2.90058 -1.10080 1.55335
H 3.10499 0.63066 1.53007
Core RigidRotor
SymmetryFactor 0.50000000000000000
End
Frequencies[1/cm] 32

3225.8	3191.8	3177.2	3169.1	3063.1	2998.9	1780.8	1651.0	1444.6	1402.4
1395.7	1371.5	1350.2	1256.5	1162.6	1149.2	1103.7	1035.1	1016.9	978.00
954.65	926.38	873.52	835.41	739.88	710.32	598.91	562.58	453.90	421.46
250.21	237.50								

ZeroEnergy[kcal/mol] 61.5812341249750643
ElectronicLevels[1/cm] 1
0.0000000000000000 1.0000000000000000
End
!***
RRHO ! 12
Geometry[angstrom] 13
C -1.11953 -0.21742 -0.24687
C -1.11766 -0.22017 1.18776
C 0.04747 -0.22080 1.85759
C 1.26907 -0.39048 1.03717
C 1.02636 -0.32342 -0.42041
C -0.06518 0.36919 -1.03527
O -0.12860 0.92115 -2.12289
H 1.73824 -0.80109 -1.08918
H -1.99516 -0.57301 -0.78591
H -2.07162 -0.24705 1.70419
H 0.11540 -0.14421 2.93534
H 1.78380 -1.32069 1.30557
H 1.98788 0.41036 1.28394
Core RigidRotor
SymmetryFactor 0.50000000000000000
End
Frequencies[1/cm] 32

3225.8	3191.7	3177.5	3169.3	3062.5	2997.9	1778.4	1649.3	1445.7	1402.0
1394.4	1372.3	1350.3	1257.1	1162.9	1148.8	1103.0	1035.2	1016.4	978.48
954.71	926.85	872.70	834.02	739.31	709.68	598.68	562.33	453.66	420.95
249.84	238.41								

ZeroEnergy[kcal/mol] 61.5685681193759633
ElectronicLevels[1/cm] 1
0.0000000000000000 1.0000000000000000
End
!***
RRHO ! 13

```
Geometry[angstrom]        13
C  -1.12203  -0.21487  -0.24628
C  -1.11827  -0.22030   1.18758
C   0.04766  -0.22173   1.85694
C   1.26896  -0.39060   1.03675
C   1.02933  -0.32152  -0.42077
C  -0.06512   0.36691  -1.03486
O  -0.12877   0.91972  -2.12260
H   1.74534  -0.79364  -1.08904
H  -2.00033  -0.56447  -0.78475
H  -2.07155  -0.24859   1.70526
H   0.11581  -0.14696   2.93478
H   1.78395  -1.32084   1.30506
H   1.98785   0.40983   1.28530
         Core     RigidRotor
       SymmetryFactor   0.50000000000000000
End
   Frequencies[1/cm]        32
  3226.0    3191.6    3177.8    3169.9    3061.5    2996.8    1775.6    1647.5    1446.9    1402.0
  1392.7    1373.2    1350.4    1257.8    1163.2    1148.3    1102.4    1035.3    1015.9    978.96
  954.80    927.28    871.97    832.67    738.82    708.99    598.45    562.13    453.49    420.51
  249.63    239.49
ZeroEnergy[kcal/mol]    61.5508135637149420
 ElectronicLevels[1/cm]              1
  0.0000000000000000       1.0000000000000000
End
!*******************************************
RRHO          !          14
Geometry[angstrom]        13
C  -1.12452  -0.21231  -0.24567
C  -1.11894  -0.22049   1.18742
C   0.04780  -0.22265   1.85627
C   1.26886  -0.39065   1.03631
C   1.03231  -0.31966  -0.42114
C  -0.06500   0.36464  -1.03451
O  -0.12890   0.91827  -2.12227
H   1.75239  -0.78624  -1.08886
H  -2.00549  -0.55588  -0.78359
H  -2.07150  -0.25024   1.70634
H   0.11615  -0.14973   2.93424
H   1.78413  -1.32085   1.30460
H   1.98775   0.40942   1.28661
         Core     RigidRotor
       SymmetryFactor   0.50000000000000000
End
   Frequencies[1/cm]        32
  3225.9    3191.6    3178.3    3170.2    3060.9    2995.8    1773.3    1645.8    1448.2    1402.1
  1390.9    1374.0    1350.4    1258.3    1163.5    1147.8    1101.9    1035.3    1015.5    979.48
```

954.89 927.74 871.21 831.34 738.26 708.34 598.21 561.93 453.27 419.97
249.34 240.37
ZeroEnergy[kcal/mol] 61.5270269406449328
ElectronicLevels[1/cm] 1
0.0000000000000000 1.0000000000000000
End
!**
RRHO ! 15
Geometry[angstrom] 13
C -1.12699 -0.20973 -0.24506
C -1.11961 -0.22068 1.18726
C 0.04792 -0.22359 1.85561
C 1.26876 -0.39068 1.03588
C 1.03527 -0.31781 -0.42151
C -0.06487 0.36237 -1.03418
O -0.12903 0.91681 -2.12194
H 1.75943 -0.77876 -1.08865
H -2.01060 -0.54722 -0.78243
H -2.07147 -0.25188 1.70741
H 0.11648 -0.15255 2.93369
H 1.78432 -1.32083 1.30415
H 1.98764 0.40904 1.28794
Core RigidRotor
SymmetryFactor 0.50000000000000000
End
Frequencies[1/cm] 32
3225.9 3191.5 3178.9 3170.5 3060.3 2994.8 1771.1 1644.2 1449.4 1402.2
1389.0 1375.0 1350.4 1258.9 1163.8 1147.4 1101.4 1035.4 1015.0 980.01
955.00 928.22 870.48 830.06 737.72 707.68 597.98 561.77 453.08 419.43
249.16 241.20
ZeroEnergy[kcal/mol] 61.5039122162917903
ElectronicLevels[1/cm] 1
0.0000000000000000 1.0000000000000000
End
!**
RRHO ! 16
Geometry[angstrom] 13
C -1.12942 -0.20714 -0.24444
C -1.12030 -0.22086 1.18711
C 0.04804 -0.22456 1.85495
C 1.26866 -0.39070 1.03546
C 1.03820 -0.31595 -0.42186
C -0.06474 0.36009 -1.03385
O -0.12915 0.91534 -2.12163
H 1.76645 -0.77120 -1.08841
H -2.01564 -0.53850 -0.78126
H -2.07145 -0.25347 1.70847
H 0.11681 -0.15543 2.93314

```
H   1.78453  -1.32079   1.30369
H   1.98751   0.40867   1.28929
         Core     RigidRotor
       SymmetryFactor   0.50000000000000000
End
   Frequencies[1/cm]         32
  3225.8     3191.4     3179.4     3170.8     3059.6     2993.9     1768.9     1642.6     1450.6     1402.4
  1387.1     1375.9     1350.4     1259.5     1164.1     1146.9     1100.9     1035.4     1014.6     980.55
  955.13     928.70     869.79     828.84     737.21     707.02     597.75     561.66     452.92     418.87
  249.15     241.89
ZeroEnergy[kcal/mol]    61.4750084517250838
 ElectronicLevels[1/cm]                 1
  0.0000000000000000       1.0000000000000000
End
!********************************************
RRHO          !          17
Geometry[angstrom]         13
C  -1.13182  -0.20454  -0.24382
C  -1.12099  -0.22104   1.18696
C   0.04815  -0.22556   1.85429
C   1.26855  -0.39070   1.03503
C   1.04110  -0.31409  -0.42221
C  -0.06460   0.35782  -1.03353
O  -0.12927   0.91385  -2.12132
H   1.77344  -0.76355  -1.08814
H  -2.02063  -0.52972  -0.78007
H  -2.07144  -0.25503   1.70952
H   0.11712  -0.15837   2.93260
H   1.78475  -1.32073   1.30323
H   1.98737   0.40830   1.29066
         Core     RigidRotor
       SymmetryFactor   0.50000000000000000
End
   Frequencies[1/cm]         32
  3225.8     3191.4     3180.0     3171.1     3059.0     2992.9     1766.7     1641.0     1451.9     1402.6
  1385.1     1376.8     1350.4     1260.0     1164.4     1146.4     1100.4     1035.3     1014.2     981.12
  955.28     929.18     869.12     827.68     736.71     706.36     597.51     561.60     452.79     418.30
  249.42     242.32
ZeroEnergy[kcal/mol]    61.4401869854884124
 ElectronicLevels[1/cm]                 1
  0.0000000000000000       1.0000000000000000
End
!********************************************
RRHO          !          18
Geometry[angstrom]         13
C  -1.13418  -0.20193  -0.24320
C  -1.12169  -0.22122   1.18681
C   0.04826  -0.22659   1.85363
```

C 1.26845 -0.39069 1.03461
C 1.04398 -0.31221 -0.42254
C -0.06445 0.35554 -1.03323
O -0.12939 0.91235 -2.12103
H 1.78041 -0.75581 -1.08783
H -2.02554 -0.52089 -0.77889
H -2.07145 -0.25655 1.71056
H 0.11743 -0.16138 2.93205
H 1.78497 -1.32067 1.30274
H 1.98723 0.40795 1.29205
Core RigidRotor
SymmetryFactor 0.50000000000000000
End
Frequencies[1/cm] 32

3225.8	3191.4	3180.6	3171.3	3058.3	2992.1	1764.5	1639.4	1453.2	1402.8
1383.2	1377.7	1350.4	1260.6	1164.7	1145.9	1100.0	1035.2	1013.8	981.69
955.43	929.67	868.49	826.56	736.23	705.69	597.28	561.59	452.68	417.71
250.01	242.41								

ZeroEnergy[kcal/mol] 61.4055656592950809
ElectronicLevels[1/cm] 1
0.0000000000000000 1.0000000000000000
End
!***
RRHO ! 19
Geometry[angstrom] 13
C -1.13651 -0.19931 -0.24257
C -1.12240 -0.22139 1.18667
C 0.04835 -0.22765 1.85297
C 1.26834 -0.39066 1.03419
C 1.04683 -0.31033 -0.42286
C -0.06430 0.35327 -1.03293
O -0.12951 0.91083 -2.12076
H 1.78734 -0.74799 -1.08748
H -2.03038 -0.51200 -0.77770
H -2.07147 -0.25802 1.71158
H 0.11773 -0.16446 2.93151
H 1.78520 -1.32060 1.30225
H 1.98708 0.40759 1.29347
Core RigidRotor
SymmetryFactor 0.50000000000000000
End
Frequencies[1/cm] 32

3225.7	3191.4	3181.2	3171.6	3057.6	2991.2	1762.4	1637.8	1454.4	1403.0
1381.3	1378.5	1350.3	1261.1	1165.0	1145.3	1099.6	1035.1	1013.4	982.28
955.58	930.14	867.89	825.46	735.74	705.01	597.04	561.60	452.57	417.10
250.92	242.18								

ZeroEnergy[kcal/mol] 61.3707013059062260
ElectronicLevels[1/cm] 1

0.0000000000000000 1.0000000000000000
End
!***
RRHO ! 20
Geometry[angstrom] 13
C -1.13879 -0.19667 -0.24194
C -1.12312 -0.22155 1.18654
C 0.04844 -0.22875 1.85232
C 1.26823 -0.39063 1.03378
C 1.04966 -0.30843 -0.42317
C -0.06414 0.35101 -1.03265
O -0.12963 0.90929 -2.12050
H 1.79425 -0.74008 -1.08710
H -2.03514 -0.50306 -0.77650
H -2.07151 -0.25944 1.71260
H 0.11802 -0.16761 2.93097
H 1.78543 -1.32052 1.30174
H 1.98691 0.40724 1.29492
Core RigidRotor
SymmetryFactor 0.50000000000000000
End
Frequencies[1/cm] 32
3225.6 3191.4 3181.8 3171.9 3056.9 2990.3 1760.3 1636.3 1455.6 1403.1
1380.2 1378.4 1350.3 1261.7 1165.3 1144.8 1099.2 1034.9 1012.9 982.88
955.71 930.61 867.33 824.38 735.26 704.32 596.78 561.63 452.46 416.47
252.05 241.67
ZeroEnergy[kcal/mol] 61.3292902392826730
ElectronicLevels[1/cm] 1
0.0000000000000000 1.0000000000000000
End
!***
RRHO ! 21
Geometry[angstrom] 13
C -1.14104 -0.19403 -0.24131
C -1.12385 -0.22171 1.18641
C 0.04851 -0.22987 1.85167
C 1.26812 -0.39058 1.03337
C 1.05246 -0.30653 -0.42346
C -0.06398 0.34875 -1.03238
O -0.12976 0.90774 -2.12025
H 1.80111 -0.73210 -1.08669
H -2.03983 -0.49407 -0.77530
H -2.07157 -0.26083 1.71359
H 0.11829 -0.17084 2.93043
H 1.78566 -1.32043 1.30123
H 1.98674 0.40688 1.29640
Core RigidRotor
SymmetryFactor 0.50000000000000000

```
 End
   Frequencies[1/cm]        32
  3225.6    3191.4    3182.3    3172.2    3056.2    2989.4    1758.2    1634.8    1456.7    1403.3
  1380.5    1376.8    1350.2    1262.2    1165.6    1144.3    1098.8    1034.7    1012.3    983.48
  955.82    931.06    866.80    823.30    734.78    703.61    596.51    561.66    452.33    415.80
  253.34    240.97
 ZeroEnergy[kcal/mol]   61.2812037979680210
  ElectronicLevels[1/cm]              1
  0.0000000000000000      1.0000000000000000
 End
!*******************************************
     Tunneling    Eckart
     ImaginaryFrequency[1/cm]    282.13240000000002
     WellDepth[kcal/mol]        4.46
     WellDepth[kcal/mol]        11.06
 End
End
Barrier TS4 W4 Pr1
 RRHO    ! transition state
 Geometry[angstrom]          13
  C  -0.67155284709999996     -1.0775262983000000      0.11003085830000001
  C   -1.2255630918000000      4.5627373200000015E-002  0.80794742570000000
  C  -0.25014004610000001      0.83984133890000001      1.3566787313999999
  C    1.0989652228000000      0.16304237190000001      1.2030120834000000
  C   0.79332642139999998     -0.71697825309999996      6.1159624999999970E-003
  C   0.28723926020000001      8.7836205700000029E-002  -1.0997844873000000
  O  -8.1455698800000037E-002  0.55392830019999995      -2.1123457443999998
  H   1.5219873702000000      -1.4500928237999999     -0.32656756830000000
  H   -1.1687139378000000      -1.8166175471999999     -0.49246730500000002
  H   -2.2847636346000000      0.25209024159999999      0.87276550060000002
  H  -0.42765131410000001      1.6949062170000000       1.9896590092999999
  H   1.3369254581000001     -0.46478184830000002       2.0733121565000001
  H   1.9305918057000000      0.83464400930000004       1.0046681392000001
       Core    RigidRotor
           SymmetryFactor   0.50000000000000000
       End
   Frequencies[1/cm]        32
  116.63    225.55    324.45    424.96    490.30    532.13    660.16    726.77    793.49    867.99
  930.77    936.95    947.65    1005.4    1028.5    1047.3    1105.5    1137.7    1205.3    1253.9
  1277.5    1302.1    1386.8    1483.1    1512.4    2000.4    3008.1    3154.9    3174.8    3231.1
  3260.9    3296.7
ZeroEnergy[kcal/mol] 75.09782736984145
  ElectronicLevels[1/cm]              1
  0.0000000000000000      1.0000000000000000
     Tunneling    Eckart
   ImaginaryFrequency[1/cm]     724.65999999999997
   WellDepth[kcal/mol]        24.57
   WellDepth[kcal/mol]        50.39
```

```
End
End
!***********************************
Barrier TS5 W1 Pr2
Variational
!***** block 1
RRHO    ! transition state
Geometry[angstrom]          13
 C   0.00000  0.00000  0.00000
 C   0.00000  0.00000  1.38752
 C   1.20287  0.00000  2.08827
 C   2.40961 -0.00362  1.39468
 C   2.41778 -0.00306  0.00694
 C   1.21112 -0.00599 -0.69743
 O   1.21614 -0.12512 -2.04595
 H   3.34713 -0.01083 -0.55117
 H  -0.92608 -0.00529 -0.56358
 H  -0.94129 -0.00327  1.92568
 H   1.19980  0.00092  3.17223
 H   3.34774 -0.00944  1.93830
 H   1.19951  1.34139 -2.68554
        Core     RigidRotor
                 SymmetryFactor   1.0000000000000000
        End
  Frequencies[1/cm]        32
  263.71    413.37    425.02    528.26    539.32    632.70    697.46    787.60    840.81    854.90
  910.93    941.09    1001.5    1014.8    1023.5    1050.9    1102.8    1173.9    1184.7    1186.2
  1272.6    1314.1    1347.1    1491.7    1527.3    1649.1    1659.8    3187.1    3195.2    3205.9
  3212.2    3218.3
 ZeroEnergy[kcal/mol]            68.66959369513855
 ElectronicLevels[1/cm]                1
  0.0000000000000000      1.0000000000000000
End
!***** block 2
RRHO    ! transition state
Geometry[angstrom]          13
 C   0.00000  0.00000  0.00000
 C   0.00000  0.00000  1.38644
 C   1.20396  0.00000  2.08660
 C   2.41235 -0.00374  1.39437
 C   2.42132 -0.00292  0.00784
 C   1.21295 -0.01028 -0.70025
 O   1.21802 -0.13499 -2.03650
 H   3.35002 -0.00673 -0.55124
 H  -0.92517 -0.00144 -0.56497
 H  -0.94047 -0.00111  1.92594
 H   1.20037  0.00299  3.17060
 H   3.34932 -0.00774  1.93990
```

```
 H   1.21091   1.41014  -2.74538
          Core      RigidRotor
                    SymmetryFactor    1.0000000000000000
          End
   Frequencies[1/cm]          32
  260.07     412.98     425.42     525.34     539.06     631.39     694.46     788.63     835.94     841.98
  854.35     943.34     1002.6     1015.7     1022.4     1049.3     1089.0     1103.0     1185.0     1185.2
  1285.9     1313.3     1347.3     1491.0     1524.7     1643.3     1657.9     3187.6     3196.1     3206.6
  3213.4     3218.9
 ZeroEnergy[kcal/mol]             75.82959369513855
 ElectronicLevels[1/cm]                  1
  0.0000000000000000       1.0000000000000000
End
!***** block 3
RRHO     ! transition state
Geometry[angstrom]            13
 C   0.00000   0.00000   0.00000
 C   0.00000   0.00000   1.38009
 C   1.20898   0.00000   2.08167
 C   2.42611  -0.00120   1.39433
 C   2.44236  -0.00118   0.01433
 C   1.22543  -0.01423  -0.71757
 O   1.23302  -0.09354  -2.01055
 H   3.37093  -0.00061  -0.54475
 H  -0.92194   0.00147  -0.56994
 H  -0.93812   0.00056   1.92338
 H   1.20261   0.00288   3.16573
 H   3.35777  -0.00162   1.94862
 H   1.23765   1.51671  -2.81497
          Core      RigidRotor
                    SymmetryFactor    1.0000000000000000
          End
   Frequencies[1/cm]          32
  225.90     400.44     424.06     494.75     506.78     530.54     621.23     656.29     688.18     805.20
  839.43     860.42     947.21     1002.8     1005.2     1012.2     1037.9     1103.4     1171.2     1179.9
  1306.2     1316.4     1343.7     1481.0     1490.5     1601.9     1622.2     3189.8     3198.4     3208.8
  3216.6     3220.8
 ZeroEnergy[kcal/mol]             79.38959369513855
 ElectronicLevels[1/cm]                  1
  0.0000000000000000       1.0000000000000000
End
!***** block 4
RRHO     ! transition state
Geometry[angstrom]            13
 C   0.00000   0.00000   0.00000
 C   0.00000   0.00000   1.37503
 C   1.21214   0.00000   2.07961
 C   2.43627   0.00035   1.39611
```

C 2.46013 0.00076 0.02126
C 1.23651 -0.01119 -0.73109
O 1.24754 -0.05567 -1.99570
H 3.38957 0.00274 -0.53623
H -0.91963 0.00155 -0.57354
H -0.93693 0.00082 1.92039
H 1.20276 0.00159 3.16365
H 3.36362 0.00155 1.95761
H 1.24951 1.70504 -2.94434
Core RigidRotor
SymmetryFactor 1.0000000000000000
End
Frequencies[1/cm] 32

193.72	246.26	360.54	392.65	450.42	505.20	539.18	610.24	662.13	801.64
823.91	835.77	940.98	992.22	1002.3	1008.4	1027.9	1102.5	1169.0	1174.1
1297.2	1341.9	1377.8	1467.3	1476.1	1578.8	1611.5	3190.6	3198.8	3209.9
3218.2	3221.9								

ZeroEnergy[kcal/mol] 83.24959369513855
ElectronicLevels[1/cm] 1
0.0000000000000000 1.0000000000000000
End
!***** block 5
RRHO ! transition state
Geometry[angstrom] 13
C 0.00000 0.00000 0.00000
C 0.00000 0.00000 1.37284
C 1.21319 0.00000 2.07951
C 2.44046 -0.00026 1.39759
C 2.46828 -0.00005 0.02505
C 1.24175 -0.00688 -0.73750
O 1.25444 -0.03034 -1.99059
H 3.39847 0.00134 -0.53120
H -0.91873 0.00136 -0.57500
H -0.93668 0.00056 1.91875
H 1.20221 0.00126 3.16351
H 3.36586 0.00010 1.96243
H 1.26729 1.88799 -3.06756
Core RigidRotor
SymmetryFactor 1.0000000000000000
End
Frequencies[1/cm] 32

118.00	157.16	236.10	385.57	451.38	491.85	534.21	604.71	655.08	800.45
815.61	822.59	936.46	988.55	1001.8	1006.2	1023.1	1101.5	1170.7	1170.8
1291.5	1341.1	1418.6	1459.3	1483.6	1571.5	1609.6	3190.5	3198.3	3210.1
3218.3	3221.9								

ZeroEnergy[kcal/mol] 84.64959369513855
ElectronicLevels[1/cm] 1
0.0000000000000000 1.0000000000000000

```
End
!***** block 6
RRHO     ! transition state
Geometry[angstrom]           13
 C   0.00000   0.00000   0.00000
 C   0.00000   0.00000   1.37240
 C   1.21339   0.00000   2.07953
 C   2.44137   0.00056   1.39801
 C   2.47017   0.00018   0.02593
 C   1.24297  -0.00466  -0.73896
 O   1.25609  -0.01977  -1.98950
 H   3.40059   0.00139  -0.52987
 H  -0.91855   0.00111  -0.57520
 H  -0.93656   0.00058   1.91852
 H   1.20201   0.00086   3.16353
 H   3.36626   0.00159   1.96367
 H   1.26969   1.98910  -3.10943
        Core     RigidRotor
                 SymmetryFactor    1.0000000000000000
        End
   Frequencies[1/cm]         32
  74.886    128.15    212.19    383.77    451.99    488.72    533.82    603.38    653.21    799.75
  813.22    819.18    934.90    988.07    1001.4    1005.5    1022.0    1101.1    1169.9    1171.2
  1290.2    1340.8    1426.6    1457.2    1490.3    1570.1    1609.2    3190.6    3198.4    3210.2
  3218.6    3222.2
 ZeroEnergy[kcal/mol]            84.96959369513855
 ElectronicLevels[1/cm]                 1
  0.0000000000000000       1.0000000000000000
End
!***** block 7
RRHO     ! transition state
Geometry[angstrom]           13
 C   0.00000   0.00000   0.00000
 C   0.00000   0.00000   1.37194
 C   1.21353   0.00000   2.07957
 C   2.44209   0.00040   1.39832
 C   2.47176   0.00073   0.02672
 C   1.24406  -0.00357  -0.74039
 O   1.25754  -0.01574  -1.98864
 H   3.40243   0.00197  -0.52872
 H  -0.91843   0.00064  -0.57545
 H  -0.93658   0.00036   1.91808
 H   1.20182   0.00061   3.16355
 H   3.36664   0.00119   1.96459
 H   1.29242   2.05398  -3.20314
        Core     RigidRotor
                 SymmetryFactor    1.0000000000000000
        End
```

```
  Frequencies[1/cm]        32
 10.165     88.355     202.80     382.40     452.15     486.59     533.70     602.25     651.82     799.16
 811.54     816.47     933.71     987.73     1001.2     1004.9     1021.0     1100.9     1169.2     1171.8
 1288.7     1340.7     1431.8     1455.6     1498.5     1569.0     1609.4     3190.5     3198.1     3210.2
 3218.4     3222.0
 ZeroEnergy[kcal/mol]          85.13959369513855
 ElectronicLevels[1/cm]              1
 0.0000000000000000      1.0000000000000000
End
!***** block 8
RRHO    ! transition state
Geometry[angstrom]          13
 C   0.00000  0.00000  0.00000
 C   0.00000  0.00000  1.37162
 C   1.21365  0.00000  2.07968
 C   2.44275  0.00009  1.39877
 C   2.47322  -0.00035  0.02751
 C   1.24501  -0.00161  -0.74149
 O   1.25888  -0.00599  -1.98777
 H   3.40409  -0.00013  -0.52756
 H   -0.91831  0.00048  -0.57561
 H   -0.93654  0.00019  1.91785
 H   1.20159  0.00042  3.16366
 H   3.36693  0.00038  1.96567
 H   1.29715  2.24148  -3.29445
         Core     RigidRotor
                  SymmetryFactor    1.0000000000000000
         End
  Frequencies[1/cm]        32
 20.632     90.487     194.59     380.96     452.62     484.27     533.70     601.23     650.45     798.36
 809.62     813.90     932.24     987.55     1000.9     1004.2     1020.2     1100.6     1168.5     1172.2
 1287.6     1340.5     1435.4     1454.0     1507.9     1568.1     1609.3     3190.4     3197.9     3210.2
 3218.5     3222.1
 ZeroEnergy[kcal/mol]          85.52959369513855
 ElectronicLevels[1/cm]              1
 0.0000000000000000      1.0000000000000000
End
!***** block 9
RRHO    ! transition state
Geometry[angstrom]          13
 C   0.00000  0.00000  0.00000
 C   0.00000  0.00000  1.37162
 C   1.21365  0.00000  2.07968
 C   2.44275  0.00009  1.39877
 C   2.47322  -0.00035  0.02751
 C   1.24501  -0.00161  -0.74149
 O   1.25888  -0.00599  -1.98777
 H   3.40409  -0.00013  -0.52756
```

```
 H   -0.91831   0.00048  -0.57561
 H   -0.93654   0.00019   1.91785
 H    1.20159   0.00042   3.16366
 H    3.36693   0.00038   1.96567
 H    1.29715   2.24148  -3.29445
          Core      RigidRotor
                    SymmetryFactor    1.0000000000000000
          End
   Frequencies[1/cm]          32
  20.632     90.487     194.59     380.96     452.62     484.27     533.70     601.23     650.45     798.36
  809.62     813.90     932.24     987.55     1000.9     1004.2     1020.2     1100.6     1168.5     1172.2
  1287.6     1340.5     1435.4     1454.0     1507.9     1568.1     1609.3     3190.4     3197.9     3210.2
  3218.5     3222.1
 ZeroEnergy[kcal/mol]             85.52959369513855
 ElectronicLevels[1/cm]                   1
  0.0000000000000000       1.0000000000000000
 End
End
Barrier TS6 W2 Pr2
 Variational
 !***** block 1

 RRHO           !            1
 Geometry[angstrom]          13
 C   -1.24840  -0.01164  -0.25866
 C   -1.24821  -0.01202   1.10592
 C   -0.03131  -0.01200   1.82912
 C    1.19004   0.04302   1.16800
 C    1.22951   0.08779  -0.21877
 C    0.00338  -0.04141  -1.00594
 O    0.03254  -0.18547  -2.23113
 H    2.16520   0.08404  -0.76562
 H   -2.16566  -0.03943  -0.83512
 H   -2.18615  -0.02415   1.65008
 H   -0.05651  -0.04089   2.91235
 H    2.11293   0.06974   1.73566
 H    0.86377   2.25437  -0.47974
          Core      RigidRotor
       SymmetryFactor   0.50000000000000000
 End
   Frequencies[1/cm]          32
  3223.7     3219.7     3211.9     3199.2     3190.9     1617.0     1595.1     1551.7     1448.4     1433.5
  1344.3     1281.1     1177.0     1173.5     1092.7     1018.2     1011.5     993.78     988.61     931.34
  817.36     803.15     794.43     668.47     598.64     536.91     491.02     458.19     449.66     373.49
  278.20     176.30
 ZeroEnergy[kcal/mol]    80.576600871755100
 ElectronicLevels[1/cm]                   1
  0.0000000000000000       1.0000000000000000
```

```
End
!******************************************
RRHO         !         2
Geometry[angstrom]        13
C  -1.24831  -0.01170  -0.25874
C  -1.24817  -0.01201   1.10607
C  -0.03158  -0.01197   1.82911
C   1.19020   0.04306   1.16763
C   1.22959   0.08632  -0.21854
C   0.00349  -0.04142  -1.00563
O   0.03256  -0.18541  -2.23119
H   2.16521   0.08440  -0.76554
H  -2.16563  -0.03936  -0.83513
H  -2.18617  -0.02414   1.65011
H  -0.05658  -0.04076   2.91236
H   2.11294   0.06983   1.73557
H   0.86114   2.27000  -0.48145
          Core     RigidRotor
        SymmetryFactor   0.50000000000000000
End
   Frequencies[1/cm]        32
 3223.6    3219.7    3211.8    3199.1    3190.9    1616.1    1591.7    1552.0    1448.0    1433.7
 1343.2    1280.9    1176.8    1173.0    1093.0    1018.3    1011.1    993.82    988.41    930.85
 816.19    803.07    794.57    667.25    598.34    536.62    490.26    457.75    437.55    372.54
 268.92    174.44
ZeroEnergy[kcal/mol]    80.818044204719345
 ElectronicLevels[1/cm]              1
 0.0000000000000000       1.0000000000000000
End
!******************************************
RRHO         !         3
Geometry[angstrom]        13
C  -1.24824  -0.01175  -0.25882
C  -1.24814  -0.01199   1.10621
C  -0.03181  -0.01196   1.82910
C   1.19034   0.04310   1.16732
C   1.22968   0.08488  -0.21832
C   0.00358  -0.04142  -1.00536
O   0.03258  -0.18537  -2.23126
H   2.16524   0.08475  -0.76548
H  -2.16561  -0.03929  -0.83512
H  -2.18619  -0.02413   1.65012
H  -0.05663  -0.04063   2.91236
H   2.11295   0.06991   1.73551
H   0.85852   2.28569  -0.48317
          Core     RigidRotor
        SymmetryFactor   0.50000000000000000
End
```

```
   Frequencies[1/cm]        32
  3223.4     3219.6     3211.8     3199.0     3190.9     1615.4     1588.6     1552.2     1447.6     1433.9
  1342.2     1280.6     1176.6     1172.5     1093.3     1018.4     1010.8     993.89     988.20     930.42
  815.13     802.96     794.70     666.14     598.07     536.38     489.60     457.42     426.03     371.26
  260.56     172.32
ZeroEnergy[kcal/mol]    81.047652818084781
 ElectronicLevels[1/cm]              1
  0.0000000000000000       1.0000000000000000
End
!********************************************
RRHO         !          4
Geometry[angstrom]        13
C  -1.24818  -0.01180  -0.25888
C  -1.24810  -0.01196   1.10632
C  -0.03201  -0.01196   1.82909
C   1.19045   0.04314   1.16707
C   1.22980   0.08344  -0.21812
C   0.00364  -0.04142  -1.00512
O   0.03259  -0.18533  -2.23131
H   2.16527   0.08510  -0.76546
H  -2.16562  -0.03923  -0.83510
H  -2.18623  -0.02412   1.65010
H  -0.05665  -0.04053   2.91235
H   2.11295   0.06996   1.73549
H   0.85591   2.30142  -0.48491
        Core     RigidRotor
       SymmetryFactor   0.50000000000000000
End
   Frequencies[1/cm]        32
  3223.3     3219.5     3211.7     3198.9     3190.9     1614.8     1585.9     1552.3     1447.3     1434.1
  1341.3     1280.3     1176.5     1172.1     1093.5     1018.4     1010.4     993.97     988.00     930.04
  814.15     802.80     794.80     665.12     597.78     536.16     489.00     457.11     415.38     369.42
  252.98     169.91
ZeroEnergy[kcal/mol]    81.266854936843795
 ElectronicLevels[1/cm]              1
  0.0000000000000000       1.0000000000000000
End
!********************************************
RRHO         !          5
Geometry[angstrom]        13
C  -1.24821  -0.01178  -0.25890
C  -1.24805  -0.01192   1.10634
C  -0.03203  -0.01206   1.82907
C   1.19047   0.04320   1.16712
C   1.23004   0.08209  -0.21796
C   0.00360  -0.04136  -1.00510
O   0.03260  -0.18540  -2.23136
H   2.16532   0.08562  -0.76554
```

```
H   -2.16571  -0.03919  -0.83494
H   -2.18629  -0.02412   1.64995
H   -0.05652  -0.04059   2.91231
H    2.11292   0.06988   1.73561
H    0.85338   2.31740  -0.48678
         Core     RigidRotor
       SymmetryFactor   0.50000000000000000
End
   Frequencies[1/cm]        32
 3223.5    3219.8    3211.9    3198.9    3190.9    1614.5    1584.2    1552.0    1447.1    1434.1
 1340.5    1279.7    1176.3    1171.9    1093.5    1018.4    1010.1    993.92    987.76    929.46
 813.03    802.38    794.80    664.08    597.43    535.91    488.32    456.74    406.08    366.73
 246.00    167.20
ZeroEnergy[kcal/mol]    81.476215982531232
 ElectronicLevels[1/cm]              1
 0.0000000000000000       1.0000000000000000
End
!*******************************************
RRHO          !          6
Geometry[angstrom]        13
C    0.00000   0.00000   0.00000
C    0.00000   0.00000   1.36548
C    1.21572   0.00000   2.08799
C    2.43869   0.05514   1.42559
C    2.47816   0.09247   0.04117
C    1.25180  -0.02956  -0.74579
O    1.28068  -0.17331  -1.97249
H    3.41339   0.09763  -0.50655
H   -0.91753  -0.02724  -0.57611
H   -0.93830  -0.01217   1.90899
H    1.19143  -0.02829   3.17128
H    3.36102   0.08200   1.99428
H    2.09872   2.34476  -0.22939
         Core     RigidRotor
       SymmetryFactor   0.50000000000000000
End
   Frequencies[1/cm]        32
 3223.4    3219.7    3211.8    3198.8    3190.9    1613.8    1581.4    1552.4    1446.9    1434.5
 1340.1    1279.7    1176.1    1171.5    1093.8    1018.5    1009.7    994.04    987.65    929.27
 812.28    802.52    794.93    663.23    597.35    535.78    487.88    456.51    398.24    362.51
 239.14    163.82
ZeroEnergy[kcal/mol]    81.674426956939992
 ElectronicLevels[1/cm]              1
 0.0000000000000000       1.0000000000000000
End
!*******************************************
!***** block 2
RRHO    ! transition state
```

```
Geometry[angstrom]          13
C   0.00000  0.00000  0.00000
C   0.00000  0.00000  1.36718
C   1.21496  0.00000  2.08597
C   2.44011  0.04160  1.41831
C   2.47737  0.07067  0.03816
C   1.25003  -0.02428  -0.74547
O   1.27413  -0.13873  -1.97752
H   3.41153  0.07650  -0.51141
H   -0.91773  -0.02137  -0.57610
H   -0.93780  -0.00916  1.91147
H   1.19373  -0.02160  3.16950
H   3.36289  0.06285  1.98670
H   2.09939  2.42555  -0.22951
        Core     RigidRotor
                 SymmetryFactor    0.5000000000000000
        End
  Frequencies[1/cm]        32
 130.97    214.79    317.00    382.38    455.06    486.41    534.53    597.74    658.74    795.39
 804.44    809.79    929.07    987.48    995.24    1007.6    1019.3    1096.2    1169.4    1175.3
 1281.1    1338.7    1437.2    1446.0    1553.8    1567.0    1610.3    3190.8    3198.4    3211.1
 3219.0    3222.8
 ZeroEnergy[kcal/mol]          82.700469720187422
 ElectronicLevels[1/cm]              1
 0.0000000000000000      1.0000000000000000
End
!***** block 3
RRHO     ! transition state
Geometry[angstrom]          13
C   0.00000  0.00000  0.00000
C   0.00000  0.00000  1.36922
C   1.21418  0.00000  2.08315
C   2.44153  0.02596  1.40930
C   2.47604  0.04367  0.03380
C   1.24798  -0.01325  -0.74431
O   1.26721  -0.08105  -1.98317
H   3.40883  0.04976  -0.51809
H   -0.91795  -0.01330  -0.57602
H   -0.93724  -0.00581  1.91438
H   1.19697  -0.01333  3.16692
H   3.36493  0.03981  1.97709
H   2.13095  2.61005  -0.19994
        Core     RigidRotor
                 SymmetryFactor    0.5000000000000000
        End
  Frequencies[1/cm]        32
 62.343    200.27    222.68    379.22    453.68    484.40    533.46    598.56    653.67    796.02
 807.46    807.97    929.31    987.45    997.21    1004.7    1019.8    1098.5    1168.0    1174.1
```

```
 1283.6     1338.6     1439.4     1447.0     1538.6     1564.7     1608.0     3190.6     3198.1     3210.7
 3218.8     3222.4
 ZeroEnergy[kcal/mol]            84.000469720187422
 ElectronicLevels[1/cm]                 1
 0.0000000000000000       1.0000000000000000
End
!***** block 4
RRHO     ! transition state
Geometry[angstrom]           13
C    0.00000   0.00000   0.00000
C    0.00000   0.00000   1.37019
C    1.21387   0.00000   2.08161
C    2.44237   0.01505   1.40450
C    2.47540   0.02626   0.03143
C    1.24710  -0.00793  -0.74372
O    1.26382  -0.04805  -1.98534
H    3.40744   0.03136  -0.52175
H   -0.91805  -0.00811  -0.57598
H   -0.93694  -0.00327   1.91586
H    1.19882  -0.00793   3.16547
H    3.36600   0.02383   1.97212
H    2.12595   2.79408  -0.20742
          Core      RigidRotor
                    SymmetryFactor    0.5000000000000000
          End
   Frequencies[1/cm]         32
 3221.1     3217.2     3207.4     3195.5     3189.0     1606.3     1565.5     1527.3     1447.3     1438.9
 1339.2     1284.1     1173.5     1166.1     1099.2     1019.6     1002.6     987.84     987.33     918.11
 809.07     804.04     784.55     651.06     598.87     533.33     482.19     453.26     378.12     187.57
 166.11     32.870
ZeroEnergy[kcal/mol]    84.650469720187422
 ElectronicLevels[1/cm]                 1
 0.0000000000000000       1.0000000000000000
End
!***** block 5
RRHO     ! transition state
Geometry[angstrom]           13
C    0.00000   0.00000   0.00000
C    0.00000   0.00000   1.37073
C    1.21375   0.00000   2.08090
C    2.44278   0.00864   1.40206
C    2.47497   0.01623   0.03017
C    1.24664  -0.00430  -0.74332
O    1.26211  -0.02722  -1.98627
H    3.40659   0.01976  -0.52367
H   -0.91808  -0.00504  -0.57600
H   -0.93673  -0.00205   1.91670
H    1.19985  -0.00496   3.16482
```

```
H   3.36651  0.01367  1.96965
H   2.11930  2.98497  -0.21496
         Core     RigidRotor
                  SymmetryFactor   0.5000000000000000
         End
   Frequencies[1/cm]        32
  3221.6    3217.9    3209.8    3197.1    3190.1    1607.1    1566.3    1524.5    1448.8    1438.1
  1339.5    1284.7    1173.1    1166.8    1099.4    1019.3    1002.4    990.76    987.61    920.22
  810.11    804.39    784.49    650.03    599.52    533.36    481.56    453.03    376.94    186.10
  122.84    31.460
 ZeroEnergy[kcal/mol]   85.090469720187422
  ElectronicLevels[1/cm]              1
  0.0000000000000000      1.0000000000000000
 End
 !***** block 6
 RRHO    ! transition state
 Geometry[angstrom]          13
 C   0.00000  0.00000  0.00000
 C   0.00000  0.00000  1.37098
 C   1.21374  0.00000  2.08037
 C   2.44303  0.00468  1.40064
 C   2.47482  0.00926  0.02933
 C   1.24636  -0.00248  -0.74307
 O   1.26123  -0.01513  -1.98663
 H   3.40618  0.01115  -0.52491
 H   -0.91812  -0.00274  -0.57594
 H   -0.93665  -0.00110  1.91708
 H   1.20056  -0.00279  3.16431
 H   3.36680  0.00742  1.96822
 H   2.11240  3.17835  -0.22658
         Core     RigidRotor
                  SymmetryFactor   0.5000000000000000
         End
   Frequencies[1/cm]        32
  39.371    83.826    185.31    379.24    452.80    482.31    533.44    599.75    649.50    796.81
  807.67    810.60    930.43    987.62    1000.1    1003.4    1019.5    1100.1    1167.5    1173.1
  1285.7    1339.8    1438.6    1451.2    1522.7    1566.9    1609.1    3190.2    3197.6    3210.2
  3218.4    3222.0
  ZeroEnergy[kcal/mol]          85.430469720187422
  ElectronicLevels[1/cm]              1
  0.0000000000000000      1.0000000000000000
 End
End
Barrier TS7 W6 Pr2
 Variational
 !***** block 1
 RRHO    ! transition state
 Geometry[angstrom]          13
```

```
C    0.00000   0.00000   0.00000
C    0.00000   0.00000   1.33738
C    1.25220   0.00000   2.11265
C    2.50655  -0.05104   1.34255
C    2.51205  -0.05141   0.00519
C    1.25787  -0.01431  -0.77662
O    1.26062  -0.00060  -1.99365
H    3.43313  -0.08450  -0.56558
H   -0.91928   0.00451  -0.57459
H   -0.93250   0.00241   1.89203
H    1.23760  -0.62906   3.00151
H    3.43608  -0.08652   1.90104
H    1.27968   1.42460   2.84049
      Core    RigidRotor
           SymmetryFactor    1.0000000000000000
      End
   Frequencies[1/cm]          32
  3208.9     3178.0     3175.6     3146.3     3034.4     2849.4     1728.8     1695.9     1594.8     1434.3
  1430.1     1391.0     1340.8     1279.7     1215.5     1187.5     1152.8     1034.2     1025.7     1003.6
  967.81     923.23     893.81     783.09     756.10     586.44     578.12     510.31     463.83     363.13
  302.66     117.66
ZeroEnergy[kcal/mol]    53.77831651207145
 ElectronicLevels[1/cm]                  1
  0.0000000000000000        1.0000000000000000
End
!***** block 2
RRHO     ! transition state
Geometry[angstrom]            13
C    0.00000   0.00000   0.00000
C    0.00000   0.00000   1.33827
C    1.25135   0.00000   2.10606
C    2.50556  -0.04628   1.34436
C    2.51204  -0.04663   0.00611
C    1.25813  -0.01148  -0.77574
O    1.26132   0.00321  -1.99298
H    3.43346  -0.07843  -0.56405
H   -0.91918   0.00231  -0.57465
H   -0.93123  -0.00033   1.89455
H    1.23847  -0.57792   3.02718
H    3.43344  -0.08094   1.90514
H    1.27780   1.53380   2.83870
         Core      RigidRotor
                   SymmetryFactor    1.0000000000000000
         End
   Frequencies[1/cm]          32
  3208.9     3178.0     3175.6     3146.3     3034.2     2849.2     1728.8     1695.9     1594.8     1434.3
  1430.0     1390.7     1340.7     1279.7     1215.4     1187.5     1152.7     1034.2     1025.7     1003.3
  967.80     923.22     893.79     783.11     756.09     586.43     578.11     510.31     463.82     363.12
```

```
  302.66     117.66
ZeroEnergy[kcal/mol]    61.23831651207145
 ElectronicLevels[1/cm]                 1
  0.0000000000000000       1.0000000000000000
End
!***** block 3
RRHO     ! transition state
Geometry[angstrom]           13
C    0.00000   0.00000   0.00000
C    0.00000   0.00000   1.33925
C    1.25021   0.00000   2.09990
C    2.50422  -0.03835   1.34657
C    2.51200  -0.03866   0.00735
C    1.25848  -0.00675  -0.77467
O    1.26230   0.00922  -1.99212
H    3.43392  -0.06734  -0.56207
H   -0.91901  -0.00051  -0.57481
H   -0.93013  -0.00352   1.89689
H    1.23926  -0.52714   3.04947
H    3.43058  -0.07022   1.90956
H    1.27293   1.64123   2.83872
          Core      RigidRotor
                    SymmetryFactor    1.0000000000000000
          End
   Frequencies[1/cm]          32
  3221.2     3217.7     3208.2     3197.2     3190.1     1609.4     1565.9     1522.9     1452.7     1437.0
  1341.3     1285.8     1172.9     1167.5     1100.0     1017.6     1000.8     1000.5     986.50     928.81
  810.67     807.30     795.74     647.12     599.64     533.42     479.08     452.81     379.22     184.84
  69.670     64.900
ZeroEnergy[kcal/mol]    67.63831651207145
 ElectronicLevels[1/cm]                 1
  0.0000000000000000       1.0000000000000000
End
!***** block 4
RRHO     ! transition state
Geometry[angstrom]           13
C    0.00000   0.00000   0.00000
C    0.00000   0.00000   1.34157
C    1.24716   0.00000   2.08931
C    2.50051  -0.01806   1.35222
C    2.51189  -0.01803   0.01070
C    1.25934   0.00289  -0.77189
O    1.26459   0.01995  -1.98991
H    3.43498  -0.03767  -0.55698
H   -0.91841  -0.00641  -0.57552
H   -0.92841  -0.01047   1.90119
H    1.23983  -0.42843   3.08514
H    3.42388  -0.04190   1.91970
```

```
H   1.25722   1.85232   2.84352
        Core     RigidRotor
                 SymmetryFactor    1.0000000000000000
        End
   Frequencies[1/cm]        32
  3221.3     3217.7     3208.5     3197.2     3190.3     1609.4     1566.1     1522.9     1452.7     1437.0
  1341.5     1285.8     1172.9     1167.6     1100.1     1017.6     1000.8     1000.5     986.50     928.83
  810.71     807.30     795.77     647.12     599.66     533.41     479.09     452.81     379.23     184.84
  66.930     61.750
ZeroEnergy[kcal/mol]    77.04831651207145
 ElectronicLevels[1/cm]              1
  0.0000000000000000       1.0000000000000000
End
!***** block 5
RRHO     ! transition state
Geometry[angstrom]          13
C   0.00000   0.00000   0.00000
C   0.00000   0.00000   1.36132
C   1.22498   0.00000   2.08056
C   2.46235  -0.02691   1.38337
C   2.48666  -0.02767   0.02227
C   1.25022  -0.00932  -0.75318
O   1.26133  -0.00587  -1.98766
H   3.41501  -0.04970  -0.53658
H  -0.91847  -0.00090  -0.57535
H  -0.93343  -0.00191   1.91271
H   1.21381  -0.13971   3.15474
H   3.38552  -0.04918   1.95133
H   1.24260   2.10680   2.71388
        Core     RigidRotor
                 SymmetryFactor    1.0000000000000000
        End
   Frequencies[1/cm]        32
  3221.6     3217.7     3209.1     3197.2     3190.7     1609.4     1566.3     1522.9     1452.8     1437.0
  1341.7     1285.8     1172.9     1168.0     1100.2     1017.6     1000.9     1000.5     986.49     928.86
  810.71     807.30     795.82     647.14     599.68     533.43     479.11     452.82     379.24     184.85
  64.610     58.930
ZeroEnergy[kcal/mol]    79.66831651207145
 ElectronicLevels[1/cm]              1
  0.0000000000000000       1.0000000000000000
End
!***** block 6
RRHO     ! transition state
Geometry[angstrom]          13
C   0.00000   0.00000   0.00000
C   0.00000   0.00000   1.36610
C   1.21955   0.00000   2.07955
C   2.45325  -0.01320   1.39111
```

```
C   2.48090  -0.01394   0.02528
C   1.24819  -0.00412  -0.74820
O   1.26080  -0.00177  -1.98716
H   3.41071  -0.02597  -0.53149
H  -0.91839  -0.00141  -0.57551
H  -0.93478  -0.00199   1.91524
H   1.20817  -0.07092   3.16063
H   3.37663  -0.02531   1.95907
H   1.21869   2.30474   2.74900
        Core     RigidRotor
                 SymmetryFactor    1.0000000000000000
        End
    Frequencies[1/cm]         32
  3221.7    3217.7    3209.2    3197.2    3190.7    1609.4    1566.3    1522.9    1452.8    1437.0
  1341.8    1285.9    1172.9    1168.0    1100.2    1017.6    1000.9    1000.5    986.49    928.87
  810.73    807.30    795.83    647.15    599.69    533.42    479.10    452.82    379.24    184.85
  62.570    56.670
ZeroEnergy[kcal/mol]    82.16831651207145
 ElectronicLevels[1/cm]                 1
  0.0000000000000000       1.0000000000000000
End
!***** block 7
RRHO     ! transition state
Geometry[angstrom]            13
C   0.00000   0.00000   0.00000
C   0.00000   0.00000   1.36855
C   1.21670   0.00000   2.07947
C   2.44842  -0.00856   1.39498
C   2.47790  -0.00934   0.02674
C   1.24716  -0.00359  -0.74578
O   1.26058  -0.00292  -1.98714
H   3.40833  -0.01686  -0.52904
H  -0.91827  -0.00064  -0.57570
H  -0.93564   0.00021   1.91629
H   1.20491  -0.03739   3.16242
H   3.37202  -0.01490   1.96274
H   1.21251   2.50742   2.76711
        Core     RigidRotor
                 SymmetryFactor    1.0000000000000000
        End
    Frequencies[1/cm]         32
  3221.7    3217.7    3209.2    3197.2    3190.7    1609.4    1566.3    1522.9    1452.8    1437.0
  1341.8    1285.9    1172.9    1168.0    1100.2    1017.6    1000.9    1000.5    986.48    928.87
  810.73    807.31    795.83    647.15    599.69    533.42    479.10    452.82    379.24    184.85
  60.770    54.690
ZeroEnergy[kcal/mol]    83.61831651207145
 ElectronicLevels[1/cm]                 1
  0.0000000000000000       1.0000000000000000
```

```
End
!***** block 8
RRHO     ! transition state
Geometry[angstrom]           13
C    0.00000   0.00000   0.00000
C    0.00000   0.00000   1.36970
C    1.21529   0.00000   2.07953
C    2.44602  -0.00427   1.39685
C    2.47637  -0.00460   0.02749
C    1.24662  -0.00110  -0.74443
O    1.26044   0.00030  -1.98709
H    3.40717  -0.00858  -0.52775
H   -0.91826  -0.00084  -0.57575
H   -0.93602  -0.00034   1.91685
H    1.20323  -0.01955   3.16302
H    3.36967  -0.00793   1.96461
H    1.20561   2.70557   2.80051
         Core      RigidRotor
                   SymmetryFactor    1.0000000000000000
         End
   Frequencies[1/cm]         32
  3221.7    3217.7    3209.2    3197.2    3190.7    1609.4    1566.3    1522.9    1452.8    1437.0
  1341.8    1285.9    1172.9    1168.0    1100.2    1017.6    1000.9    1000.5    986.48    928.87
  810.73    807.31    795.83    647.15    599.69    533.42    479.10    452.81    379.24    184.84
  59.180    52.930
ZeroEnergy[kcal/mol]    84.51831651207145
 ElectronicLevels[1/cm]                 1
  0.0000000000000000       1.0000000000000000
End
!***** block 9
RRHO     ! transition state
Geometry[angstrom]           13
C    0.00000   0.00000   0.00000
C    0.00000   0.00000   1.37036
C    1.21449   0.00000   2.07961
C    2.44468  -0.00387   1.39795
C    2.47557  -0.00448   0.02796
C    1.24636  -0.00155  -0.74372
O    1.26042  -0.00066  -1.98706
H    3.40656  -0.00773  -0.52699
H   -0.91823  -0.00016  -0.57581
H   -0.93629   0.00084   1.91705
H    1.20224  -0.00956   3.16330
H    3.36840  -0.00606   1.96563
H    1.20314   2.90307   2.83595
         Core      RigidRotor
                   SymmetryFactor    1.0000000000000000
         End
```

```
  Frequencies[1/cm]        32
 3221.7    3217.7    3209.2    3197.2    3190.7    1609.4    1566.3    1522.9    1452.8    1437.0
 1341.8    1285.9    1172.9    1168.0    1100.2    1017.6    1000.9    1000.5    986.48    928.87
 810.73    807.31    795.83    647.15    599.69    533.41    479.10    452.81    379.24    184.84
 57.750    51.350
ZeroEnergy[kcal/mol]   85.02831651207145
 ElectronicLevels[1/cm]              1
 0.0000000000000000      1.0000000000000000
End
!***** block 10
RRHO    ! transition state
Geometry[angstrom]          13
C   0.00000  0.00000  0.00000
C   0.00000  0.00000  1.37070
C   1.21408  0.00000  2.07968
C   2.44399 -0.00329  1.39853
C   2.47518 -0.00383  0.02820
C   1.24624 -0.00149 -0.74334
O   1.26042 -0.00090 -1.98703
H   3.40626 -0.00641 -0.52659
H  -0.91821  0.00001 -0.57585
H  -0.93644  0.00121  1.91716
H   1.20173 -0.00493  3.16344
H   3.36775 -0.00466  1.96617
H   1.20093  3.09882  2.87788
        Core     RigidRotor
                 SymmetryFactor   1.0000000000000000
        End
  Frequencies[1/cm]        32
 3221.7    3217.7    3209.2    3197.2    3190.7    1609.4    1566.3    1522.9    1452.8    1437.0
 1341.8    1285.9    1172.9    1168.0    1100.2    1017.6    1000.9    1000.5    986.48    928.87
 810.73    807.31    795.83    647.15    599.69    533.41    479.10    452.81    379.24    184.84
 56.450    49.920
ZeroEnergy[kcal/mol]   85.36831651207145
 ElectronicLevels[1/cm]              1
 0.0000000000000000      1.0000000000000000
End
End
Barrier TS8 W2 W5
Variational
RRHO        !        1
Geometry[angstrom]        13
O   0.63794 -0.14274 -2.24264
C   0.55124 -0.12867 -1.06652
C   1.32597 -0.13878  0.10492
C   0.82056  0.00577  1.39607
C  -0.51593  0.05579  1.79676
C  -1.53229 -0.02649  0.85439
```

C -1.31628 0.38976 -0.46198
H 2.33537 -0.52060 -0.02000
H 1.55874 -0.07406 2.18939
H -0.73405 -0.06752 2.85087
H -2.47583 -0.49225 1.13245
H -0.81123 1.35191 -0.57152
H -2.05890 0.19395 -1.22805
Core RigidRotor
SymmetryFactor 1.0000000000000000
End
Frequencies[1/cm] 32

3214.7	3207.9	3183.0	3167.9	3153.2	3088.2	1949.7	1621.6	1549.8	1515.8
1439.5	1410.0	1256.1	1231.6	1158.2	1103.3	1049.7	1015.3	1011.0	986.42
888.90	845.77	800.09	757.50	690.20	614.37	540.21	465.30	403.00	378.55
299.83	142.33								

ZeroEnergy[kcal/mol] 53.047324699104000
ElectronicLevels[1/cm] 1
0.0000000000000000 1.0000000000000000
End
!***
RRHO ! 2
Geometry[angstrom] 13
O 0.63683 -0.14262 -2.24322
C 0.55859 -0.13014 -1.06763
C 1.32578 -0.13894 0.10426
C 0.81932 0.00581 1.39741
C -0.51459 0.05626 1.79715
C -1.53296 -0.02700 0.85295
C -1.32178 0.39188 -0.45959
H 2.33462 -0.52263 -0.01983
H 1.55844 -0.07266 2.18993
H -0.73390 -0.06406 2.85133
H -2.47347 -0.49921 1.13064
H -0.80407 1.34634 -0.57381
H -2.06289 0.19599 -1.22685
Core RigidRotor
SymmetryFactor 1.0000000000000000
End
Frequencies[1/cm] 32

3216.9	3208.2	3182.9	3167.8	3152.6	3091.0	1958.8	1621.4	1550.7	1516.6
1438.3	1410.1	1258.9	1231.9	1157.3	1103.0	1048.0	1014.0	1009.4	986.45
884.72	842.83	801.17	756.77	685.13	615.06	540.10	465.74	402.44	377.92
297.09	144.54								

ZeroEnergy[kcal/mol] 53.645443615270015
ElectronicLevels[1/cm] 1
0.0000000000000000 1.0000000000000000
End
!***

RRHO ! 3
Geometry[angstrom] 13
O 0.63570 -0.14252 -2.24378
C 0.56598 -0.13159 -1.06876
C 1.32558 -0.13914 0.10361
C 0.81810 0.00585 1.39877
C -0.51325 0.05671 1.79753
C -1.53365 -0.02753 0.85152
C -1.32728 0.39403 -0.45721
H 2.33395 -0.52442 -0.01970
H 1.55817 -0.07113 2.19048
H -0.73375 -0.06050 2.85180
H -2.47111 -0.50608 1.12882
H -0.79723 1.34076 -0.57612
H -2.06687 0.19798 -1.22564
Core RigidRotor
SymmetryFactor 1.0000000000000000
End
Frequencies[1/cm] 32
3219.0 3208.4 3182.9 3167.8 3152.0 3093.9 1968.0 1622.0 1551.6 1517.4
1437.3 1410.1 1261.9 1232.2 1156.5 1102.7 1046.5 1012.8 1007.8 986.35
880.67 839.86 803.74 754.81 680.27 615.34 540.09 466.08 401.95 377.03
294.11 146.60
ZeroEnergy[kcal/mol] 54.176209089720207
ElectronicLevels[1/cm] 1
0.0000000000000000 1.0000000000000000
End
!**
RRHO ! 4
Geometry[angstrom] 13
O 0.63456 -0.14243 -2.24433
C 0.57341 -0.13301 -1.06991
C 1.32538 -0.13939 0.10295
C 0.81692 0.00588 1.40014
C -0.51193 0.05713 1.79790
C -1.53436 -0.02808 0.85009
C -1.33276 0.39624 -0.45486
H 2.33338 -0.52598 -0.01959
H 1.55793 -0.06947 2.19104
H -0.73362 -0.05684 2.85226
H -2.46877 -0.51287 1.12699
H -0.79071 1.33518 -0.57842
H -2.07086 0.19994 -1.22442
Core RigidRotor
SymmetryFactor 1.0000000000000000
End
Frequencies[1/cm] 32
3221.1 3208.5 3182.9 3167.8 3151.6 3096.8 1977.2 1623.2 1552.5 1518.1

1436.3 1410.2 1265.0 1232.5 1156.0 1102.3 1045.1 1011.7 1006.4 986.10
876.76 836.89 807.62 751.84 675.73 615.12 540.12 466.28 401.53 375.84
290.86 148.48
ZeroEnergy[kcal/mol] 54.633346027849971
ElectronicLevels[1/cm] 1
0.0000000000000000 1.0000000000000000
End
!***
RRHO ! 5
Geometry[angstrom] 13
O 0.63340 -0.14236 -2.24486
C 0.58087 -0.13441 -1.07107
C 1.32518 -0.13967 0.10231
C 0.81578 0.00590 1.40152
C -0.51062 0.05753 1.79827
C -1.53509 -0.02865 0.84868
C -1.33824 0.39850 -0.45252
H 2.33291 -0.52729 -0.01951
H 1.55772 -0.06769 2.19161
H -0.73350 -0.05309 2.85273
H -2.46644 -0.51958 1.12516
H -0.78455 1.32964 -0.58070
H -2.07488 0.20186 -1.22319
Core RigidRotor
SymmetryFactor 1.0000000000000000
End
Frequencies[1/cm] 32
3223.0 3208.7 3183.0 3167.8 3151.2 3099.6 1986.4 1625.2 1553.7 1518.6
1435.5 1410.3 1268.1 1232.9 1155.6 1101.7 1043.9 1010.6 1005.3 985.66
873.02 833.97 812.54 748.20 671.60 614.31 540.18 466.33 401.18 374.35
287.40 150.11
ZeroEnergy[kcal/mol] 55.023415438611398
ElectronicLevels[1/cm] 1
0.0000000000000000 1.0000000000000000
End
!***
RRHO ! 6
Geometry[angstrom] 13
O 0.63222 -0.14231 -2.24539
C 0.58837 -0.13580 -1.07226
C 1.32500 -0.14000 0.10168
C 0.81466 0.00592 1.40291
C -0.50934 0.05791 1.79863
C -1.53583 -0.02925 0.84729
C -1.34371 0.40081 -0.45021
H 2.33257 -0.52838 -0.01942
H 1.55755 -0.06579 2.19219
H -0.73340 -0.04924 2.85319

H -2.46413 -0.52622 1.12334
H -0.77875 1.32415 -0.58297
H -2.07894 0.20378 -1.22193
Core RigidRotor
SymmetryFactor 1.0000000000000000
End
Frequencies[1/cm] 32

3224.8	3208.8	3183.2	3167.8	3150.9	3102.3	1995.7	1627.7	1555.1	1519.0
1434.8	1410.5	1271.4	1233.3	1155.5	1101.1	1042.9	1009.6	1004.5	985.08
869.46	831.17	818.26	744.22	667.98	612.84	540.26	466.24	400.88	372.60
283.79	151.49								

ZeroEnergy[kcal/mol] 55.347174995025772
ElectronicLevels[1/cm] 1
0.0000000000000000 1.0000000000000000
End
!***
RRHO ! 7
Geometry[angstrom] 13
O 0.63104 -0.14228 -2.24592
C 0.59589 -0.13716 -1.07347
C 1.32483 -0.14038 0.10107
C 0.81359 0.00594 1.40429
C -0.50809 0.05827 1.79898
C -1.53660 -0.02987 0.84593
C -1.34918 0.40318 -0.44793
H 2.33234 -0.52923 -0.01933
H 1.55741 -0.06377 2.19278
H -0.73330 -0.04530 2.85365
H -2.46184 -0.53279 1.12153
H -0.77334 1.31874 -0.58520
H -2.08304 0.20568 -1.22064
Core RigidRotor
SymmetryFactor 1.0000000000000000
End
Frequencies[1/cm] 32

3226.5	3208.8	3183.3	3167.8	3150.7	3105.0	2005.0	1630.7	1557.0	1519.0
1434.3	1410.7	1274.7	1233.7	1155.5	1100.5	1042.1	1008.7	1004.0	984.40
866.13	828.85	824.29	740.14	664.93	610.69	540.34	466.01	400.62	370.63
280.16	152.58								

ZeroEnergy[kcal/mol] 55.605482440135785
ElectronicLevels[1/cm] 1
0.0000000000000000 1.0000000000000000
End
!***
RRHO ! 8
Geometry[angstrom] 13
O 0.62984 -0.14227 -2.24644
C 0.60345 -0.13851 -1.07471

C 1.32468 -0.14080 0.10049
C 0.81256 0.00595 1.40567
C -0.50688 0.05861 1.79932
C -1.53739 -0.03052 0.84460
C -1.35465 0.40560 -0.44568
H 2.33224 -0.52985 -0.01920
H 1.55730 -0.06162 2.19337
H -0.73322 -0.04127 2.85411
H -2.45957 -0.53930 1.11972
H -0.76833 1.31344 -0.58740
H -2.08721 0.20759 -1.21932
Core RigidRotor
SymmetryFactor 1.0000000000000000
End
Frequencies[1/cm] 32

3228.2	3208.9	3183.5	3167.8	3150.5	3107.6	2014.3	1634.2	1559.3	1518.8
1434.0	1411.1	1278.0	1234.2	1155.8	1099.9	1041.6	1007.9	1003.8	983.71
863.14	832.35	825.13	736.18	662.45	607.92	540.37	465.67	400.38	368.50
276.61	153.39								

ZeroEnergy[kcal/mol] 55.799124038397491
ElectronicLevels[1/cm] 1
0.0000000000000000 1.0000000000000000
End
!***
RRHO ! 9
Geometry[angstrom] 13
O 0.62864 -0.14229 -2.24695
C 0.61103 -0.13984 -1.07597
C 1.32457 -0.14128 0.09994
C 0.81158 0.00596 1.40704
C -0.50571 0.05893 1.79964
C -1.53819 -0.03121 0.84330
C -1.36012 0.40809 -0.44345
H 2.33228 -0.53025 -0.01903
H 1.55722 -0.05935 2.19397
H -0.73315 -0.03715 2.85455
H -2.45730 -0.54576 1.11793
H -0.76375 1.30828 -0.58956
H -2.09146 0.20952 -1.21796
Core RigidRotor
SymmetryFactor 1.0000000000000000
End
Frequencies[1/cm] 32

3229.6	3208.9	3183.8	3167.7	3150.4	3109.9	2023.6	1638.0	1562.2	1518.3
1433.9	1411.5	1281.3	1234.7	1156.2	1099.4	1041.4	1007.1	1003.9	983.09
860.77	839.25	822.84	732.49	660.41	604.64	540.31	465.22	400.14	366.29
273.16	153.92								

ZeroEnergy[kcal/mol] 55.934460658719563

ElectronicLevels[1/cm] 1
0.0000000000000000 1.0000000000000000
End
!***
RRHO ! 10
Geometry[angstrom] 13
O 0.62751 -0.14236 -2.24749
C 0.61862 -0.14113 -1.07726
C 1.32448 -0.14180 0.09941
C 0.81064 0.00598 1.40839
C -0.50461 0.05922 1.79995
C -1.53901 -0.03194 0.84207
C -1.36562 0.41067 -0.44125
H 2.33246 -0.53043 -0.01884
H 1.55718 -0.05695 2.19456
H -0.73312 -0.03291 2.85495
H -2.45501 -0.55224 1.11618
H -0.75963 1.30324 -0.59166
H -2.09584 0.21152 -1.21653
Core RigidRotor
SymmetryFactor 1.0000000000000000
End
Frequencies[1/cm] 32

3231.2	3209.2	3184.1	3167.6	3150.5	3112.8	2032.6	1641.7	1565.6	1517.5
1433.9	1412.0	1284.5	1235.1	1156.7	1099.3	1041.0	1006.0	1003.5	982.64
859.93	844.89	820.03	729.26	658.75	600.78	540.60	464.47	399.24	362.77
268.83	152.11								

ZeroEnergy[kcal/mol] 56.017124088424428
ElectronicLevels[1/cm] 1
0.0000000000000000 1.0000000000000000
End
!***
RRHO ! 11
Geometry[angstrom] 13
O 0.00000 0.00000 0.00000
C 0.00000 0.00000 1.16940
C 0.69822 0.00000 2.34697
C 0.18352 0.14841 3.65774
C -1.12977 0.20192 4.04823
C -2.16611 0.10970 3.08885
C -1.99734 0.55571 1.80889
H 1.70663 -0.38785 2.22954
H 0.93091 0.08806 4.44321
H -1.35932 0.11387 5.10341
H -3.07906 -0.41618 3.36242
H -1.38238 1.44100 1.65430
H -2.72659 0.35595 1.03296
Core RigidRotor

SymmetryFactor 1.0000000000000000
End
Frequencies[1/cm] 32
3232.8 3209.3 3184.3 3167.5 3150.9 3117.0 2042.0 1645.9 1569.4 1516.5
1434.3 1412.5 1287.7 1235.6 1157.5 1098.4 1041.5 1005.7 1004.3 982.17
863.17 847.55 818.24 726.28 656.79 597.49 539.65 464.10 399.63 361.85
266.31 154.22
ZeroEnergy[kcal/mol] 56.051388021683228
ElectronicLevels[1/cm] 1
0.0000000000000000 1.0000000000000000
End
!**
RRHO ! 12
Geometry[angstrom] 13
O 0.62502 -0.14247 -2.24852
C 0.63391 -0.14370 -1.07994
C 1.32449 -0.14302 0.09851
C 0.80890 0.00600 1.41106
C -0.50251 0.05978 1.80050
C -1.54071 -0.03349 0.83968
C -1.37655 0.41592 -0.43697
H 2.33334 -0.53011 -0.01811
H 1.55715 -0.05177 2.19581
H -0.73307 -0.02418 2.85578
H -2.45058 -0.56494 1.11271
H -0.75267 1.29384 -0.59573
H -2.10483 0.21554 -1.21353
Core RigidRotor
SymmetryFactor 1.0000000000000000
End
Frequencies[1/cm] 32
3233.8 3209.2 3184.6 3167.4 3150.7 3118.4 2051.1 1650.4 1573.7 1515.2
1434.9 1413.2 1290.8 1236.0 1158.3 1097.7 1042.0 1005.6 1004.6 981.82
869.65 846.86 816.49 724.00 654.74 593.86 538.23 463.26 399.74 360.78
263.21 155.43
ZeroEnergy[kcal/mol] 56.037781400039169
ElectronicLevels[1/cm] 1
0.0000000000000000 1.0000000000000000
End
!**
RRHO ! 13
Geometry[angstrom] 13
O 0.62367 -0.14253 -2.24903
C 0.64160 -0.14498 -1.08134
C 1.32460 -0.14372 0.09812
C 0.80811 0.00600 1.41236
C -0.50151 0.06003 1.80075
C -1.54158 -0.03432 0.83855

```
C  -1.38197  0.41860  -0.43489
H   2.33406  -0.52958  -0.01757
H   1.55716  -0.04902   2.19643
H  -0.73303  -0.01971   2.85620
H  -2.44843  -0.57116   1.11104
H  -0.74989   1.28953  -0.59765
H  -2.10947   0.21758  -1.21193
          Core     RigidRotor
        SymmetryFactor    1.0000000000000000
End
   Frequencies[1/cm]          32
  3234.9     3208.9     3184.8     3167.3     3150.7     3121.1     2060.1     1654.4     1578.4     1513.9
  1435.3     1413.8     1293.7     1236.5     1159.1     1097.6     1042.4     1005.4     1003.9     981.94
  877.14     844.83     814.26     721.73     652.76     590.06     537.76     462.57     398.88     357.43
  258.71     153.28
ZeroEnergy[kcal/mol]    55.985494919116751
 ElectronicLevels[1/cm]                  1
  0.0000000000000000       1.0000000000000000
End
!*******************************************
RRHO          !          14
Geometry[angstrom]          13
O   0.62238  -0.14263  -2.24954
C   0.64929  -0.14622  -1.08276
C   1.32476  -0.14446   0.09777
C   0.80737   0.00601   1.41362
C  -0.50060   0.06026   1.80097
C  -1.54248  -0.03518   0.83749
C  -1.38744   0.42136  -0.43284
H   2.33496  -0.52882  -0.01695
H   1.55720  -0.04615   2.19705
H  -0.73303  -0.01517   2.85656
H  -2.44628  -0.57735   1.10942
H  -0.74757   1.28543  -0.59950
H  -2.11428   0.21971  -1.21026
          Core     RigidRotor
        SymmetryFactor    1.0000000000000000
End
   Frequencies[1/cm]          32
  3236.2     3208.8     3185.0     3167.1     3151.0     3124.2     2068.9     1658.5     1583.3     1512.5
  1436.0     1414.4     1296.4     1237.0     1159.9     1097.2     1043.1     1005.6     1003.3     982.00
  884.20     842.67     812.40     719.98     650.29     586.73     536.38     461.82     398.30     355.03
  254.61     152.43
ZeroEnergy[kcal/mol]    55.888625172963370
 ElectronicLevels[1/cm]                  1
  0.0000000000000000       1.0000000000000000
End
!*******************************************
```

```
RRHO          !          15
Geometry[angstrom]         13
O   0.62111  -0.14276  -2.25005
C   0.65699  -0.14744  -1.08421
C   1.32499  -0.14526   0.09746
C   0.80667   0.00602   1.41484
C  -0.49975   0.06047   1.80116
C  -1.54339  -0.03608   0.83649
C  -1.39292   0.42416  -0.43082
H   2.33602  -0.52786  -0.01622
H   1.55725  -0.04319   2.19765
H  -0.73304  -0.01059   2.85690
H  -2.44418  -0.58346   1.10787
H  -0.74569   1.28158  -0.60129
H  -2.11922   0.22192  -1.20852
           Core     RigidRotor
        SymmetryFactor    1.0000000000000000
End
   Frequencies[1/cm]         32
  3237.3     3208.8     3185.2     3166.9     3151.2     3127.0     2077.6     1662.5     1588.2     1511.1
  1436.8     1415.1     1298.9     1237.4     1160.8     1096.8     1044.0     1005.7     1002.8     982.21
  890.75     840.50     810.66     718.50     647.58     583.67     534.73     461.12     397.59     352.44
  250.58     151.38
ZeroEnergy[kcal/mol]    55.766526386935607
 ElectronicLevels[1/cm]                 1
  0.0000000000000000       1.0000000000000000
End
!*********************************************
RRHO          !          16
Geometry[angstrom]         13
O   0.61986  -0.14291  -2.25055
C   0.66468  -0.14865  -1.08569
C   1.32529  -0.14611   0.09720
C   0.80601   0.00604   1.41601
C  -0.49896   0.06067   1.80132
C  -1.54431  -0.03701   0.83554
C  -1.39841   0.42702  -0.42882
H   2.33726  -0.52669  -0.01536
H   1.55731  -0.04013   2.19824
H  -0.73306  -0.00597   2.85720
H  -2.44212  -0.58948   1.10638
H  -0.74424   1.27800  -0.60299
H  -2.12432   0.22420  -1.20670
           Core     RigidRotor
        SymmetryFactor    1.0000000000000000
End
   Frequencies[1/cm]         32
  3238.3     3208.7     3185.5     3166.7     3151.4     3129.7     2086.2     1666.4     1593.3     1509.7
```

1437.7 1415.7 1301.2 1237.8 1161.7 1096.5 1045.2 1005.9 1002.2 982.59
896.72 838.41 809.06 717.23 644.72 580.97 532.88 460.48 396.81 349.67
246.86 150.23
ZeroEnergy[kcal/mol] 55.613623956580622
ElectronicLevels[1/cm] 1
0.0000000000000000 1.0000000000000000
End
!***
RRHO ! 17
Geometry[angstrom] 13
O 0.61862 -0.14309 -2.25106
C 0.67235 -0.14984 -1.08719
C 1.32567 -0.14701 0.09697
C 0.80541 0.00605 1.41714
C -0.49824 0.06084 1.80145
C -1.54526 -0.03797 0.83465
C -1.40394 0.42992 -0.42685
H 2.33868 -0.52531 -0.01438
H 1.55737 -0.03698 2.19882
H -0.73310 -0.00134 2.85746
H -2.44011 -0.59542 1.10495
H -0.74323 1.27470 -0.60462
H -2.12957 0.22655 -1.20480
Core RigidRotor
SymmetryFactor 1.0000000000000000
End
Frequencies[1/cm] 32
3239.3 3208.6 3185.7 3166.5 3151.7 3132.3 2094.3 1670.1 1598.4 1508.3
1438.6 1416.2 1303.4 1238.2 1162.6 1096.2 1046.5 1006.0 1001.6 983.17
901.87 836.43 807.56 716.14 641.78 578.65 530.86 459.91 395.83 346.74
243.43 148.99
ZeroEnergy[kcal/mol] 55.428745633073132
ElectronicLevels[1/cm] 1
0.0000000000000000 1.0000000000000000
End
!***
RRHO ! 18
Geometry[angstrom] 13
O 0.61741 -0.14330 -2.25155
C 0.68000 -0.15102 -1.08872
C 1.32614 -0.14796 0.09679
C 0.80484 0.00608 1.41822
C -0.49757 0.06100 1.80155
C -1.54621 -0.03896 0.83381
C -1.40949 0.43287 -0.42490
H 2.34028 -0.52375 -0.01327
H 1.55743 -0.03376 2.19939
H -0.73315 0.00331 2.85769

H -2.43814 -0.60128 1.10359
H -0.74267 1.27171 -0.60615
H -2.13498 0.22899 -1.20280
Core RigidRotor
SymmetryFactor 1.0000000000000000
End
Frequencies[1/cm] 32
3240.3 3208.4 3185.8 3166.2 3151.9 3134.8 2102.1 1673.6 1603.5 1506.9
1439.6 1416.7 1305.4 1238.5 1163.5 1096.0 1048.1 1006.0 1001.1 983.96
906.02 834.57 806.17 715.19 638.84 576.70 528.70 459.43 394.50 343.70
240.07 147.58
ZeroEnergy[kcal/mol] 55.229558747118926
ElectronicLevels[1/cm] 1
0.0000000000000000 1.0000000000000000
End
!***
RRHO ! 19
Geometry[angstrom] 13
O 0.61623 -0.14352 -2.25203
C 0.68763 -0.15219 -1.09026
C 1.32671 -0.14895 0.09663
C 0.80432 0.00611 1.41925
C -0.49696 0.06114 1.80160
C -1.54719 -0.03998 0.83303
C -1.41506 0.43586 -0.42298
H 2.34205 -0.52200 -0.01202
H 1.55749 -0.03046 2.19994
H -0.73321 0.00795 2.85787
H -2.43622 -0.60706 1.10230
H -0.74257 1.26904 -0.60760
H -2.14057 0.23151 -1.20072
Core RigidRotor
SymmetryFactor 1.0000000000000000
End
Frequencies[1/cm] 32
3241.2 3208.3 3186.0 3165.9 3152.2 3137.2 2109.6 1676.9 1608.4 1505.6
1440.5 1417.1 1307.2 1238.9 1164.3 1095.8 1050.0 1006.1 1000.6 984.94
909.40 832.83 804.87 714.34 636.00 575.09 526.48 459.03 393.07 340.66
236.79 146.05
ZeroEnergy[kcal/mol] 55.005128525572904
ElectronicLevels[1/cm] 1
0.0000000000000000 1.0000000000000000
End
!***
RRHO ! 20
Geometry[angstrom] 13
O 0.61506 -0.14377 -2.25250
C 0.69522 -0.15335 -1.09181

C 1.32737 -0.14999 0.09652
C 0.80384 0.00615 1.42021
C -0.49641 0.06126 1.80162
C -1.54817 -0.04103 0.83230
C -1.42067 0.43890 -0.42108
H 2.34401 -0.52009 -0.01064
H 1.55754 -0.02710 2.20048
H -0.73327 0.01258 2.85801
H -2.43434 -0.61275 1.10109
H -0.74290 1.26671 -0.60896
H -2.14632 0.23411 -1.19854
Core RigidRotor
SymmetryFactor 1.0000000000000000
End
Frequencies[1/cm] 32

3242.1	3208.2	3186.1	3165.6	3152.5	3139.4	2116.7	1680.0	1613.3	1504.3
1441.5	1417.5	1308.8	1239.2	1165.0	1095.7	1052.4	1006.2	1000.2	986.05
912.18	831.22	803.68	713.58	633.38	573.82	524.28	458.77	391.80	337.64
233.72	144.53								

ZeroEnergy[kcal/mol] 54.769449025099544
ElectronicLevels[1/cm] 1
0.0000000000000000 1.0000000000000000
End
!**
RRHO ! 21
Geometry[angstrom] 13
O 0.61394 -0.14404 -2.25296
C 0.70277 -0.15451 -1.09337
C 1.32813 -0.15107 0.09643
C 0.80339 0.00620 1.42112
C -0.49590 0.06137 1.80160
C -1.54917 -0.04210 0.83162
C -1.42630 0.44197 -0.41919
H 2.34612 -0.51801 -0.00912
H 1.55758 -0.02369 2.20099
H -0.73334 0.01719 2.85810
H -2.43250 -0.61837 1.09993
H -0.74365 1.26471 -0.61022
H -2.15225 0.23681 -1.19627
Core RigidRotor
SymmetryFactor 1.0000000000000000
End
Frequencies[1/cm] 32

3242.9	3208.0	3186.2	3165.3	3152.8	3141.5	2123.6	1682.9	1618.0	1503.1
1442.5	1417.8	1310.4	1239.5	1165.8	1095.8	1055.2	1006.4	999.76	987.14
914.25	829.73	802.58	712.91	631.02	572.86	522.19	458.66	390.64	334.63
230.84	143.02								

ZeroEnergy[kcal/mol] 54.522019895590515

```
 ElectronicLevels[1/cm]                 1
  0.0000000000000000       1.0000000000000000
 End
!*******************************************
     Tunneling    Eckart
     ImaginaryFrequency[1/cm]    609.87680000000000
     WellDepth[kcal/mol]        38.20
     WellDepth[kcal/mol]        14.12
 End
End
Barrier TS9 W3 W6
 Variational
 RRHO          !          1
 Geometry[angstrom]         13
 C   -1.21141  -0.05339  -0.22060
 C   -1.21068  -0.05216   1.14784
 C   -0.00148  -0.04100   1.86721
 C    1.23302   0.02268   1.10130
 C    1.21766   0.00678  -0.30189
 C   -0.00904   0.00740  -1.05996
 O   -0.06813   0.03273  -2.29054
 H    2.15381   0.01784  -0.84676
 H   -2.15455  -0.07031  -0.75779
 H   -2.14685  -0.06696   1.69478
 H    0.03841  -0.05806   2.94828
 H    2.16589  -0.14117   1.63224
 H    0.80948   1.10533   1.51093
          Core      RigidRotor
        SymmetryFactor   0.50000000000000000
 End
   Frequencies[1/cm]         32
  3235.8     3219.7     3206.3     3189.5     3184.0     2226.1     1712.8     1604.2     1522.7     1502.9
  1424.4     1375.7     1282.6     1223.3     1185.3     1153.6     1074.4     1015.2     1014.1     988.27
  970.98     878.93     818.33     813.07     749.67     646.52     595.39     527.31     493.38     450.13
  390.92     139.30
 ZeroEnergy[kcal/mol]    61.0088675842295500
 ElectronicLevels[1/cm]                 1
  0.0000000000000000       1.0000000000000000
 End
!*******************************************
 RRHO          !          2
 Geometry[angstrom]         13
 C   -1.21111  -0.05340  -0.22075
 C   -1.21044  -0.05224   1.14837
 C   -0.00230  -0.04232   1.86725
 C    1.23263   0.02398   1.10224
 C    1.21738   0.00669  -0.30226
 C   -0.00905   0.00746  -1.06002
```

```
O   -0.06814  0.03271  -2.29075
H   2.15364  0.01783  -0.84698
H   -2.15434  -0.07016  -0.75786
H   -2.14689  -0.06669   1.69487
H   0.03759  -0.05636   2.94839
H   2.16537  -0.14400   1.63229
H   0.82231   1.10789   1.50331
         Core     RigidRotor
       SymmetryFactor   0.5000000000000000
End
   Frequencies[1/cm]        32
  3235.7     3219.5     3206.2     3189.3     3183.0     2248.7     1712.1     1602.0     1522.4     1502.4
  1424.4     1375.2     1282.6     1225.1     1184.8     1155.0     1074.8     1015.4     1009.8     987.72
  969.30     879.94     817.32     812.56     747.35     645.57     595.33     527.25     493.41     449.45
  389.59     138.33
ZeroEnergy[kcal/mol]    61.2571709284754262
 ElectronicLevels[1/cm]              1
  0.0000000000000000       1.0000000000000000
End
!*******************************************
RRHO          !          3
Geometry[angstrom]        13
C   -1.21080  -0.05341  -0.22091
C   -1.21020  -0.05233   1.14891
C   -0.00311  -0.04362   1.86730
C   1.23225   0.02529   1.10319
C   1.21709   0.00660  -0.30264
C   -0.00906   0.00751  -1.06008
O   -0.06815   0.03270  -2.29097
H   2.15347   0.01784  -0.84720
H   -2.15412  -0.07001  -0.75792
H   -2.14693  -0.06642   1.69496
H   0.03674  -0.05474   2.94850
H   2.16484  -0.14689   1.63233
H   0.83516   1.11052   1.49573
         Core     RigidRotor
       SymmetryFactor   0.5000000000000000
End
   Frequencies[1/cm]        32
  3235.5     3219.4     3205.9     3189.1     3181.9     2272.1     1711.5     1599.9     1522.3     1501.7
  1424.3     1374.8     1282.6     1227.2     1184.4     1156.4     1075.2     1015.6     1006.2     987.21
  967.19     880.94     816.85     811.23     745.16     644.46     595.26     527.16     493.32     448.76
  388.18     137.27
ZeroEnergy[kcal/mol]    61.4748850641547349
 ElectronicLevels[1/cm]              1
  0.0000000000000000       1.0000000000000000
End
!*******************************************
```

RRHO ! 4
Geometry[angstrom] 13
C -1.21049 -0.05341 -0.22107
C -1.20997 -0.05242 1.14945
C -0.00392 -0.04491 1.86735
C 1.23186 0.02659 1.10413
C 1.21680 0.00650 -0.30301
C -0.00907 0.00757 -1.06015
O -0.06816 0.03268 -2.29118
H 2.15329 0.01788 -0.84743
H -2.15389 -0.06985 -0.75799
H -2.14699 -0.06616 1.69503
H 0.03587 -0.05318 2.94860
H 2.16429 -0.14984 1.63236
H 0.84803 1.11322 1.48818
Core RigidRotor
SymmetryFactor 0.5000000000000000
End
Frequencies[1/cm] 32

3235.3	3219.3	3205.7	3188.9	3180.8	2295.7	1710.8	1597.8	1522.5	1500.7
1424.3	1374.4	1282.6	1229.5	1183.9	1157.6	1075.6	1015.9	1003.3	986.74
964.65	881.92	816.83	809.15	743.05	643.21	595.19	527.06	493.13	448.08
386.72	136.12								

ZeroEnergy[kcal/mol] 61.6611236567898984
ElectronicLevels[1/cm] 1
0.0000000000000000 1.0000000000000000
End
!***
RRHO ! 5
Geometry[angstrom] 13
C -1.21018 -0.05342 -0.22123
C -1.20973 -0.05253 1.14999
C -0.00473 -0.04617 1.86739
C 1.23148 0.02789 1.10508
C 1.21651 0.00641 -0.30339
C -0.00909 0.00762 -1.06022
O -0.06817 0.03267 -2.29141
H 2.15312 0.01795 -0.84766
H -2.15367 -0.06968 -0.75807
H -2.14705 -0.06591 1.69511
H 0.03498 -0.05169 2.94870
H 2.16372 -0.15286 1.63238
H 0.86091 1.11599 1.48066
Core RigidRotor
SymmetryFactor 0.5000000000000000
End
Frequencies[1/cm] 32

3235.1	3219.1	3205.5	3188.8	3179.6	2319.4	1710.2	1595.6	1522.9	1499.6

1424.3 1374.2 1282.6 1231.9 1183.5 1158.7 1075.9 1016.2 1000.9 986.33
961.81 882.84 817.04 806.54 741.02 641.83 595.10 526.96 492.82 447.39
385.20 134.91
ZeroEnergy[kcal/mol] 61.8157437492071451
ElectronicLevels[1/cm] 1
0.0000000000000000 1.0000000000000000
End
!***
RRHO ! 6
Geometry[angstrom] 13
C -1.20986 -0.05342 -0.22139
C -1.20950 -0.05265 1.15053
C -0.00553 -0.04742 1.86745
C 1.23110 0.02918 1.10603
C 1.21622 0.00630 -0.30376
C -0.00911 0.00767 -1.06029
O -0.06818 0.03265 -2.29163
H 2.15294 0.01805 -0.84789
H -2.15344 -0.06951 -0.75814
H -2.14711 -0.06566 1.69517
H 0.03406 -0.05027 2.94881
H 2.16313 -0.15595 1.63239
H 0.87382 1.11880 1.47318
Core RigidRotor
SymmetryFactor 0.50000000000000000
End
Frequencies[1/cm] 32
3234.8 3218.9 3205.3 3188.6 3178.3 2343.6 1709.6 1593.5 1523.5 1498.3
1424.2 1374.0 1282.6 1234.4 1183.1 1159.6 1076.2 1016.5 998.94 986.01
958.85 883.71 817.33 803.56 739.04 640.37 595.01 526.86 492.40 446.72
383.63 133.64
ZeroEnergy[kcal/mol] 61.9456494475757196
ElectronicLevels[1/cm] 1
0.0000000000000000 1.0000000000000000
End
!***
RRHO ! 7
Geometry[angstrom] 13
C -1.20955 -0.05342 -0.22155
C -1.20926 -0.05277 1.15108
C -0.00633 -0.04865 1.86751
C 1.23073 0.03048 1.10697
C 1.21592 0.00620 -0.30412
C -0.00913 0.00772 -1.06037
O -0.06818 0.03263 -2.29186
H 2.15275 0.01818 -0.84812
H -2.15321 -0.06934 -0.75822
H -2.14719 -0.06543 1.69523

H 0.03312 -0.04892 2.94891
H 2.16252 -0.15911 1.63238
H 0.88674 1.12167 1.46574
Core RigidRotor
SymmetryFactor 0.50000000000000000
End
Frequencies[1/cm] 32

3234.5	3218.8	3205.1	3188.4	3177.1	2368.3	1709.0	1591.5	1524.2	1496.9
1424.1	1373.8	1282.6	1237.1	1182.7	1160.4	1076.4	1016.8	997.39	985.76
955.95	884.52	817.67	800.29	737.10	638.86	594.91	526.77	491.88	446.05
382.03	132.35								

ZeroEnergy[kcal/mol] 62.0577591537821589
ElectronicLevels[1/cm] 1
0.0000000000000000 1.0000000000000000
End
!**
RRHO ! 8
Geometry[angstrom] 13
C -1.20923 -0.05341 -0.22171
C -1.20903 -0.05291 1.15162
C -0.00712 -0.04986 1.86757
C 1.23036 0.03179 1.10792
C 1.21562 0.00609 -0.30449
C -0.00916 0.00776 -1.06046
O -0.06819 0.03262 -2.29209
H 2.15257 0.01835 -0.84836
H -2.15298 -0.06916 -0.75830
H -2.14726 -0.06522 1.69529
H 0.03216 -0.04764 2.94901
H 2.16188 -0.16234 1.63237
H 0.89968 1.12458 1.45834
Core RigidRotor
SymmetryFactor 0.50000000000000000
End
Frequencies[1/cm] 32

3234.2	3218.6	3204.9	3188.2	3175.7	2393.3	1708.5	1589.5	1525.1	1495.4
1424.0	1373.7	1282.7	1239.8	1182.3	1161.1	1076.6	1017.1	996.11	985.58
953.23	885.25	818.02	796.78	735.17	637.29	594.81	526.70	491.25	445.38
380.38	131.02								

ZeroEnergy[kcal/mol] 62.1387507098790066
ElectronicLevels[1/cm] 1
0.0000000000000000 1.0000000000000000
End
!**
RRHO ! 9
Geometry[angstrom] 13
C -1.20891 -0.05341 -0.22187
C -1.20880 -0.05305 1.15218

C -0.00790 -0.05106 1.86763
C 1.22998 0.03309 1.10886
C 1.21531 0.00599 -0.30485
C -0.00920 0.00780 -1.06055
O -0.06819 0.03260 -2.29232
H 2.15237 0.01854 -0.84859
H -2.15274 -0.06896 -0.75838
H -2.14734 -0.06502 1.69534
H 0.03117 -0.04643 2.94911
H 2.16122 -0.16564 1.63234
H 0.91264 1.12752 1.45098
Core RigidRotor
SymmetryFactor 0.50000000000000000
End
Frequencies[1/cm] 32

3233.8	3218.4	3204.7	3188.0	3174.3	2418.1	1707.9	1587.5	1526.1	1493.8
1423.9	1373.7	1282.8	1242.6	1182.0	1161.7	1076.8	1017.4	995.02	985.45
950.74	885.91	818.37	793.07	733.23	635.65	594.71	526.63	490.51	444.72
378.68	129.64								

ZeroEnergy[kcal/mol] 62.2004309277714937
ElectronicLevels[1/cm] 1
0.0000000000000000 1.0000000000000000
End
!***
RRHO ! 10
Geometry[angstrom] 13
C -1.20862 -0.05339 -0.22204
C -1.20851 -0.05321 1.15274
C -0.00867 -0.05225 1.86767
C 1.22959 0.03440 1.10979
C 1.21499 0.00589 -0.30521
C -0.00923 0.00782 -1.06069
O -0.06818 0.03258 -2.29249
H 2.15213 0.01877 -0.84880
H -2.15254 -0.06875 -0.75841
H -2.14737 -0.06482 1.69542
H 0.03017 -0.04530 2.94922
H 2.16047 -0.16900 1.63226
H 0.92560 1.13052 1.44367
Core RigidRotor
SymmetryFactor 0.50000000000000000
End
Frequencies[1/cm] 32

3233.2	3218.4	3204.5	3187.9	3173.2	2442.1	1707.8	1585.6	1527.2	1492.2
1423.8	1373.7	1282.8	1245.3	1181.7	1162.2	1076.9	1017.7	994.10	985.38
948.55	886.43	818.69	789.19	731.26	633.89	594.59	526.57	489.54	443.98
376.93	128.02								

ZeroEnergy[kcal/mol] 62.2426854417204836

```
 ElectronicLevels[1/cm]             1
  0.0000000000000000       1.0000000000000000
End
!*******************************************
RRHO          !          11
Geometry[angstrom]         13
C    0.00000   0.00000   0.00000
C    0.00000   0.00000   1.37545
C    1.19887   0.00000   2.08993
C    2.43754   0.08908   1.33291
C    2.42299   0.05914  -0.08340
C    1.19901   0.06124  -0.83865
O    1.14010   0.08594  -2.07054
H    3.36028   0.07242  -0.62687
H   -0.94402  -0.01518  -0.53630
H   -0.93917  -0.01129   1.91760
H    1.23743   0.00909   3.17146
H    3.36807  -0.11915   1.85440
H    2.14692   1.18685   1.65861
          Core      RigidRotor
        SymmetryFactor   0.50000000000000000
End
   Frequencies[1/cm]         32
  3232.9     3218.2     3204.3     3187.7     3171.8     2465.6     1707.3     1583.6     1528.4     1490.4
  1423.6     1373.8     1282.9     1248.1     1181.4     1162.6     1077.0     1018.0     993.24     985.31
  946.68     886.92     819.02     785.31     729.14     632.17     594.47     526.52     488.62     443.33
  375.18     126.55
ZeroEnergy[kcal/mol]    62.2585386669698706
 ElectronicLevels[1/cm]             1
  0.0000000000000000       1.0000000000000000
End
!*******************************************
RRHO          !          12
Geometry[angstrom]         13
C   -1.20796  -0.05337  -0.22238
C   -1.20807  -0.05355   1.15384
C   -0.01021  -0.05451   1.86783
C    1.22885   0.03701   1.11169
C    1.21437   0.00564  -0.30592
C   -0.00931   0.00790  -1.06090
O   -0.06819   0.03255  -2.29299
H    2.15173   0.01930  -0.84925
H   -2.15203  -0.06836  -0.75858
H   -2.14758  -0.06451   1.69548
H    0.02807  -0.04326   2.94943
H    2.15898  -0.17603   1.63217
H    0.95167   1.13647   1.42919
          Core      RigidRotor
```

SymmetryFactor 0.50000000000000000
End
Frequencies[1/cm] 32

3232.5	3217.9	3204.1	3187.5	3170.3	2488.8	1706.8	1581.7	1529.7	1488.5
1423.4	1373.8	1283.0	1250.9	1181.2	1162.8	1077.1	1018.4	992.47	985.26
945.18	887.33	819.35	781.48	726.90	630.45	594.36	526.49	487.63	442.69
373.42	125.07								

ZeroEnergy[kcal/mol] 62.2614700143583959
ElectronicLevels[1/cm] 1
0.0000000000000000 1.0000000000000000
End
!**
RRHO ! 13
Geometry[angstrom] 13
C -1.20758 -0.05337 -0.22254
C -1.20791 -0.05375 1.15439
C -0.01097 -0.05557 1.86794
C 1.22851 0.03831 1.11267
C 1.21408 0.00550 -0.30627
C -0.00938 0.00795 -1.06098
O -0.06820 0.03254 -2.29331
H 2.15158 0.01961 -0.84950
H -2.15174 -0.06817 -0.75872
H -2.14774 -0.06440 1.69547
H 0.02699 -0.04236 2.94954
H 2.15823 -0.17969 1.63216
H 0.96474 1.13942 1.42202
Core RigidRotor
SymmetryFactor 0.50000000000000000
End
Frequencies[1/cm] 32

3232.3	3217.3	3203.8	3187.3	3168.4	2511.7	1705.8	1579.8	1530.9	1486.5
1423.2	1373.9	1283.3	1253.8	1180.9	1163.0	1077.1	1018.7	991.73	985.20
944.01	887.76	819.68	777.78	724.48	628.79	594.24	526.48	486.73	442.15
371.67	123.77								

ZeroEnergy[kcal/mol] 62.2447469263253921
ElectronicLevels[1/cm] 1
0.0000000000000000 1.0000000000000000
End
!**
RRHO ! 14
Geometry[angstrom] 13
C -1.20724 -0.05335 -0.22270
C -1.20770 -0.05395 1.15495
C -0.01172 -0.05663 1.86803
C 1.22815 0.03962 1.11363
C 1.21376 0.00536 -0.30661
C -0.00943 0.00798 -1.06110

```
O   -0.06820   0.03252  -2.29357
H    2.15138   0.01995  -0.84972
H   -2.15147  -0.06796  -0.75882
H   -2.14787  -0.06431   1.69548
H    0.02589  -0.04153   2.94966
H    2.15740  -0.18342   1.63209
H    0.97784   1.14238   1.41490
          Core      RigidRotor
       SymmetryFactor   0.50000000000000000
End
   Frequencies[1/cm]         32
  3231.9     3217.1     3203.6     3187.1     3166.8     2533.9     1705.3     1578.0     1532.2     1484.6
  1423.0     1374.1     1283.5     1256.5     1180.7     1163.1     1077.1     1019.0     991.04     985.17
  943.17     888.08     819.98     774.32     721.87     627.10     594.12     526.47     485.65     441.52
  369.90     122.30
ZeroEnergy[kcal/mol]    62.2091270758920074
 ElectronicLevels[1/cm]              1
  0.0000000000000000       1.0000000000000000
End
!*******************************************
RRHO          !          15
Geometry[angstrom]         13
C   -1.20690  -0.05334  -0.22287
C   -1.20749  -0.05416   1.15550
C   -0.01246  -0.05766   1.86813
C    1.22778   0.04094   1.11458
C    1.21344   0.00522  -0.30694
C   -0.00948   0.00801  -1.06124
O   -0.06820   0.03251  -2.29384
H    2.15118   0.02033  -0.84994
H   -2.15121  -0.06775  -0.75891
H   -2.14799  -0.06425   1.69548
H    0.02477  -0.04078   2.94979
H    2.15652  -0.18724   1.63200
H    0.99097   1.14533   1.40783
          Core      RigidRotor
       SymmetryFactor   0.50000000000000000
End
   Frequencies[1/cm]         32
  3231.5     3216.8     3203.4     3186.9     3165.2     2555.1     1704.8     1576.2     1533.5     1482.6
  1422.8     1374.2     1283.7     1259.3     1180.6     1163.2     1077.0     1019.4     990.39     985.12
  942.61     888.36     820.26     771.18     719.03     625.44     593.98     526.48     484.53     440.89
  368.14     120.81
ZeroEnergy[kcal/mol]    62.1599849274677410
 ElectronicLevels[1/cm]              1
  0.0000000000000000       1.0000000000000000
End
!*******************************************
```

```
RRHO          !          16
Geometry[angstrom]         13
C   -1.20655  -0.05332  -0.22304
C   -1.20729  -0.05439   1.15605
C   -0.01319  -0.05867   1.86824
C    1.22742   0.04226   1.11554
C    1.21311   0.00508  -0.30727
C   -0.00954   0.00803  -1.06138
O   -0.06820   0.03250  -2.29412
H    2.15096   0.02074  -0.85014
H   -2.15093  -0.06752  -0.75900
H   -2.14812  -0.06422   1.69548
H    0.02363  -0.04011   2.94992
H    2.15561  -0.19115   1.63191
H    1.00413   1.14824   1.40083
           Core      RigidRotor
        SymmetryFactor   0.50000000000000000
End
   Frequencies[1/cm]         32
  3231.2     3216.4     3203.2     3186.8     3163.5     2575.2     1704.3     1574.5     1534.9     1480.5
  1422.5     1374.4     1284.0     1261.9     1180.4     1163.1     1077.0     1019.7     989.77     985.08
  942.27     888.59     820.52     768.40     715.87     623.81     593.85     526.50     483.41     440.28
  366.38     119.30
ZeroEnergy[kcal/mol]    62.1032953655921047
 ElectronicLevels[1/cm]                  1
  0.0000000000000000       1.0000000000000000
End
!********************************************
RRHO          !          17
Geometry[angstrom]         13
C   -1.20620  -0.05329  -0.22320
C   -1.20708  -0.05463   1.15661
C   -0.01391  -0.05965   1.86835
C    1.22706   0.04359   1.11650
C    1.21277   0.00493  -0.30760
C   -0.00961   0.00805  -1.06153
O   -0.06820   0.03248  -2.29440
H    2.15074   0.02118  -0.85035
H   -2.15066  -0.06729  -0.75910
H   -2.14825  -0.06422   1.69548
H    0.02248  -0.03953   2.95005
H    2.15465  -0.19516   1.63179
H    1.01732   1.15111   1.39389
           Core      RigidRotor
        SymmetryFactor   0.50000000000000000
End
   Frequencies[1/cm]         32
  3230.8     3216.1     3203.0     3186.6     3161.8     2594.3     1703.8     1572.8     1536.3     1478.4
```

1422.3 1374.6 1284.3 1264.5 1180.3 1163.0 1076.9 1020.0 989.17 985.02
942.12 888.78 820.78 766.06 712.35 622.23 593.71 526.54 482.29 439.66
364.64 117.77
ZeroEnergy[kcal/mol] 62.0265367924909241
ElectronicLevels[1/cm] 1
0.0000000000000000 1.0000000000000000
End
!***
RRHO ! 18
Geometry[angstrom] 13
C -1.20585 -0.05326 -0.22337
C -1.20688 -0.05488 1.15716
C -0.01462 -0.06059 1.86847
C 1.22670 0.04492 1.11745
C 1.21243 0.00478 -0.30791
C -0.00968 0.00807 -1.06168
O -0.06819 0.03247 -2.29469
H 2.15052 0.02166 -0.85054
H -2.15038 -0.06706 -0.75919
H -2.14839 -0.06426 1.69547
H 0.02132 -0.03903 2.95019
H 2.15365 -0.19927 1.63166
H 1.03054 1.15393 1.38701
Core RigidRotor
SymmetryFactor 0.50000000000000000
End
Frequencies[1/cm] 32
3230.4 3215.8 3202.8 3186.4 3160.1 2612.6 1703.4 1571.2 1537.6 1476.3
1422.0 1374.8 1284.7 1266.9 1180.3 1162.8 1076.7 1020.3 988.60 984.94
942.14 888.94 821.01 764.18 708.53 620.73 593.56 526.58 481.20 439.05
362.92 116.23
ZeroEnergy[kcal/mol] 61.9427597474814831
ElectronicLevels[1/cm] 1
0.0000000000000000 1.0000000000000000
End
!***
RRHO ! 19
Geometry[angstrom] 13
C -1.20549 -0.05323 -0.22354
C -1.20668 -0.05515 1.15770
C -0.01532 -0.06150 1.86860
C 1.22635 0.04627 1.11840
C 1.21208 0.00462 -0.30822
C -0.00976 0.00808 -1.06185
O -0.06819 0.03245 -2.29498
H 2.15028 0.02218 -0.85073
H -2.15010 -0.06681 -0.75927
H -2.14853 -0.06434 1.69546

H 0.02014 -0.03862 2.95034
H 2.15260 -0.20348 1.63151
H 1.04379 1.15669 1.38020
Core RigidRotor
SymmetryFactor 0.50000000000000000
End
Frequencies[1/cm] 32

3230.0	3215.4	3202.6	3186.3	3158.3	2630.1	1702.9	1569.8	1538.9	1474.2
1421.8	1375.0	1285.2	1269.2	1180.2	1162.6	1076.6	1020.6	988.07	984.83
942.31	889.10	821.23	762.77	704.53	619.34	593.42	526.65	480.16	438.45
361.26	114.71								

ZeroEnergy[kcal/mol] 61.8525646506937234
ElectronicLevels[1/cm] 1
0.0000000000000000 1.0000000000000000
End
!**
RRHO ! 20
Geometry[angstrom] 13
C -1.20513 -0.05319 -0.22370
C -1.20649 -0.05543 1.15825
C -0.01600 -0.06238 1.86874
C 1.22599 0.04761 1.11935
C 1.21172 0.00447 -0.30852
C -0.00985 0.00809 -1.06202
O -0.06818 0.03243 -2.29529
H 2.15004 0.02273 -0.85091
H -2.14982 -0.06656 -0.75936
H -2.14867 -0.06446 1.69544
H 0.01894 -0.03830 2.95051
H 2.15150 -0.20780 1.63135
H 1.05708 1.15938 1.37348
Core RigidRotor
SymmetryFactor 0.50000000000000000
End
Frequencies[1/cm] 32

3229.6	3215.0	3202.4	3186.1	3156.5	2646.9	1702.5	1568.4	1540.2	1472.0
1421.5	1375.2	1285.8	1271.3	1180.2	1162.4	1076.4	1020.9	987.57	984.69
942.61	889.27	821.44	761.82	700.47	618.06	593.27	526.73	479.22	437.84
359.67	113.22								

ZeroEnergy[kcal/mol] 61.7560086849971852
ElectronicLevels[1/cm] 1
0.0000000000000000 1.0000000000000000
End
!**
RRHO ! 21
Geometry[angstrom] 13
C -1.20477 -0.05316 -0.22387
C -1.20629 -0.05573 1.15879

```
C   -0.01667  -0.06322   1.86889
C    1.22565   0.04897   1.12030
C    1.21136   0.00431  -0.30882
C   -0.00994   0.00810  -1.06221
O   -0.06817   0.03241  -2.29560
H    2.14980   0.02332  -0.85108
H   -2.14954  -0.06629  -0.75945
H   -2.14882  -0.06463   1.69542
H    0.01774  -0.03807   2.95067
H    2.15034  -0.21224   1.63116
H    1.07041   1.16200   1.36684
         Core      RigidRotor
       SymmetryFactor   0.50000000000000000
End
   Frequencies[1/cm]          32
  3229.2     3214.6     3202.2     3185.9     3154.7     2662.8     1702.0     1567.2     1541.3     1469.9
  1421.2     1375.3     1286.5     1273.3     1180.2     1162.1     1076.3     1021.2     987.11     984.51
  943.00     889.45     821.63     761.33     696.43     616.91     593.12     526.84     478.40     437.25
  358.18     111.75
ZeroEnergy[kcal/mol]    61.6532919904352084
 ElectronicLevels[1/cm]                  1
  0.0000000000000000       1.0000000000000000
End
!*******************************************
    Tunneling    Eckart
    ImaginaryFrequency[1/cm]    822.86770000000001
    WellDepth[kcal/mol]          5.13
    WellDepth[kcal/mol]         45.87
End
End
Barrier TS10 W5 W4
 Variational
 RRHO          !          1
 Geometry[angstrom]          13
C   -1.36593   0.10904  -0.03962
C   -1.39636   0.13461   1.41938
C   -0.10350   0.13008   1.90776
C    0.85143  -0.26774   0.97343
C    0.14398  -0.71093  -0.29466
C    0.77463  -0.04340  -1.33455
O    1.04449   0.57300  -2.26574
H   -1.15023   1.08884  -0.48656
H   -2.19990  -0.36094  -0.56317
H   -2.28337  -0.05024   2.00818
H    0.15437   0.28554   2.94988
H    1.87484  -0.53757   1.17945
H    0.07441  -1.79996  -0.46456
         Core      RigidRotor
```

SymmetryFactor 0.50000000000000000
End
Frequencies[1/cm] 32

3264.3	3240.2	3198.1	3128.2	2990.6	2947.4	2158.0	1503.0	1485.3	1441.0
1317.4	1285.2	1265.2	1213.8	1112.8	1101.2	1076.0	1036.4	1009.0	954.02
928.22	895.50	817.46	644.55	574.57	571.55	510.28	468.18	341.47	327.92
173.41	89.670								

ZeroEnergy[kcal/mol] 91.798637571641400
ElectronicLevels[1/cm] 1
0.0000000000000000 1.0000000000000000
End
!***
RRHO ! 2
Geometry[angstrom] 13
C -1.36983 0.11169 -0.03760
C -1.39753 0.13497 1.41882
C -0.10299 0.13079 1.90745
C 0.85070 -0.26906 0.97535
C 0.14839 -0.71137 -0.29576
C 0.77517 -0.04577 -1.33643
O 1.04426 0.57363 -2.26598
H -1.14775 1.08865 -0.48654
H -2.20404 -0.35732 -0.56164
H -2.28142 -0.06214 2.00855
H 0.15490 0.28669 2.94953
H 1.87515 -0.53500 1.18169
H 0.08089 -1.80024 -0.46555
Core RigidRotor
SymmetryFactor 0.50000000000000000
End
Frequencies[1/cm] 32

3263.6	3238.8	3197.8	3128.8	2994.8	2952.9	2159.0	1501.5	1485.6	1443.6
1318.5	1285.3	1263.7	1211.8	1113.8	1101.0	1071.7	1039.1	1010.8	953.65
928.79	893.79	815.56	644.81	577.45	568.68	502.50	469.69	345.17	326.13
171.44	85.090								

ZeroEnergy[kcal/mol] 92.048539860257250
ElectronicLevels[1/cm] 1
0.0000000000000000 1.0000000000000000
End
!***
RRHO ! 3
Geometry[angstrom] 13
C -1.37380 0.11437 -0.03557
C -1.39872 0.13530 1.41824
C -0.10246 0.13149 1.90714
C 0.84997 -0.27033 0.97720
C 0.15288 -0.71190 -0.29687
C 0.77569 -0.04807 -1.33823

O 1.04405 0.57424 -2.26622
H -1.14520 1.08839 -0.48652
H -2.20823 -0.35367 -0.56009
H -2.27940 -0.07380 2.00885
H 0.15546 0.28789 2.94917
H 1.87539 -0.53253 1.18393
H 0.08719 -1.80057 -0.46652
Core RigidRotor
SymmetryFactor 0.50000000000000000
End
Frequencies[1/cm] 32

3262.8	3237.3	3197.4	3129.6	2999.1	2958.5	2160.0	1500.9	1485.5	1445.8
1319.6	1285.3	1262.5	1209.9	1115.0	1100.8	1067.5	1041.5	1012.5	953.33
929.45	892.17	813.65	645.54	580.89	565.52	496.03	471.26	348.96	324.35
169.65	81.410								

ZeroEnergy[kcal/mol] 92.279017170255202
ElectronicLevels[1/cm] 1
0.0000000000000000 1.0000000000000000
End
!***
RRHO ! 4
Geometry[angstrom] 13
C -1.37784 0.11708 -0.03351
C -1.39990 0.13560 1.41763
C -0.10192 0.13218 1.90682
C 0.84924 -0.27157 0.97898
C 0.15744 -0.71251 -0.29799
C 0.77619 -0.05030 -1.33996
O 1.04385 0.57482 -2.26644
H -1.14261 1.08804 -0.48649
H -2.21248 -0.34999 -0.55854
H -2.27732 -0.08524 2.00907
H 0.15605 0.28915 2.94878
H 1.87559 -0.53015 1.18618
H 0.09332 -1.80096 -0.46746
Core RigidRotor
SymmetryFactor 0.50000000000000000
End
Frequencies[1/cm] 32

3261.8	3235.7	3197.1	3130.5	3003.5	2964.4	2161.1	1501.3	1485.0	1447.8
1320.8	1285.5	1261.3	1208.0	1116.4	1100.5	1063.7	1043.1	1014.0	953.08
930.18	890.61	811.72	646.70	584.54	562.38	491.16	472.81	352.71	322.56
168.02	78.670								

ZeroEnergy[kcal/mol] 92.496358891957065
ElectronicLevels[1/cm] 1
0.0000000000000000 1.0000000000000000
End
!***

RRHO ! 5
Geometry[angstrom] 13
C -1.38195 0.11981 -0.03143
C -1.40108 0.13587 1.41701
C -0.10135 0.13287 1.90650
C 0.84851 -0.27277 0.98070
C 0.16206 -0.71320 -0.29912
C 0.77667 -0.05245 -1.34161
O 1.04367 0.57539 -2.26666
H -1.13998 1.08763 -0.48647
H -2.21678 -0.34630 -0.55696
H -2.27517 -0.09644 2.00922
H 0.15666 0.29047 2.94838
H 1.87573 -0.52787 1.18843
H 0.09926 -1.80139 -0.46837
Core RigidRotor
SymmetryFactor 0.50000000000000000
End
Frequencies[1/cm] 32
3260.7 3234.0 3196.7 3131.4 3008.0 2970.3 2162.2 1502.4 1484.5 1449.4
1322.0 1285.7 1260.4 1206.1 1117.9 1100.1 1061.1 1043.3 1015.3 952.89
930.98 889.09 809.75 648.22 588.31 559.27 488.04 474.31 356.36 320.76
166.50 76.810
ZeroEnergy[kcal/mol] 92.694004016410801
ElectronicLevels[1/cm] 1
0.0000000000000000 1.0000000000000000
End
!**
RRHO ! 6
Geometry[angstrom] 13
C -1.38612 0.12258 -0.02934
C -1.40225 0.13611 1.41637
C -0.10078 0.13356 1.90618
C 0.84778 -0.27394 0.98236
C 0.16674 -0.71397 -0.30025
C 0.77713 -0.05452 -1.34320
O 1.04350 0.57593 -2.26688
H -1.13730 1.08713 -0.48644
H -2.22112 -0.34258 -0.55537
H -2.27298 -0.10741 2.00930
H 0.15729 0.29184 2.94796
H 1.87583 -0.52568 1.19069
H 0.10501 -1.80188 -0.46926
Core RigidRotor
SymmetryFactor 0.50000000000000000
End
Frequencies[1/cm] 32
3259.6 3232.3 3196.4 3132.5 3012.5 2976.4 2163.4 1504.2 1484.1 1450.4

1323.4 1286.1 1259.6 1204.2 1119.5 1099.8 1060.5 1041.7 1016.2 952.78
931.86 887.58 807.75 650.04 592.18 556.22 486.59 475.83 359.84 318.94
165.03 75.700
ZeroEnergy[kcal/mol] 92.865320056077373
ElectronicLevels[1/cm] 1
0.0000000000000000 1.0000000000000000
End
!***
RRHO ! 7
Geometry[angstrom] 13
C -1.39035 0.12536 -0.02723
C -1.40342 0.13632 1.41571
C -0.10019 0.13423 1.90586
C 0.84705 -0.27508 0.98395
C 0.17148 -0.71481 -0.30139
C 0.77757 -0.05653 -1.34473
O 1.04334 0.57645 -2.26709
H -1.13460 1.08656 -0.48641
H -2.22551 -0.33884 -0.55377
H -2.27074 -0.11815 2.00931
H 0.15793 0.29325 2.94753
H 1.87588 -0.52358 1.19294
H 0.11056 -1.80242 -0.47012
Core RigidRotor
SymmetryFactor 0.50000000000000000
End
Frequencies[1/cm] 32
3258.3 3230.5 3196.0 3133.6 3017.2 2982.5 2164.8 1506.5 1484.0 1451.0
1324.8 1286.7 1259.0 1202.4 1121.1 1099.4 1061.5 1038.6 1016.5 952.73
932.81 886.04 805.72 652.12 596.11 553.21 486.46 477.60 363.18 317.08
163.58 75.240
ZeroEnergy[kcal/mol] 93.022213892883663
ElectronicLevels[1/cm] 1
0.0000000000000000 1.0000000000000000
End
!***
RRHO ! 8
Geometry[angstrom] 13
C -1.39464 0.12818 -0.02510
C -1.40458 0.13649 1.41504
C -0.09959 0.13490 1.90553
C 0.84633 -0.27619 0.98548
C 0.17627 -0.71572 -0.30251
C 0.77800 -0.05846 -1.34619
O 1.04319 0.57695 -2.26730
H -1.13186 1.08592 -0.48638
H -2.22995 -0.33508 -0.55214
H -2.26846 -0.12866 2.00926

H 0.15860 0.29471 2.94709
H 1.87589 -0.52157 1.19520
H 0.11592 -1.80300 -0.47096
Core RigidRotor
SymmetryFactor 0.50000000000000000
End
Frequencies[1/cm] 32

3257.0	3228.6	3195.7	3134.8	3021.8	2988.7	2166.2	1509.1	1484.3	1451.1
1326.3	1287.4	1258.5	1200.5	1122.8	1098.9	1063.4	1035.2	1016.1	952.77
933.83	884.46	803.65	654.44	600.07	550.23	487.20	479.86	366.38	315.20
162.11	75.310								

ZeroEnergy[kcal/mol] 93.151606396077568
ElectronicLevels[1/cm] 1
0.0000000000000000 1.0000000000000000
End
!***
RRHO ! 9
Geometry[angstrom] 13
C -1.39899 0.13103 -0.02297
C -1.40573 0.13664 1.41436
C -0.09897 0.13556 1.90520
C 0.84562 -0.27727 0.98695
C 0.18110 -0.71670 -0.30364
C 0.77840 -0.06033 -1.34760
O 1.04305 0.57743 -2.26750
H -1.12912 1.08520 -0.48635
H -2.23443 -0.33131 -0.55050
H -2.26614 -0.13893 2.00915
H 0.15928 0.29619 2.94664
H 1.87587 -0.51965 1.19746
H 0.12107 -1.80362 -0.47178
Core RigidRotor
SymmetryFactor 0.50000000000000000
End
Frequencies[1/cm] 32

3255.6	3226.7	3195.4	3136.2	3026.6	2995.0	2167.6	1512.0	1485.1	1450.7
1327.8	1288.2	1258.1	1198.8	1124.5	1098.5	1065.8	1032.5	1014.4	952.88
934.90	882.78	801.53	656.99	604.01	547.28	488.51	482.67	369.48	313.29
160.62	75.840								

ZeroEnergy[kcal/mol] 93.252797075507480
ElectronicLevels[1/cm] 1
0.0000000000000000 1.0000000000000000
End
!***
RRHO ! 10
Geometry[angstrom] 13
C -1.40347 0.13392 -0.02082
C -1.40679 0.13677 1.41365

C -0.09833 0.13616 1.90488
C 0.84494 -0.27834 0.98832
C 0.18595 -0.71771 -0.30476
C 0.77878 -0.06212 -1.34891
O 1.04292 0.57789 -2.26771
H -1.12643 1.08442 -0.48635
H -2.23900 -0.32753 -0.54876
H -2.26377 -0.14891 2.00900
H 0.15998 0.29765 2.94622
H 1.87585 -0.51783 1.19966
H 0.12603 -1.80423 -0.47256
Core RigidRotor
SymmetryFactor 0.50000000000000000
End
Frequencies[1/cm] 32
3254.2 3224.3 3194.7 3137.9 3031.4 3001.5 2169.0 1515.2 1486.3 1450.1
1329.5 1289.3 1257.8 1197.0 1126.2 1098.1 1068.3 1030.7 1011.2 953.20
936.06 880.89 799.34 659.79 608.11 544.40 490.42 485.81 372.53 311.33
159.19 76.770
ZeroEnergy[kcal/mol] 93.326057549803622
ElectronicLevels[1/cm] 1
0.0000000000000000 1.0000000000000000
End
!**
RRHO ! 11
Geometry[angstrom] 13
C 0.00000 0.00000 0.00000
C 0.00000 0.00000 1.43163
C 1.31021 0.00000 1.92325
C 2.25216 -0.41615 1.00838
C 1.59883 -0.85567 -0.28720
C 2.18711 -0.20064 -1.33151
O 2.45077 0.44150 -2.24920
H 0.28432 0.94669 -0.46764
H -0.83566 -0.46054 -0.52846
H -0.85347 -0.29542 2.02740
H 1.56858 0.16245 2.96440
H 3.28367 -0.65288 1.22067
H 1.53863 -1.94183 -0.45464
Core RigidRotor
SymmetryFactor 0.50000000000000000
End
Frequencies[1/cm] 32
3252.7 3222.7 3194.4 3139.5 3036.5 3007.9 2170.7 1518.6 1487.8 1449.1
1331.2 1290.5 1257.4 1195.3 1127.9 1097.6 1071.0 1029.7 1007.0 953.44
937.20 878.89 797.10 662.68 611.83 541.45 493.17 488.55 375.48 309.37
157.65 77.980
ZeroEnergy[kcal/mol] 93.375775878876304

ElectronicLevels[1/cm] 1
0.0000000000000000 1.0000000000000000
End
!***
RRHO ! 12
Geometry[angstrom] 13
C -1.41238 0.13972 -0.01653
C -1.40905 0.13687 1.41221
C -0.09710 0.13748 1.90422
C 0.84356 -0.28034 0.99101
C 0.19586 -0.71999 -0.30697
C 0.77953 -0.06554 -1.35146
O 1.04269 0.57877 -2.26811
H -1.12079 1.08260 -0.48627
H -2.24811 -0.31989 -0.54547
H -2.25908 -0.16833 2.00845
H 0.16134 0.30089 2.94526
H 1.87566 -0.51429 1.20423
H 0.13535 -1.80571 -0.47404
Core RigidRotor
SymmetryFactor 0.50000000000000000
End
Frequencies[1/cm] 32

3251.1	3220.3	3194.1	3141.1	3041.1	3014.1	2172.3	1522.2	1489.6	1448.0
1332.9	1292.0	1257.3	1193.7	1129.5	1097.1	1073.6	1029.1	1002.4	953.76
938.35	876.78	794.78	665.83	615.84	538.63	497.17	490.60	378.30	307.43
156.09	79.580								

ZeroEnergy[kcal/mol] 93.395519715229746
ElectronicLevels[1/cm] 1
0.0000000000000000 1.0000000000000000
End
!***
RRHO ! 13
Geometry[angstrom] 13
C -1.41681 0.14260 -0.01440
C -1.41026 0.13685 1.41148
C -0.09652 0.13821 1.90390
C 0.84285 -0.28126 0.99232
C 0.20092 -0.72126 -0.30807
C 0.77990 -0.06715 -1.35268
O 1.04259 0.57918 -2.26831
H -1.11788 1.08157 -0.48620
H -2.25265 -0.31606 -0.54389
H -2.25677 -0.17774 2.00804
H 0.16200 0.30266 2.94471
H 1.87549 -0.51257 1.20661
H 0.13973 -1.80658 -0.47474
Core RigidRotor

SymmetryFactor 0.50000000000000000
End
Frequencies[1/cm] 32
3249.6 3218.9 3194.1 3142.5 3046.0 3020.1 2174.3 1526.0 1491.5 1446.8
1334.6 1293.6 1257.1 1192.1 1131.2 1096.4 1076.3 1028.7 997.58 954.01
939.45 874.50 792.39 669.06 619.46 535.80 501.64 492.14 381.02 305.48
154.39 81.290
ZeroEnergy[kcal/mol] 93.378299178390549
ElectronicLevels[1/cm] 1
0.0000000000000000 1.0000000000000000
End
!***
RRHO ! 14
Geometry[angstrom] 13
C -1.42136 0.14554 -0.01226
C -1.41138 0.13681 1.41073
C -0.09592 0.13888 1.90357
C 0.84218 -0.28219 0.99355
C 0.20597 -0.72255 -0.30915
C 0.78025 -0.06871 -1.35384
O 1.04250 0.57958 -2.26851
H -1.11502 1.08047 -0.48615
H -2.25726 -0.31222 -0.54224
H -2.25442 -0.18691 2.00762
H 0.16267 0.30441 2.94421
H 1.87533 -0.51092 1.20893
H 0.14392 -1.80742 -0.47541
Core RigidRotor
SymmetryFactor 0.50000000000000000
End
Frequencies[1/cm] 32
3247.9 3217.0 3193.8 3144.4 3051.0 3026.4 2176.1 1529.8 1493.6 1445.6
1336.4 1295.5 1256.9 1190.6 1132.8 1095.9 1078.8 1028.1 992.82 954.46
940.58 871.91 789.87 672.43 623.24 533.15 506.38 493.43 383.67 303.53
152.69 83.080
ZeroEnergy[kcal/mol] 93.331061261680013
ElectronicLevels[1/cm] 1
0.0000000000000000 1.0000000000000000
End
!***
RRHO ! 15
Geometry[angstrom] 13
C -1.42594 0.14850 -0.01012
C -1.41248 0.13675 1.40998
C -0.09532 0.13954 1.90324
C 0.84153 -0.28309 0.99473
C 0.21106 -0.72389 -0.31022
C 0.78060 -0.07022 -1.35495

O 1.04241 0.57997 -2.26871
H -1.11218 1.07930 -0.48610
H -2.26192 -0.30836 -0.54057
H -2.25208 -0.19584 2.00715
H 0.16335 0.30617 2.94369
H 1.87515 -0.50934 1.21124
H 0.14792 -1.80830 -0.47607
Core RigidRotor
SymmetryFactor 0.50000000000000000
End
Frequencies[1/cm] 32
3246.3 3215.2 3193.5 3146.4 3056.0 3032.6 2178.0 1533.7 1495.9 1444.5
1338.3 1297.5 1256.8 1189.0 1134.4 1095.3 1081.4 1027.4 988.29 954.97
941.68 869.09 787.23 675.89 626.99 530.75 510.99 494.52 386.21 301.59
150.91 84.890
ZeroEnergy[kcal/mol] 93.252690899142486
ElectronicLevels[1/cm] 1
0.0000000000000000 1.0000000000000000
End
!**
RRHO ! 16
Geometry[angstrom] 13
C -1.43057 0.15148 -0.00798
C -1.41357 0.13665 1.40923
C -0.09471 0.14019 1.90292
C 0.84090 -0.28398 0.99587
C 0.21618 -0.72527 -0.31128
C 0.78093 -0.07167 -1.35602
O 1.04233 0.58034 -2.26892
H -1.10934 1.07806 -0.48606
H -2.26662 -0.30447 -0.53887
H -2.24973 -0.20456 2.00665
H 0.16403 0.30795 2.94316
H 1.87496 -0.50781 1.21356
H 0.15173 -1.80920 -0.47670
Core RigidRotor
SymmetryFactor 0.50000000000000000
End
Frequencies[1/cm] 32
3244.7 3213.4 3193.2 3148.5 3061.0 3038.7 2180.0 1537.7 1498.3 1443.4
1340.1 1299.7 1256.8 1187.4 1136.0 1094.7 1083.8 1026.6 984.11 955.54
942.76 866.07 784.45 679.39 630.72 528.89 515.08 495.43 388.61 299.67
149.09 86.660
ZeroEnergy[kcal/mol] 93.143145203625828
ElectronicLevels[1/cm] 1
0.0000000000000000 1.0000000000000000
End
!**

```
RRHO          !          17
Geometry[angstrom]         13
C  -1.43523  0.15448  -0.00585
C  -1.41464  0.13653  1.40848
C  -0.09410  0.14083  1.90259
C   0.84027  -0.28485  0.99696
C   0.22132  -0.72670  -0.31232
C   0.78126  -0.07307  -1.35705
O   1.04225  0.58070  -2.26912
H  -1.10652  1.07675  -0.48601
H  -2.27136  -0.30057  -0.53717
H  -2.24741  -0.21305  2.00611
H   0.16472  0.30977  2.94263
H   1.87475  -0.50633  1.21588
H   0.15535  -1.81014  -0.47732
           Core     RigidRotor
        SymmetryFactor   0.50000000000000000
End
   Frequencies[1/cm]         32
  3243.0     3211.7     3192.9     3150.7     3066.0     3044.8     2182.0     1541.8     1500.9     1442.5
  1341.9     1302.0     1256.8     1185.8     1137.6     1094.2     1086.2     1025.5     980.34     956.17
  943.81     862.86     781.52     682.92     634.38     528.35     517.76     496.15     390.86     297.78
  147.24     88.370
ZeroEnergy[kcal/mol]    93.001995303608588
 ElectronicLevels[1/cm]                1
  0.0000000000000000       1.0000000000000000
End
!*********************************************
RRHO          !          18
Geometry[angstrom]         13
C  -1.43992  0.15750  -0.00372
C  -1.41570  0.13637  1.40773
C  -0.09350  0.14147  1.90226
C   0.83967  -0.28569  0.99801
C   0.22649  -0.72818  -0.31335
C   0.78159  -0.07442  -1.35805
O   1.04218  0.58105  -2.26933
H  -1.10372  1.07538  -0.48596
H  -2.27613  -0.29665  -0.53544
H  -2.24510  -0.22132  2.00554
H   0.16539  0.31160  2.94208
H   1.87453  -0.50490  1.21820
H   0.15880  -1.81110  -0.47792
           Core     RigidRotor
        SymmetryFactor   0.50000000000000000
End
   Frequencies[1/cm]         32
  3241.4     3210.1     3192.5     3153.0     3070.9     3050.8     2184.0     1545.9     1503.5     1441.6
```

1343.8 1304.4 1256.9 1184.2 1139.1 1093.7 1088.3 1024.3 977.04 956.84
944.81 859.50 778.42 686.44 637.97 530.03 518.12 496.67 392.93 295.92
145.36 89.970
ZeroEnergy[kcal/mol] 92.822665894421384
ElectronicLevels[1/cm] 1
0.0000000000000000 1.0000000000000000
End
!***
RRHO ! 19
Geometry[angstrom] 13
C -1.44464 0.16053 -0.00160
C -1.41675 0.13618 1.40699
C -0.09291 0.14211 1.90193
C 0.83908 -0.28652 0.99903
C 0.23169 -0.72969 -0.31437
C 0.78191 -0.07573 -1.35902
O 1.04211 0.58138 -2.26953
H -1.10094 1.07394 -0.48592
H -2.28095 -0.29270 -0.53371
H -2.24281 -0.22939 2.00494
H 0.16607 0.31346 2.94153
H 1.87430 -0.50351 1.22052
H 0.16207 -1.81209 -0.47850
Core RigidRotor
SymmetryFactor 0.50000000000000000
End
Frequencies[1/cm] 32
3239.7 3208.4 3192.2 3155.4 3075.7 3056.7 2186.2 1550.0 1506.3 1440.9
1345.6 1307.0 1257.1 1182.6 1140.6 1093.6 1090.1 1022.9 974.22 957.57
945.77 856.06 775.15 689.92 641.48 533.12 516.94 496.99 394.79 294.10
143.44 91.430
ZeroEnergy[kcal/mol] 92.611360592081695
ElectronicLevels[1/cm] 1
0.0000000000000000 1.0000000000000000
End
!***
RRHO ! 20
Geometry[angstrom] 13
C -1.44939 0.16358 0.00051
C -1.41778 0.13596 1.40624
C -0.09232 0.14274 1.90160
C 0.83851 -0.28734 1.00000
C 0.23690 -0.73125 -0.31536
C 0.78224 -0.07699 -1.35995
O 1.04204 0.58171 -2.26974
H -1.09818 1.07244 -0.48588
H -2.28580 -0.28874 -0.53195
H -2.24055 -0.23724 2.00432

H 0.16674 0.31534 2.94097
H 1.87407 -0.50215 1.22284
H 0.16516 -1.81310 -0.47906
Core RigidRotor
SymmetryFactor 0.50000000000000000
End
Frequencies[1/cm] 32
3238.1 3206.9 3191.8 3157.9 3080.4 3062.5 2188.3 1554.1 1509.2 1440.3
1347.4 1309.6 1257.3 1180.9 1142.0 1094.3 1090.9 1021.4 971.91 958.32
946.67 852.58 771.71 693.35 644.90 536.54 515.31 497.06 396.41 292.33
141.47 92.730
ZeroEnergy[kcal/mol] 92.374125759715987
ElectronicLevels[1/cm] 1
0.0000000000000000 1.0000000000000000
End
!**
RRHO ! 21
Geometry[angstrom] 13
C -1.45417 0.16665 0.00261
C -1.41880 0.13571 1.40550
C -0.09174 0.14336 1.90127
C 0.83796 -0.28813 1.00094
C 0.24214 -0.73284 -0.31634
C 0.78256 -0.07822 -1.36086
O 1.04198 0.58203 -2.26995
H -1.09545 1.07087 -0.48584
H -2.29069 -0.28475 -0.53019
H -2.23832 -0.24488 2.00367
H 0.16740 0.31724 2.94040
H 1.87382 -0.50082 1.22517
H 0.16808 -1.81413 -0.47961
Core RigidRotor
SymmetryFactor 0.50000000000000000
End
Frequencies[1/cm] 32
3236.5 3205.4 3191.3 3160.4 3085.0 3068.2 2190.5 1558.3 1512.2 1439.9
1349.2 1312.3 1257.7 1179.2 1143.4 1095.8 1090.7 1019.8 970.09 959.09
947.53 849.15 768.08 696.70 648.21 539.87 513.68 496.89 397.75 290.60
139.46 93.840
ZeroEnergy[kcal/mol] 92.098354025245705
ElectronicLevels[1/cm] 1
0.0000000000000000 1.0000000000000000
End
!**
Tunneling Eckart
ImaginaryFrequency[1/cm] 600.22040000000004
WellDepth[kcal/mol] 51.45
WellDepth[kcal/mol] 42.85

```
 End
End
Barrier TS11 W1 Pr3
 Variational
 RRHO          !           1
 Geometry[angstrom]         13
 C   -1.27225   0.01102  -1.15738
 C   -1.27742   0.01060   0.23640
 C   -0.06048   0.01099  -1.85220
 H   -2.21218   0.02045  -1.69694
 C   -0.01738  -0.00643   0.78597
 H   -2.19278   0.03069   0.81460
 C    1.16767   0.01376  -1.18306
 H   -0.07969   0.01501  -2.93744
 C    1.22167   0.00209   0.22415
 O    0.24449   0.01442   2.27692
 H    2.07962   0.02406  -1.77342
 H    1.31836   0.02616   1.86518
 H    0.04257  -0.84574   2.67053
            Core      RigidRotor
         SymmetryFactor   0.50000000000000000
 End
    Frequencies[1/cm]         32
  3796.7     3219.7     3203.9     3187.2     3166.4     1936.3     1667.4     1601.1     1488.3     1454.4
  1360.1     1331.2     1282.0     1181.0     1140.3     1081.0     1054.5     1033.4     1014.1     995.68
  961.12     882.60     764.73     756.06     706.54     628.05     599.23     561.28     438.78     424.26
  408.05     232.90
 ZeroEnergy[kcal/mol]    85.134027802177920
  ElectronicLevels[1/cm]                 1
  0.0000000000000000       1.0000000000000000
 End
!********************************************
 RRHO          !           2
 Geometry[angstrom]         13
 C   -1.27232   0.01101  -1.15735
 C   -1.27730   0.01061   0.23649
 C   -0.06064   0.01099  -1.85245
 H   -2.21238   0.02044  -1.69668
 C   -0.01663  -0.00634   0.78464
 H   -2.19266   0.03076   0.81473
 C    1.16732   0.01374  -1.18347
 H   -0.08021   0.01504  -2.93770
 C    1.22213   0.00205   0.22334
 O    0.24455   0.01438   2.27778
 H    2.07935   0.02405  -1.77385
 H    1.30919   0.02672   1.88300
 H    0.04281  -0.84606   2.67132
            Core      RigidRotor
```

SymmetryFactor 0.50000000000000000
End
Frequencies[1/cm] 32

3794.1	3219.3	3203.7	3186.7	3165.4	1975.2	1668.9	1601.2	1488.6	1453.6
1375.6	1334.9	1283.5	1180.9	1140.2	1075.4	1053.3	1032.5	1014.2	994.32
960.51	882.49	766.06	755.34	705.44	628.25	604.90	559.09	438.07	423.41
408.51	229.16								

ZeroEnergy[kcal/mol] 85.492444529475671
ElectronicLevels[1/cm] 1
0.0000000000000000 1.0000000000000000
End
!***
RRHO ! 3
Geometry[angstrom] 13
C -1.27240 0.01100 -1.15731
C -1.27714 0.01063 0.23658
C -0.06082 0.01099 -1.85271
H -2.21260 0.02043 -1.69639
C -0.01580 -0.00626 0.78324
H -2.19249 0.03083 0.81491
C 1.16695 0.01371 -1.18393
H -0.08076 0.01507 -2.93799
C 1.22263 0.00202 0.22249
O 0.24450 0.01433 2.27875
H 2.07904 0.02403 -1.77436
H 1.30053 0.02741 1.90081
H 0.04292 -0.84639 2.67216
Core RigidRotor
SymmetryFactor 0.50000000000000000
End
Frequencies[1/cm] 32

3791.7	3219.0	3203.5	3186.3	3164.4	2020.7	1670.3	1601.3	1488.9	1452.8
1392.1	1337.2	1284.8	1180.8	1140.2	1069.7	1052.2	1031.2	1014.4	992.75
959.90	882.38	767.57	754.62	704.26	629.32	610.39	556.00	437.24	422.56
408.50	225.43								

ZeroEnergy[kcal/mol] 85.789211806292309
ElectronicLevels[1/cm] 1
0.0000000000000000 1.0000000000000000
End
!***
RRHO ! 4
Geometry[angstrom] 13
C -1.27249 0.01099 -1.15727
C -1.27694 0.01064 0.23668
C -0.06103 0.01100 -1.85301
H -2.21284 0.02042 -1.69607
C -0.01487 -0.00616 0.78179
H -2.19224 0.03092 0.81513

```
C   1.16657  0.01368 -1.18445
H  -0.08137  0.01509 -2.93831
C   1.22317  0.00198  0.22160
O   0.24433  0.01427  2.27986
H   2.07869  0.02402 -1.77496
H   1.29242  0.02825  1.91853
H   0.04289 -0.84675  2.67306
        Core     RigidRotor
      SymmetryFactor   0.50000000000000000
End
  Frequencies[1/cm]        32
 3789.6    3218.7    3203.3    3185.8    3163.4    2073.3    1671.6    1601.3    1489.2    1452.0
 1408.7    1339.1    1286.1    1180.7    1140.2    1064.0    1051.3    1029.8    1014.5    991.42
 959.31    882.25    772.01    753.93    703.08    632.32    615.43    552.03    436.25    421.72
 407.98    221.82
ZeroEnergy[kcal/mol]   86.044299299157325
 ElectronicLevels[1/cm]              1
 0.0000000000000000      1.0000000000000000
End
!*******************************************
RRHO         !         5
Geometry[angstrom]        13
C  -1.27260  0.01098 -1.15722
C  -1.27669  0.01066  0.23679
C  -0.06127  0.01100 -1.85333
H  -2.21312  0.02042 -1.69571
C  -0.01383 -0.00607  0.78028
H  -2.19190  0.03101  0.81541
C   1.16617  0.01364 -1.18503
H  -0.08202  0.01512 -2.93865
C   1.22376  0.00194  0.22068
O   0.24401  0.01420  2.28112
H   2.07831  0.02400 -1.77566
H   1.28493  0.02925  1.93609
H   0.04269 -0.84712  2.67402
        Core     RigidRotor
      SymmetryFactor   0.50000000000000000
End
  Frequencies[1/cm]        32
 3787.8    3218.5    3203.1    3185.4    3162.5    2132.5    1672.8    1601.2    1489.6    1451.2
 1425.0    1340.7    1287.3    1180.6    1140.2    1058.3    1050.4    1028.3    1014.6    989.80
 958.75    882.13    778.87    753.26    701.93    638.42    618.17    547.36    435.07    420.91
 406.97    218.41
ZeroEnergy[kcal/mol]   86.266840520825719
 ElectronicLevels[1/cm]              1
 0.0000000000000000      1.0000000000000000
End
!*******************************************
```

```
RRHO        !        6
Geometry[angstrom]        13
C   -1.27271  0.01096  -1.15716
C   -1.27636  0.01068   0.23689
C   -0.06155  0.01099  -1.85370
H   -2.21343  0.02041  -1.69530
C   -0.01268 -0.00597   0.77873
H   -2.19145  0.03111   0.81576
C    1.16577  0.01361  -1.18569
H   -0.08274  0.01515  -2.93903
C    1.22442  0.00191   0.21971
O    0.24354  0.01412   2.28252
H    2.07788  0.02398  -1.77649
H    1.27810  0.03041   1.95343
H    0.04230 -0.84753   2.67503
        Core     RigidRotor
      SymmetryFactor   0.50000000000000000
End
   Frequencies[1/cm]        32
 3786.4    3218.2    3202.9    3185.0    3161.6    2197.4    1673.9    1601.1    1489.9    1450.4
 1440.8    1342.0    1288.3    1180.5    1140.3    1052.6    1049.6    1026.5    1014.6    987.77
 958.22    881.99    786.31    752.63    700.80    645.81    618.81    542.21    433.72    420.18
 405.54    215.28
ZeroEnergy[kcal/mol]    86.456734675950310
 ElectronicLevels[1/cm]              1
 0.0000000000000000       1.0000000000000000
End
!*********************************************
RRHO        !        7
Geometry[angstrom]        13
C   -1.27284  0.01095  -1.15708
C   -1.27596  0.01071   0.23700
C   -0.06187  0.01099  -1.85410
H   -2.21378  0.02040  -1.69483
C   -0.01141 -0.00586   0.77714
H   -2.19087  0.03122   0.81619
C    1.16535  0.01357  -1.18644
H   -0.08352  0.01517  -2.93944
C    1.22513  0.00189   0.21871
O    0.24288  0.01403   2.28409
H    2.07739  0.02396  -1.77745
H    1.27193  0.03173   1.97035
H    0.04170 -0.84797   2.67609
        Core     RigidRotor
      SymmetryFactor   0.50000000000000000
End
   Frequencies[1/cm]        32
 3785.3    3218.0    3202.7    3184.7    3160.8    2266.6    1674.8    1600.9    1490.3    1456.0
```

1449.6 1343.3 1289.2 1180.5 1140.4 1049.5 1046.9 1024.8 1014.7 986.09
957.72 881.86 800.10 752.05 699.77 653.44 618.88 536.80 432.20 419.52
403.73 212.49
ZeroEnergy[kcal/mol] 86.636567547341210
ElectronicLevels[1/cm] 1
0.0000000000000000 1.0000000000000000
End
!**
RRHO ! 8
Geometry[angstrom] 13
C -1.27299 0.01093 -1.15699
C -1.27548 0.01073 0.23712
C -0.06223 0.01098 -1.85454
H -2.21417 0.02039 -1.69430
C -0.01002 -0.00575 0.77551
H -2.19016 0.03135 0.81669
C 1.16494 0.01353 -1.18727
H -0.08437 0.01519 -2.93989
C 1.22591 0.00186 0.21767
O 0.24206 0.01392 2.28582
H 2.07686 0.02394 -1.77855
H 1.26637 0.03320 1.98676
H 0.04089 -0.84845 2.67718
Core RigidRotor
SymmetryFactor 0.50000000000000000
End
Frequencies[1/cm] 32
3784.5 3217.8 3202.5 3184.4 3160.0 2337.4 1675.7 1600.6 1490.7 1470.2
1448.8 1344.5 1289.9 1180.4 1140.5 1049.0 1042.0 1022.8 1014.7 984.24
957.25 881.71 817.16 751.51 698.82 659.83 618.47 531.32 430.52 418.93
401.52 210.07
ZeroEnergy[kcal/mol] 86.794831082507097
ElectronicLevels[1/cm] 1
0.0000000000000000 1.0000000000000000
End
!**
RRHO ! 9
Geometry[angstrom] 13
C -1.27314 0.01091 -1.15689
C -1.27491 0.01077 0.23723
C -0.06263 0.01096 -1.85501
H -2.21460 0.02039 -1.69372
C -0.00854 -0.00564 0.77389
H -2.18933 0.03149 0.81727
C 1.16451 0.01349 -1.18818
H -0.08527 0.01520 -2.94036
C 1.22674 0.00185 0.21660
O 0.24108 0.01380 2.28766

H 2.07629 0.02392 -1.77978
H 1.26136 0.03480 2.00271
H 0.03987 -0.84895 2.67830
Core RigidRotor
SymmetryFactor 0.50000000000000000
End
Frequencies[1/cm] 32
3784.0 3217.6 3202.4 3184.2 3159.3 2407.8 1676.4 1600.3 1491.6 1482.6
1448.1 1345.4 1290.4 1180.3 1140.6 1048.6 1037.1 1020.8 1014.6 982.06
956.80 881.57 831.58 751.01 697.92 664.08 617.78 525.80 428.70 418.35
398.92 207.95
ZeroEnergy[kcal/mol] 86.929708274689611
ElectronicLevels[1/cm] 1
0.0000000000000000 1.0000000000000000
End
!**
RRHO ! 10
Geometry[angstrom] 13
C -1.27328 0.01089 -1.15677
C -1.27425 0.01083 0.23735
C -0.06308 0.01089 -1.85549
H -2.21504 0.02039 -1.69307
C -0.00696 -0.00555 0.77226
H -2.18835 0.03164 0.81791
C 1.16405 0.01348 -1.18914
H -0.08624 0.01521 -2.94082
C 1.22764 0.00187 0.21551
O 0.23997 0.01366 2.28958
H 2.07561 0.02406 -1.78110
H 1.25690 0.03618 2.01816
H 0.03811 -0.84922 2.67978
Core RigidRotor
SymmetryFactor 0.50000000000000000
End
Frequencies[1/cm] 32
3783.3 3217.4 3202.2 3184.1 3158.9 2475.9 1677.1 1600.1 1497.4 1488.8
1447.5 1346.3 1290.8 1180.2 1140.8 1048.4 1032.8 1018.7 1014.3 980.09
956.35 881.35 847.44 750.53 697.08 666.74 617.04 520.52 426.78 417.69
396.16 206.13
ZeroEnergy[kcal/mol] 87.041914635083311
ElectronicLevels[1/cm] 1
0.0000000000000000 1.0000000000000000
End
!**
RRHO ! 11
Geometry[angstrom] 13
C 0.00000 0.00000 0.00000
C 0.00000 0.00000 1.39411

```
C   1.20992  0.00000  -0.69944
H  -0.94211  0.00953  -0.53569
C   1.26819 -0.01630   1.92729
H  -0.91370  0.02093   1.97533
C   2.43716  0.00256  -0.03361
H   1.18620  0.00434  -1.78479
C   2.50208 -0.00899   1.37104
O   1.51209  0.00265   3.44839
H   3.34850  0.01316  -0.62606
H   2.52631  0.02718   3.18909
H   1.31026 -0.86071   3.83752
        Core     RigidRotor
      SymmetryFactor   0.50000000000000000
End
   Frequencies[1/cm]         32
  3783.4    3217.3    3202.1    3183.9    3158.3    2536.9    1677.7    1599.7    1507.2    1489.7
  1446.8    1346.9    1290.9    1180.1    1140.9    1048.2    1029.3    1017.0    1013.4    978.27
  955.97    881.21    862.92    750.14    696.35    668.01    616.30    515.79    424.94    416.93
  393.27    204.67
ZeroEnergy[kcal/mol]    87.142241254333863
 ElectronicLevels[1/cm]                 1
  0.0000000000000000       1.0000000000000000
End
!********************************************
RRHO          !          12
Geometry[angstrom]         13
C  -1.27364  0.01085  -1.15648
C  -1.27268  0.01090   0.23760
C  -0.06407  0.01085  -1.85660
H  -2.21610  0.02040  -1.69156
C  -0.00360 -0.00532   0.76905
H  -2.18598  0.03196   0.81948
C   1.16322  0.01338  -1.19132
H  -0.08836  0.01521  -2.94193
C   1.22958  0.00188   0.21328
O   0.23725  0.01339   2.29384
H   2.07428  0.02400  -1.78422
H   1.24879  0.03993   2.04679
H   0.03545 -0.85051   2.68192
        Core     RigidRotor
      SymmetryFactor   0.50000000000000000
End
   Frequencies[1/cm]         32
  3783.4    3217.2    3202.0    3183.7    3157.7    2600.3    1678.1    1599.3    1517.0    1489.9
  1446.2    1347.5    1291.0    1180.0    1141.0    1048.1    1026.2    1016.0    1011.5    976.48
  955.58    881.09    876.44    749.76    695.64    668.48    615.52    510.97    423.08    415.78
  389.79    203.28
ZeroEnergy[kcal/mol]    87.233147721156224
```

ElectronicLevels[1/cm] 1
0.0000000000000000 1.0000000000000000
End
!***
RRHO ! 13
Geometry[angstrom] 13
C -1.27386 0.01084 -1.15631
C -1.27179 0.01089 0.23775
C -0.06460 0.01088 -1.85721
H -2.21670 0.02040 -1.69070
C -0.00184 -0.00518 0.76749
H -2.18461 0.03211 0.82039
C 1.16287 0.01329 -1.19251
H -0.08948 0.01521 -2.94256
C 1.23061 0.00186 0.21216
O 0.23568 0.01327 2.29615
H 2.07364 0.02379 -1.78599
H 1.24509 0.04234 2.05993
H 0.03467 -0.85157 2.68251
Core RigidRotor
SymmetryFactor 0.50000000000000000
End
Frequencies[1/cm] 32

3784.1	3217.1	3201.9	3183.4	3156.9	2655.4	1678.5	1598.9	1526.1	1489.9
1445.6	1348.0	1290.8	1179.8	1141.0	1047.9	1023.7	1015.8	1008.9	974.87
955.26	888.11	880.94	749.46	695.02	668.11	614.77	506.80	421.77	414.18
386.30	202.19								

ZeroEnergy[kcal/mol] 87.296865184171972
ElectronicLevels[1/cm] 1
0.0000000000000000 1.0000000000000000
End
!***
RRHO ! 14
Geometry[angstrom] 13
C -1.27405 0.01083 -1.15613
C -1.27084 0.01093 0.23788
C -0.06517 0.01085 -1.85782
H -2.21730 0.02042 -1.68980
C -0.00004 -0.00506 0.76593
H -2.18315 0.03227 0.82133
C 1.16247 0.01323 -1.19373
H -0.09066 0.01520 -2.94315
C 1.23168 0.00188 0.21104
O 0.23405 0.01312 2.29846
H 2.07291 0.02374 -1.78778
H 1.24157 0.04443 2.07277
H 0.03324 -0.85237 2.68346
Core RigidRotor

SymmetryFactor 0.50000000000000000
End
Frequencies[1/cm] 32

3784.6	3217.0	3201.8	3183.3	3156.4	2708.3	1678.9	1598.4	1534.2	1489.8
1445.0	1348.4	1290.7	1179.7	1141.1	1047.9	1021.8	1015.6	1006.0	973.38
954.93	899.19	880.81	749.15	694.43	667.27	614.00	502.92	420.86	411.94
382.72	201.24								

ZeroEnergy[kcal/mol] 87.362454660839058
ElectronicLevels[1/cm] 1
0.0000000000000000 1.0000000000000000
End
!***
RRHO ! 15
Geometry[angstrom] 13
C -1.27425 0.01082 -1.15593
C -1.26982 0.01097 0.23802
C -0.06576 0.01081 -1.85844
H -2.21793 0.02043 -1.68885
C 0.00179 -0.00493 0.76438
H -2.18159 0.03245 0.82232
C 1.16209 0.01318 -1.19498
H -0.09187 0.01517 -2.94376
C 1.23278 0.00192 0.20991
O 0.23233 0.01297 2.30082
H 2.07217 0.02368 -1.78965
H 1.23819 0.04652 2.08506
H 0.03174 -0.85321 2.68438
Core RigidRotor
SymmetryFactor 0.50000000000000000
End
Frequencies[1/cm] 32

3785.2	3216.9	3201.7	3183.2	3155.9	2757.3	1679.3	1598.0	1541.5	1489.8
1444.5	1348.8	1290.4	1179.6	1141.1	1047.9	1020.2	1015.5	1003.0	971.96
954.64	907.28	880.67	748.87	693.90	666.10	613.19	499.40	420.28	409.20
379.06	200.42								

ZeroEnergy[kcal/mol] 87.411347464931277
ElectronicLevels[1/cm] 1
0.0000000000000000 1.0000000000000000
End
!***
RRHO ! 16
Geometry[angstrom] 13
C -1.27445 0.01080 -1.15572
C -1.26876 0.01102 0.23817
C -0.06636 0.01076 -1.85906
H -2.21857 0.02044 -1.68786
C 0.00365 -0.00480 0.76284
H -2.17996 0.03265 0.82336

```
C   1.16172  0.01312  -1.19626
H   -0.09310  0.01511  -2.94438
C   1.23391  0.00196  0.20878
O   0.23055  0.01281  2.30322
H   2.07141  0.02363  -1.79158
H   1.23490  0.04862  2.09691
H   0.03022  -0.85409  2.68526
        Core     RigidRotor
      SymmetryFactor   0.50000000000000000
End
  Frequencies[1/cm]        32
 3785.8    3216.8    3201.7    3183.1    3155.4    2803.2    1679.6    1597.5    1548.1    1489.6
 1444.0    1349.1    1290.1    1179.4    1141.1    1047.8    1018.7    1015.5    999.80    970.68
 954.39    911.40    880.52    748.61    693.39    664.73    612.33    496.18    419.92    406.10
 375.33    199.68
ZeroEnergy[kcal/mol]    87.448817990836526
 ElectronicLevels[1/cm]              1
 0.0000000000000000       1.0000000000000000
End
!*******************************************
RRHO          !          17
Geometry[angstrom]        13
C   -1.27464  0.01078  -1.15550
C   -1.26766  0.01108  0.23832
C   -0.06698  0.01070  -1.85970
H   -2.21922  0.02045  -1.68683
C   0.00552  -0.00466  0.76131
H   -2.17827  0.03287  0.82443
C   1.16135  0.01306  -1.19757
H   -0.09436  0.01504  -2.94501
C   1.23504  0.00201  0.20764
O   0.22871  0.01264  2.30565
H   2.07065  0.02357  -1.79355
H   1.23169  0.05075  2.10843
H   0.02870  -0.85502  2.68609
        Core     RigidRotor
      SymmetryFactor   0.50000000000000000
End
  Frequencies[1/cm]        32
 3786.6    3216.8    3201.6    3183.1    3155.0    2846.4    1679.9    1597.0    1553.9    1489.5
 1443.5    1349.3    1289.7    1179.3    1141.1    1047.8    1017.5    1015.4    996.53    969.60
 954.14    913.08    880.38    748.36    692.92    663.22    611.48    493.21    419.67    402.71
 371.51    199.02
ZeroEnergy[kcal/mol]    87.472179369012773
 ElectronicLevels[1/cm]              1
 0.0000000000000000       1.0000000000000000
End
!*******************************************
```

RRHO ! 18
Geometry[angstrom] 13
C -1.27483 0.01077 -1.15528
C -1.26654 0.01115 0.23848
C -0.06760 0.01064 -1.86034
H -2.21987 0.02046 -1.68578
C 0.00740 -0.00452 0.75980
H -2.17654 0.03309 0.82553
C 1.16100 0.01300 -1.19890
H -0.09564 0.01495 -2.94563
C 1.23618 0.00207 0.20649
O 0.22684 0.01246 2.30811
H 2.06989 0.02350 -1.79555
H 1.22854 0.05293 2.11969
H 0.02720 -0.85599 2.68688
Core RigidRotor
SymmetryFactor 0.50000000000000000
End
Frequencies[1/cm] 32
3787.4 3216.7 3201.6 3183.0 3154.6 2887.3 1680.2 1596.6 1559.4 1489.3
1443.0 1349.5 1289.3 1179.1 1141.0 1047.7 1016.5 1015.3 993.44 968.69
953.93 914.41 880.24 748.13 692.47 661.63 610.66 490.48 419.50 399.11
367.62 198.40
ZeroEnergy[kcal/mol] 87.498570713948192
ElectronicLevels[1/cm] 1
0.0000000000000000 1.0000000000000000
End
!**
RRHO ! 19
Geometry[angstrom] 13
C -1.27502 0.01075 -1.15504
C -1.26539 0.01123 0.23864
C -0.06823 0.01056 -1.86098
H -2.22053 0.02047 -1.68471
C 0.00928 -0.00436 0.75829
H -2.17477 0.03333 0.82665
C 1.16066 0.01295 -1.20024
H -0.09693 0.01484 -2.94626
C 1.23732 0.00214 0.20533
O 0.22493 0.01226 2.31059
H 2.06913 0.02343 -1.79758
H 1.22545 0.05515 2.13067
H 0.02574 -0.85701 2.68763
Core RigidRotor
SymmetryFactor 0.50000000000000000
End
Frequencies[1/cm] 32
3788.4 3216.7 3201.5 3182.9 3154.3 2926.1 1680.6 1596.1 1564.6 1489.2

1442.5 1349.8 1288.9 1178.9 1140.9 1047.7 1015.8 1015.0 990.73 967.89
953.76 916.08 880.09 747.91 692.04 659.97 609.87 487.97 419.39 395.32
363.69 197.86
ZeroEnergy[kcal/mol] 87.523232277080547
ElectronicLevels[1/cm] 1
0.0000000000000000 1.0000000000000000
End
!***
RRHO ! 20
Geometry[angstrom] 13
C -1.27521 0.01073 -1.15480
C -1.26423 0.01132 0.23880
C -0.06886 0.01047 -1.86163
H -2.22119 0.02047 -1.68362
C 0.01116 -0.00420 0.75678
H -2.17297 0.03359 0.82779
C 1.16033 0.01289 -1.20160
H -0.09823 0.01470 -2.94689
C 1.23846 0.00222 0.20417
O 0.22299 0.01206 2.31311
H 2.06837 0.02335 -1.79964
H 1.22239 0.05741 2.14135
H 0.02434 -0.85808 2.68835
Core RigidRotor
SymmetryFactor 0.50000000000000000
End
Frequencies[1/cm] 32
3789.3 3216.7 3201.5 3182.9 3153.9 2962.6 1681.0 1595.6 1569.5 1489.0
1442.0 1350.0 1288.5 1178.7 1140.9 1047.7 1015.5 1014.5 988.34 967.13
953.64 917.30 879.95 747.71 691.63 658.27 609.09 485.66 419.30 391.39
359.72 197.36
ZeroEnergy[kcal/mol] 87.538402209197765
ElectronicLevels[1/cm] 1
0.0000000000000000 1.0000000000000000
End
!***
RRHO ! 21
Geometry[angstrom] 13
C -1.27539 0.01072 -1.15455
C -1.26306 0.01142 0.23897
C -0.06950 0.01037 -1.86228
H -2.22185 0.02047 -1.68252
C 0.01304 -0.00403 0.75527
H -2.17115 0.03386 0.82895
C 1.16001 0.01282 -1.20297
H -0.09954 0.01454 -2.94753
C 1.23960 0.00231 0.20300
O 0.22103 0.01185 2.31567

```
H   2.06762  0.02327  -1.80172
H   1.21936  0.05971   2.15176
H   0.02303  -0.85921   2.68903
        Core     RigidRotor
      SymmetryFactor   0.5000000000000000
End
   Frequencies[1/cm]         32
 3790.4     3216.7     3201.5     3182.9     3153.6     2997.0     1681.3     1595.2     1573.9     1488.9
 1441.5     1350.2     1288.0     1178.6     1140.8     1047.7     1015.4     1013.8     986.11     966.33
 953.48     917.34     879.81     747.51     691.23     656.55     608.30     483.55     419.25     387.34
 355.74     196.90
ZeroEnergy[kcal/mol]    87.555172536336125
 ElectronicLevels[1/cm]                 1
 0.0000000000000000       1.0000000000000000
End
!*********************************************
    Tunneling    Eckart
    ImaginaryFrequency[1/cm]    393.64900000000000
    WellDepth[kcal/mol]        87.14
    WellDepth[kcal/mol]        14.36
End
End
!*********************************************
Barrier TS12 W2 W7
RRHO          !          11
 Geometry[angstrom]         13
 C   0.00000   0.00000   0.00000
 C   0.00000   0.00000   1.50594
 C   1.15094   0.00000   2.22590
 C   2.41601  -0.04528   1.58951
 C   2.49880  -0.07758   0.17831
 C   1.38324  -0.08410  -0.59824
 O  -0.61890   1.12657  -0.52438
 H   3.47512  -0.10275  -0.29410
 H  -0.61017  -0.84367  -0.38352
 H  -0.96743   0.05344   1.99292
 H   1.10694   0.04319   3.30898
 H   3.32142  -0.03802   2.18376
 H   1.44112  -0.09017  -1.68015
        Core     RigidRotor
      SymmetryFactor    1.0000000000000000
End
   Frequencies[1/cm]         32
 3220.1     3211.8     3204.5     3187.0     3183.8     2893.4     1618.8     1557.2     1449.7     1389.1
 1341.5     1279.5     1203.0     1185.7     1119.3     1095.1     1051.6     1015.0     995.24     988.48
 980.38     940.79     823.41     801.04     743.66     657.05     577.19     566.67     528.02     396.27
 308.11     122.09
ZeroEnergy[kcal/mol]    88.2
```

```
 ElectronicLevels[1/cm]                  1
  0.0000000000000000       1.0000000000000000
 End
!********************************************
     Tunneling    Eckart
     ImaginaryFrequency[1/cm]    533.85350000000005
     WellDepth[kcal/mol]         43.4
     WellDepth[kcal/mol]         70.4
 End
End
!********************************************
```

REFERENCES

(1) Lee, Y. T. Molecular Beam Studies of Elementary Chemical Processes. *Science* **1986,** *236*, 793-798.

(2) Casavecchia, P. Chemical Reaction Dynamics with Molecular Beams. *Rep. Prog. Phys.* **2000**, 63, 355-414.

(3) Casavecchia, P.; Leonori, F.; Balucani, N.; Petrucci, R.; Capozza, G,; Segoloni, E. Probing the Dynamics of Polyatomic Multichannel Elementary Reactions by Crossed Molecular Beam Experiments with Soft Electron-Ionization Mass Spectrometric Detection. *Phys. Chem. Chem. Phys.* **2009**, *11*, 46-65.

(4) Leonori F.; Balucani, N.; Capozza, G.; Segoloni, E. Volpi, G.G.; Casavecchia, P. Dynamics of the O(^{3}P) + C_2H_2 Reaction from Crossed Molecular Beam Experiments with Soft Electron Ionization Detection. *Phys. Chem. Chem. Phys.* **2014**, *16*, 10008-22.

(5) Alagia, M.; Balucani, N.; Casavecchia, P.; Stranges, D.; Volpi, G.G. Reactive Scattering of Atoms and Radicals. *J. Chem. Soc. Faraday Trans*. **1995**, *91*, 575-596.

(6) Casavecchia, P.; Leonori, F.; Balucani, N. Reaction Dynamics of Oxygen Atoms with Unsaturated Hydrocarbons from Crossed Molecular Beam Studies: Primary Products, Branching Ratios and Role of Intersystem Crossing. *Int. Rev. Phys. Chem.* **2015**, *34*, 161-204.

(7) Daly, N. R., Scintillation Type Mass Spectrometer Ion Detector. *Rev. Sci. Instrum.* **1960**, *31*, 264-267.

(8) Alagia, M.; Aquilanti, V.; Ascenzi, D.; Balucani, N.; Cappelletti, D.; Cartechini, L.; Casavecchia, P.; Pirani, F.; Sanchini, G.; Volpi, G. G. Magnetic Analysis of Supersonic Beams of Atomic Oxygen, Nitrogen, and Chlorine Generated from a Radio-Frequency Discharge. *Isr. J. Chem*. **1997**, *37*, 329-342.

(9) Sibener, S. J.; Buss, R. J.; Ng, C. Y.; Lee, Y. T. Development of a Supersonic O(^3P_J), O($^1D_2$) Nozzle Beam Source. *Rev. Sci. Instrum.* **1980**, *51*, 167−182

(10) NIST Chemistry WebBook.

(11) Caracciolo, A.; Vanuzzo, G.; Balucani, N.; Minton, T. K.; Casavecchia, P.; Pratali Maffei, L.; Cavallotti, C. Crossed Beams and Theoretical Studies of the O(^{3}P,^{1}D) + Benzene Reaction Dynamics: Primary Product, Branching Fractions and Role of Intersystem Crossing. (Work in progress).

(12) Miller, W. B.; Safron, S.A.; Herschbach, D.R. Exchange Reactions of Alkali Atoms with Alkali Halides: a Collision Complex Mechanism. *Discuss. Faraday Soc.* **1967**, *44*, 108-122

(13) Sibener, S. J.; Buss, R. J.; Casavecchia, P.; Hirooka, T.; Lee, Y. T., A Crossed Molecular Beams Investigation of the Reactions O(^{3}P)+C_6H_6, C_6D_6. *J. Chem. Phys.* **1980**, *72*, 4341-4349.

(14) Schmoltner, A.M.; Chu, P.M.; Brudzynski, R.J.; Lee, Y.T. Crossed Molecular Beam Study of the Reaction O(^{3}P) + C_2H_4. *J. Chem. Phys.* **1989**, *91*, 6926-6936.

(15) Caracciolo, A.; Vanuzzo, G.; Balucani, N.; Stranges, D.; Pratali Maffei, L.; Cavallotti, C.; Casavecchia, P. Combined Experimental and Theoretical Studies of the O(^{3}P) + 1-Butene Reaction Dynamics: Primary Products, Branching Ratios and Role of Intersystem Crossing. *J. Phys. Chem. A* **2019**, *123*, 9934-9956.

(16) Chen, H.-F.; Liang, C.-W.; Lin, J. J.; Lee, Y.-P.; Ogilvie, J. F.; Xu, Z. F.; Lin, M. C., Dynamics of Reactions O(^{1}D)+C_6H_6 and C_6D_6. *J. Chem. Phys.* **2008,** *129*, 174303.

(17) Pratali Maffei L., Pelucchi, M., Faravelli, T., Cavallotti C. Theoretical Study of Sensitive Reactions in Phenol Decomposition. *React. Chem. Eng.*, **2020**, *5*, 452-472.

(18) Leonori, F.; Balucani, N.; Nevrly, V.; Bergeat, A.; Falcinelli, S.; Vanuzzo, G.; Casavecchia, P.; Cavallotti, C. Experimental and Theoretical Studies on the Dynamics of the O(^{3}P) + Propene Reaction: Primary Products, Branching Ratios, and Role of Intersystem Crossing. *J. Phys. Chem. C* **2015**, *119*, 14632–14652.
